# CircKIAA1617 promotes stemness via USP14/PGRMC1-mediated autophagy and lipid metabolism reprogramming in ER-positive breast cancer

**DOI:** 10.1186/s12943-026-02580-2

**Published:** 2026-01-31

**Authors:** Jingwen Yang, Yaming Li, Zekun Wang, Yuhan Sun, Yinqiao He, Tong Niu, Yiran Liang, Xi Chen, Tong Chen, Dianwen Han, Ning Zhang, Wenjing Zhao, Bing Chen, Lijuan Wang, Dan Luo, Xiaoyan Li, Qifeng Yang

**Affiliations:** 1 Department of Breast Surgery, General Surgery, Qilu Hospital of Shandong University, Wenhua Xi Road, No. 107, Jinan, 250012 Shandong PR China; 2 Biological Resource Center, Qilu Hospital of Shandong University, Jinan, 250012 Shandong PR China; 3 Research Institute of Breast Cancer, Shandong University, Jinan, 250012 Shandong PR China

**Keywords:** ER-positive breast cancer, CircKIAA1617, Autophagy, Stemness, Lipid metabolism reprogramming

## Abstract

**Background:**

Breast cancer (BC) is the most common neoplasm in women, and its growth mainly depends on estrogen, but the mechanism of estrogen in BC is still not fully understood. Circular RNAs (circRNAs) represent a novel type of regulatory RNA characterized by high evolutionary conservation and stability. This study aimed to investigate the roles and mechanisms of circRNAs in ER-positive BC.

**Methods:**

CircKIAA1617 was identified through high-throughput RNA sequencing in ER-positive BC. Gain- and loss-of-function assays were performed to evaluate the functions of circKIAA1617 in ER-positive BC cells. Chromatin immunoprecipitation (ChIP) and luciferase assays verified the regulatory effects of estrogen on circKIAA1617 expression. RNA pulldown experiments, proteomic analyses, and RNA immunoprecipitation were conducted to identify the downstream targets of circKIAA1617.

**Results:**

CircKIAA1617 expression was upregulated in ER-positive BC cells and tissues, indicating an unfavorable prognosis. In vitro and in vivo studies proved the circKIAA1617 increased the proliferation and stemness of ER-positive BC cells by inducing autophagy. Mechanistically, circKIAA1617 was activated by estrogen and cyclized by EIF4A3. Moreover, circKIAA1617 could act as a scaffold to enhance the interaction between the PGRMC1 and USP14 proteins, further increasing the stability of the PGRMC1 protein by decreasing its K48-linked polyubiquitination at lysine 105. In addition, autophagy activated by the circKIAA1617/USP14/PGRMC1 axis further modulated lipid metabolic reprogramming in ER-positive BC by increasing lipophagy, which accounted for the proliferation, stemness and autophagy of ER-positive BC.

**Conclusions:**

Our results revealed that circKIAA1617 promoted the proliferation and stemness of BC cells by regulating USP14/PGRMC1-mediated autophagy and lipid metabolic reprogramming and could serve as a potential diagnostic biomarker for ER-positive BC.

**Supplementary Information:**

The online version contains supplementary material available at 10.1186/s12943-026-02580-2.

## Background

Breast cancer (BC) is a growing global health threat, with approximately 2.26 million new cases and 680 thousand deaths reported worldwide each year, representing a significant threat to the lives and well-being of women [1]. In terms of the expression of estrogen receptor (ER), progesterone receptor (PR), and human epithelial growth factor receptor 2 (HER2), breast cancer can be classified into four molecular subtypes: luminal A, luminal B, HER2-enriched, and basal‐like. Among them, estrogen receptor-positive breast cancer is the most common subtype, accounting for more than 70% of all breast cancer cases, and generally progresses in an ER-positive manner [2]. By binding to estrogen receptors (ERs), estrogen (17‐β‐estradiol, E2) serves as a critical driver of ER-positive BC, triggering the aberrant expression of numerous target genes that govern tumor proliferation, metastasis, stemness, and metabolism [3–5]. Although the classical mechanisms by which estrogen affects the malignant behaviors of ER-positive BC have been extensively studied, additional regulatory mechanisms, particularly those involving novel non-coding RNAs, may yet be identified to provide new insights for therapeutic development.

Circular RNAs (circRNAs), which are endogenous single-stranded and covalently closed circular RNAs, were first identified in 1976 [6]. Unlike canonical RNAs, circRNAs are generated by backsplicing, in which the 3’ splice donor site of an exon is covalently joined to a 5’ splice acceptor of the same or upstream exon, resulting in the formation of a circular RNA structure [7]. Due to their unique closed-loop structure, circular RNAs exhibit increased stability, higher conservation across species, and increased tolerance to RNase R degradation compared with linear RNA [8]. CircRNAs exert their effects through different mechanisms, such as interactions with microRNAs or proteins and translation into novel peptides [9]. Previous studies have proved that circRNAs can act as a class of bona fide functional molecules, providing insights into the mechanism of cancer progression. For example, circPGR functions as a competing endogenous RNA (ceRNA) to sponge miR-301a-5p and suppress ER-positive breast cancer cell growth [10]. Du et al. verified that circ-Foxo3 bound p53 and the E3 ubiquitin ligase MDM2, thereby promoting MDM2-induced p53 ubiquitination and subsequent degradation to influence the apoptosis of breast cancer cells [11]. However, the effects of estrogen on circRNA expression and the underlying roles and mechanisms have not been fully evaluated, and the identification of estrogen-responsive circRNAs and an investigation of their molecular mechanisms could provide novel tailored targets for patients with ER-positive BC.

In the present study, we identified an estrogen-responsive circRNA termed circKIAA1617 (circBase ID: hsa_ circ_0017636 [12]) in ER-positive BC whose expression was significantly upregulated in ER-positive BC tissues and exhibited prognostic significance. In vitro and in vivo studies proved that circKIAA1617 promoted the proliferation and stemness of ER-positive BC cells by inducing autophagy. Mechanistically, circKIAA1617 could be activated by estrogen and further cyclized by eukaryotic initiation factor 4A3 (EIF4A3), a key regulator of mRNA splicing and translation [13]. Furthermore, circKIAA1617 could act as a scaffold to enhance the binding between progesterone receptor membrane component 1 (PGRMC1, which is known to participates in the regulation of autophagy) and ubiquitin-specific peptidase 14 (USP14, a deubiquitinating enzyme) [14], further inhibiting the K48-linked polyubiquitination of lysine 105 of the PGRMC1 protein and blocking its proteasome degradation, which accounted for the proliferation, stemness and autophagy of ER-positive BC. Moreover, we demonstrated that the autophagy induced by the circKIAA1617/USP14/PGRMC1 axis enhanced lipophagy to modulate lipid metabolic reprogramming in ER-positive BC. In conclusion, our study revealed the roles and mechanisms of estrogen-responsive circKIAA1617 in ER-positive BC, and it shows promise as a potential diagnostic biomarker for the clinical intervention of ER-positive BC.

## Materials and methods

### Ethics statement and human tissue samples

Approval for all experimental protocols was secured through the Institutional Review Board of Shandong University Qilu Hospital, with written informed consent prospectively obtained from participant (Approval Number: KYLL-2022(ZM)-1058). Breast cancer tissue specimens were obtained from the Shandong University Qilu Hospital by prospective collection. Resected specimens underwent independent histopathological validation through triple-blinded consensus review by board-certified surgical pathologists, followed by immediate cryopreservation at -80 ℃. Clinicopathological data were retrieved from the institutional medical records. ER and PR status were determined by immunohistochemistry (IHC) on formalin-fixed, paraffin-embedded specimens, and HER2 status was assessed by IHC with fluorescence in situ hybridization (FISH) for cases with equivocal IHC results, following institutional laboratory protocols. Total RNA was extracted from tumor tissues and circKIAA1617 expression was quantified by qRT-PCR with normalization to endogenous controls. Patients were dichotomized into high- and low-expression groups using the median circKIAA1617 expression value as the cutoff. Survival analyses were performed using overall survival (OS) as the endpoint.

### Cell culture and treatments

All cell lines were procured from the American Type Culture Collection (Manassas, VA, USA) and routinely maintained in standard media and conditions. MCF7 (RRID: CVCL_0031), T47D (RRID: CVCL_0553), and HEK293T (RRID: CVCL_0063) cells were cultured in Dulbecco’s Modified Eagle Medium (DMEM; Invitrogen, Carlsbad, CA, USA) supplemented with 10% fetal bovine serum (FBS; Gibco, MI, USA), 100 U/ml penicillin (Macgene, Beijing, China) and 100 µg/ml streptomycin (Macgene, Beijing, China). Cell cultures were incubated at 37 °C in a 5% CO_2_-humidified atmosphere. For experiments including the treatment of E2, cells were pre-treated with E2-free medium which contains charcoal-stripped FBS and phenol-red-free medium for 24 h.

### Animal experiments

For xenograft proliferation studies, MCF7 overexpressing pLO5 and circKIAA1617 cells (1 × 10^6^ cells) in 200µL of PBS containing Matrigel (1:3, v/v) was injected subcutaneously into the left flank of 4 to 6-week-old BALB/c nude female mice (*n* = 5 for each group). The day before MCF7 cell transplantation, mice were subcutaneously implanted with 17β-estradiol control release pellets (SE-121, Innovative Research of America). Tumor growth rate was monitored by measuring tumor diameters every 5 days. At the endpoint, the mice were sacrificed, and the xenografted tumors were measured and weighed. Hematoxylin and eosin (H&E) and immunohistochemistry (IHC) staining were performed on the sections. For in vivo limiting dilution assay, 1 × 10^5^, 2.5 × 10^5^, 5 × 10^5^, 1 × 10^6^ control or circKIAA1617-OV MCF7 cells were injected subcutaneously into 4 to 6-week-old BALB/c nude female mice. Animals were randomly assigned to experimental groups, and investigators were blinded to group allocation during data collection and analysis. All the animal experimental procedures conducted in this study received approval from the Ethical Committee of Shandong University (Approval Number: DWLL-202500074).

### Statistical analysis

Statistical analysis was conducted by GraphPad Prism 10.1.2 and SPSS 25.0. All data were represented as mean ± standard deviation (SD) and are derived from a minimum of three independent experiments. Student’s t-test or one-way ANOVA was utilized to evaluate the relationship between parametric variables. Chi-square test was applied to analyze the relationships between nonparametric variables. Kaplan-Meier analysis was used to analyze the survival differences. *P* < 0.05 was regarded statistically significant.

## Results

### CircKIAA1617 expression is upregulated in ER-positive BC

**Fig. 1:**
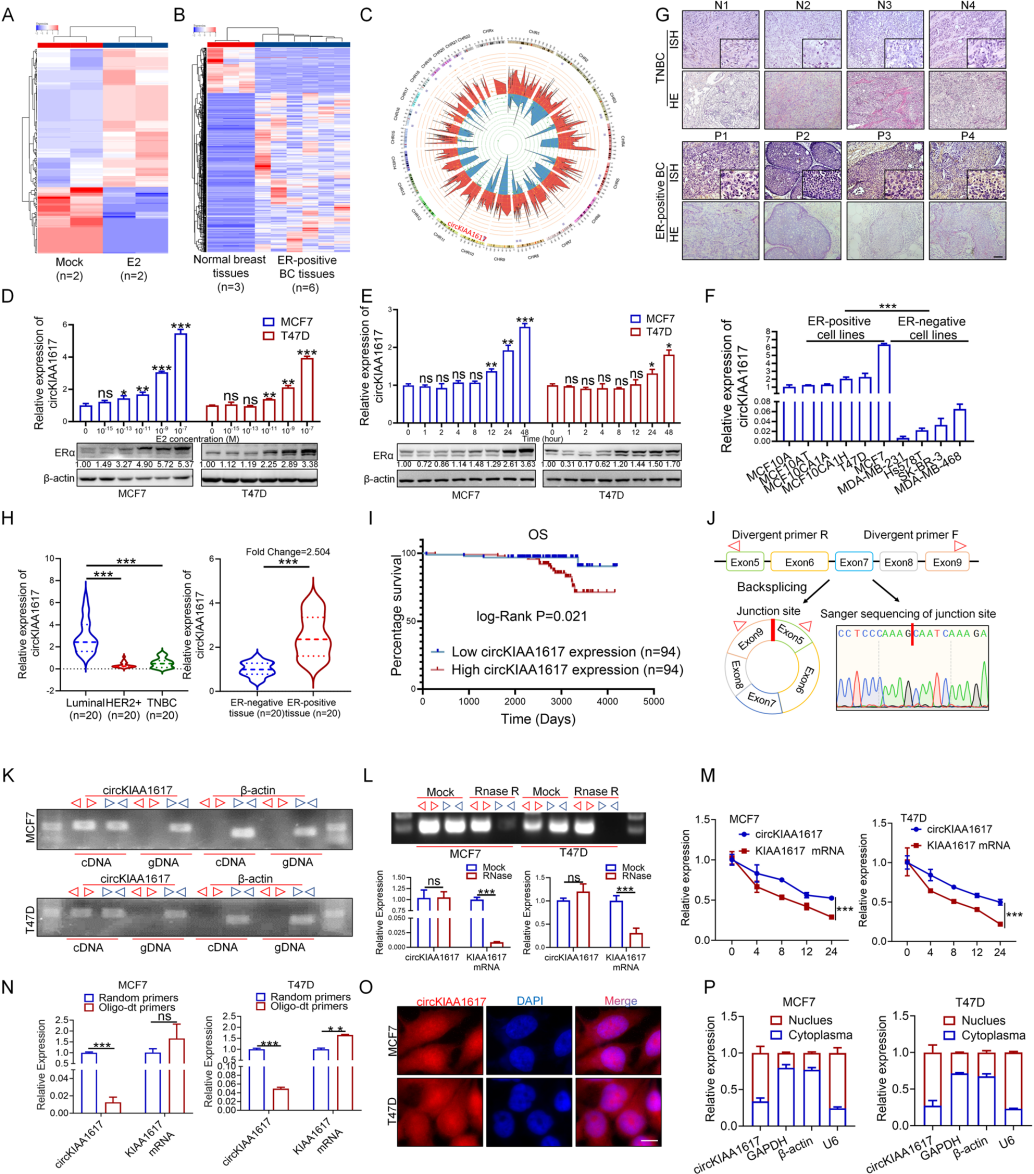
Identification of circKIAA1617 as an estrogen-responsive circRNA in ER-positive BC. **A** Transcriptome sequencing of the MCF7 cell line treated with or without E2 (1 nM). **B** Transcriptome sequencing of normal breast tissues and ER-positive BC tissues. **C** Circos plot indicating the differentially expressed circRNAs. The innermost circle and second circle show the dysregulated circRNAs obtained from the results of the two experiments described above. The third circle (purple squares) represents the circRNAs whose expression changed significantly in the results from the two experiments described above. The outermost circle shows the chromosomal distribution of the circRNAs. **D** The expression levels of circKIAA1617 and ERα in ER-positive BC cells treated with different concentrations of estrogen (*n* = 3). **E** The expression levels of circKIAA1617 and ERα in ER-positive BC cells treated with estrogen for different durations (*n* = 3). **F** Expression of circKIAA1617 in breast cancer cell lines (*n* = 3). **G** ISH analysis of circKIAA1617 expression in ER-negative (N1-4) and ER-positive (P1-4) breast cancer tissues. Scale bars = 100 μm. **H** Expression of circKIAA1617 in different subtypes of breast cancer. **I** Kaplan-Meier curves and log-rank tests comparing overall survival (OS) between the high-circKIAA1617-expressing subgroup and low-circKIAA1617-expressing subgroup (*n* = 94 patients per group) in an enrolled cohort of patients with ER-positive BC. **J** Upper panel: Schematic illustration indicating that circKIAA1617 is a circularized sequence composed of exons 5–9 of human KIAA1617. Lower panel: Sequencing analysis of the head-to-tail splicing junction of circKIAA1617. **K** CircKIAA1617, along with β-actin, were amplified from the cDNA or gDNA of MCF7 and T47D cells with divergent and convergent primers, respectively (*n* = 3). **L** qRT-PCR assays were performed to assess the expression of circKIAA1617 and the KIAA1617 mRNA after treatment with or without RNase R (*n* = 3). **M** qRT-PCR analysis of the stability of circKIAA1617 and linear KIAA1617 mRNA following actinomycin-D treatment (*n* = 3). **N** Random 6-mer primers or oligo (dT) primers were used for the reverse transcription of cDNA, and the levels of circKIAA1617 and the KIAA1617 mRNA amplified by those two different primer sets were analyzed using qRT-PCR (*n* = 3). **O** Subcellular spatial map of circKIAA1617 localization obtained using fluorescence in situ hybridization (FISH) with a junction-specific probe (*n* = 3). Scale bars = 10 μm. **P** Expression of circKIAA1617 in the cytoplasm and nucleus of MCF7 and T47D cells (*n* = 3). ns, not significant; *P < 0.05; **P < 0.01; and ***P < 0.001

CircRNA-seq was initially performed to screen for potential estrogen-responsive circRNAs associated with the estrogen-induced progression of ER-positive BC. As shown in Fig. 1. A, circRNAs that were dysregulated in E2-treated MCF7 cells were first analyzed, and the results revealed 97 upregulated circRNAs and 202 downregulated circRNAs in E2-stimulated ER-positive BC cells. In parallel, circRNAs that have been reported to be differentially expressed between normal breast tissues and ER-positive BC tissues were further screened to identify potential oncogenic circRNAs [15]. As shown in Fig. 1. B, we identified 518 upregulated circRNAs and 149 downregulated circRNAs in ER-positive BC tissues. We subsequently intersected the aforementioned results, and 21 circRNAs that were dysregulated in both E2-stimulated ER-positive BC cells and tissues were further identified (Fig. 1. C, Figure S1. A). Among the 21 circRNAs, the top 5 dysregulated circRNAs upon E2 treatment detected using RNA-seq were further selected, and detailed information on the circRNAs is shown in Figure S1. B. As shown in Figure S1. C, D, qRT-PCR assays revealed that circKIAA1617 was the only circRNA whose expression was upregulated in both E2-treated ER-positive BC cell lines and ER-positive BC tissues at the most significant level. Although the magnitude of the fold change differed between the circRNA-seq screening and the qRT-PCR validation, the upregulation trend of circKIAA1617 remained consistent. This quantitative discrepancy is commonly observed and likely attributable to the inherent differences in sensitivity and dynamic range between circRNA-seq screening and the qRT-PCR validation. Crucially, the qRT-PCR results, which serve as the gold standard for quantification, confirmed the significant overexpression of circKIAA1617 identified by the initial screening. Thus, this circRNA was selected for further study. Moreover, E2 was used to treat ER-positive BC cells in a concentration- and time-dependent manner, further indicating that circKIAA1617 is an estrogen-responsive circRNA (Fig. 1. D, E). Furthermore, an ER-specific siRNA and the ER antagonist fulvestrant were also used to antagonize the effects of ER, and the results showed that the regulatory effects of E2 on circKIAA1617 were suppressed upon ER inhibition (Figure S1. E-H). In addition, E2 was also used to treat TNBC (Triple-negative breast cancer) cell lines, and the dose and time course of E2 did not significantly affect circKIAA1617 expression in MDA-MB-231 cells, whereas the overexpression of ESR1 in MDA-MB-231 cells further led to the upregulation of circKIAA1617 expression upon E2 treatment, indicating that circKIAA1617 expression is E2 dependent (Figure S1. I-K). The detection of circKIAA1617 in breast cancer cell lines revealed that circKIAA1617 was significantly overexpressed in ER-positive BC cells (Fig. 1. F). Additionally, the expression of circKIAA1617 in breast cancer tissues was also examined using ISH and qRT-PCR, and the results further confirmed that circKIAA1617 was significantly overexpressed in ER-positive BC tissues (Fig. 1. G, H). A total of 188 patients with ER-positive BC, and the expression of circKIAA1617 was examined using qRT-PCR to explore the clinical significance of circKIAA1617; the results showed that circKIAA1617 expression was correlated with the tumor size, N status and Ki67 status in ER-positive BC (Table 1). The prognostic analysis revealed that circKIAA1617 expression was associated with shorter overall survival (OS) of patients with ER-positive BC (Fig. 1. I), and univariate and multivariate analyses further indicated that circKIAA1617 expression was an independent prognostic factor for the overall survival (OS) of patients with ER-positive BC (Table 2). Our data suggest that circKIAA1617 is an estrogen-induced circRNA that might play vital roles in ER-positive BC.

**Table 1:** Association between clinicopathological variables and circKIAA1617 expression in ER-positive BC patients

Variable	Cases(*n* = 188)	circKIAA1617 expression		*P* value
		Low (*n* = 94)	High (*n* = 94)	
**Age**				0.241
≤ 50		48	56	
>50		46	38	
**Histologic subtype**				0.519
**IDC**		83	80	
**other**		11	14	
**Histologic grade**				0.303
I + II		72	63	
III		15	19	
Unknown		7	12	
**Tumor Size**				**0.041**
≤ 2 cm		55	41	
>2 cm		39	53	
**N status**				**0.039**
Negative		61	47	
Positive		33	47	
**Distant Metastasis**				0.747
No + Unknown		90	88	
Yes		4	6	
**PR status**				0.468
Negative		8	11	
Positive		86	83	
**Her-2 status**				0.667
Negative		70	75	
Positive		8	7	
Unknown		16	12	
**Ki67 status**				**0.018**
Low		48	32	
High		46	62	

**Table 2:** Univariate and multivariate analyses of prognostic factors (OS) for patients with ER-positive BC

Variable	Univariate analysis (OS)		Multivariate analysis (OS)	
	HR (95% CI)	*P* value	HR (95% CI)	*P* value
**Age**				
≤ 50	Reference	-		
>50	2.414 (0.947–6.155)	0.065		
**Histologic subtype**				
IDC	Reference	-		
other	0.309 (0.041–2.320)	0.254		
**Histologic grade**				
I + II	Reference	-		
III	0.721 (0.207–2.513)	0.607		
Unknown	0.967 (0.219–4.258)	0.964		
**Tumor Size**				
≤ 2 cm	Reference	-		
>2 cm	1.094 (0.443–2.703)	0.846		
**N status**				
Negative	Reference	-		
Positive	2.107 (0.826–5.375)	0.119		
**Distant Metastasis**				
No + Unknown	Reference	-	Reference	-
Yes	**5.13 (1.469–17.920)**	**0.010**	**4.065 (1.159–14.260)**	**0.029**
**PR status**				
Negative	Reference	-		
Positive	0.407 (0.135–1.227)	0.110		
**Her-2 status**				
Negative	Reference	-		
Positive	0.689 (0.091–5.243)	0.719		
Unknown	2.335 (0.758–7.191)	0.139		
**Ki67 status**				
Low	Reference	-		
High	1.382 (0.524–3.645)	0.513		
**circKIAA1617 expression**				
Low	Reference	-	Reference	-
High	**3.388 (1.123–10.219)**	**0.030**	**3.077 (1.010–9.371)**	**0.048**

Annotations from the USCS Genome Browser revealed that circKIAA1617 originated from the 5th to 9th exons of KIAA1617 (Fig. 1. J). Specific divergent and convergent primers were designed to amplify circKIAA1617 and the linear KIAA1617 mRNA, and the specific joint sequence of head-to-tail splicing was detected in ER-positive BC cells by Sanger sequencing (Fig. 1. J). Moreover, PCR assays were performed to assess the expression of circKIAA1617 and actin using cDNA and genomic DNA (gDNA) templates from the MCF7 and T47D cell lines. Notably, while the convergent primers successfully amplified both circKIAA1617 and actin, the divergent primers specifically amplified circKIAA1617 from cDNA but not from gDNA (Fig. 1. K). RNase R was subsequently used to treat total RNA from ER-positive BC cells, and the results showed that compared with the KIAA1617 mRNA, circKIAA1617 was more resistant to digestion by RNase R, indicating the circular characteristics of circKIAA1617 (Fig. 1. L). In addition, actinomycin D assays were performed, confirming that circKIAA1617 was more stable than the linear mRNA of its parental host gene (Fig. 1. M). Total RNA from ER-positive BC cells was reverse-transcribed using either random primers or oligo (dT) primers to further validate the circular characteristics of circKIAA1617, and qRT-PCR revealed that compared with oligo (dT) primers, circKIAA1617 was clearly enriched in samples that were reverse transcribed using random primers, indicating the lack of a poly(A) tail in the circKIAA1617 sequence (Fig. 1. N). In addition, fluorescence in situ hybridization (FISH) assays and nuclear-cytoplasmic RNA extraction revealed that circKIAA1617 was located in both the cytoplasm and nucleus of MCF7 and T47D cells (Fig. 1. O, P). In conclusion, our results indicated that circKIAA1617 was an endogenously expressed estrogen-responsive circular RNA in ER-positive BC cells.

### CircKIAA1617 promoted the proliferation and stemness of ER-positive BC cells in vitro and in vivo

**Fig. 2:**
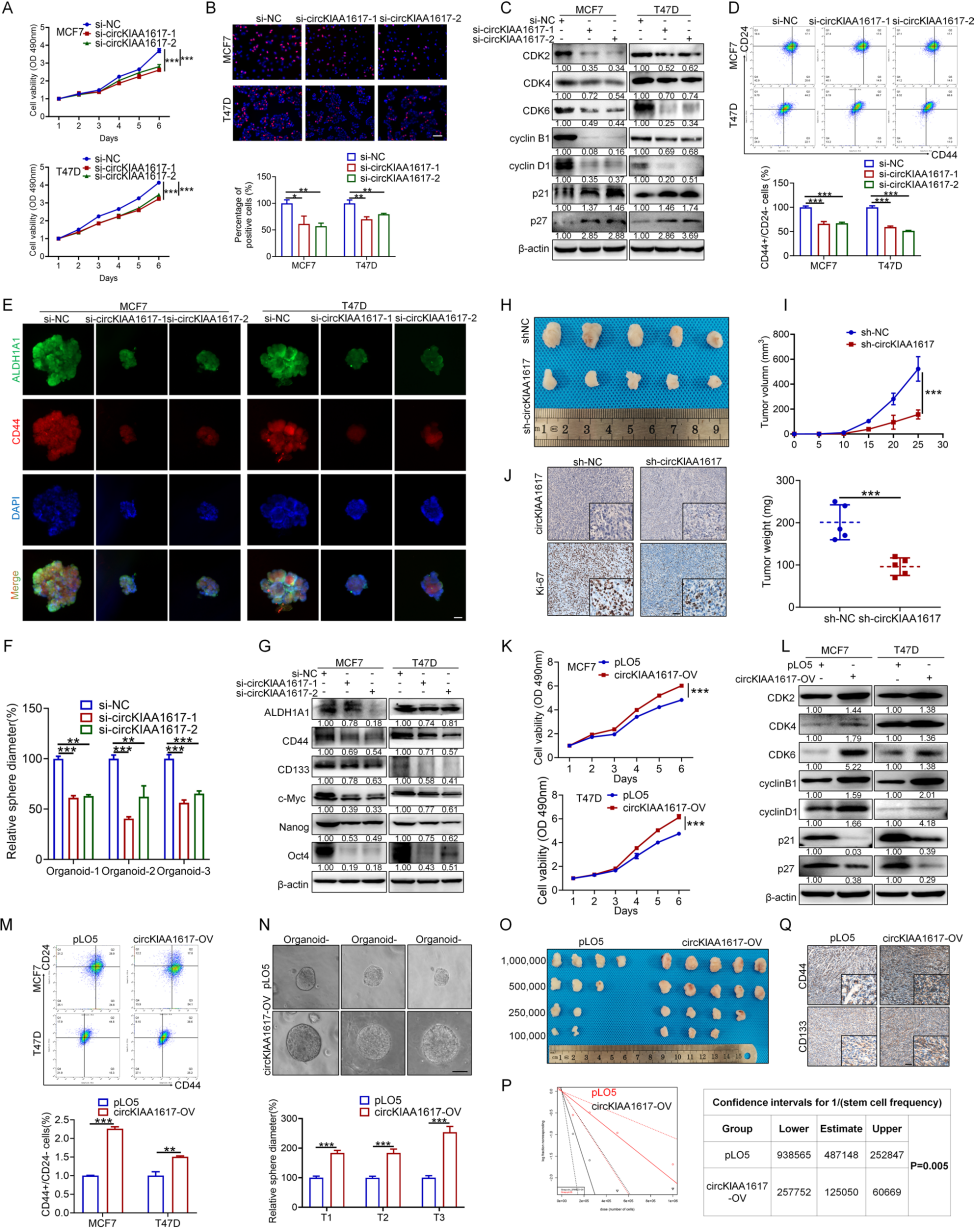
CircKIAA1617 promoted the proliferation and stemness of ER-positive BC cells in vitro and in vivo. MTT (**A**) and EdU (**B**) assays were performed to determine the proliferation rate of the MCF7 and T47D cell lines after transfection with si-circKIAA1617 (*n* = 3). Scale bars = 100 μm. **C.** The proteins associated with the cell cycle were quantified by western blot analysis after circKIAA1617 silencing (*n* = 3). **D** Flow cytometry was used to determine the percentage of MCF7 and T47D cells with the CD44+/CD24- phenotype after circKIAA1617 silencing (*n* = 3). **E** IF staining was performed to evaluate the effect of circKIAA1617 siRNAs on the expression of stemness markers (CD44 and ALDH1A1) in MCF7 and T47D spheres (*n* = 3). Scale bars = 20 μm. **F** Statistical analysis of the PDO diameter after circKIAA1617 silencing (*n* = 3). **G** Western blot assays were performed to assess the expression of stemness-associated proteins. Nude BALB/c mice bearing sustained-release E2 pellets were subcutaneously implanted with MCF7 cells stably transfected with the shRNA targeting circKIAA1617 (*n* = 5 mice/group). The tumor images (**H**), tumor volumes and tumor weights (**I**) were compared between the groups (*n* = 5). **J.** ISH and IHC staining assays were conducted to assess intratumoral circKIAA1617 and Ki-67 expression (*n* = 3). Scale bars = 100 μm. **K** MTT assays were performed to monitor the proliferation rates of the MCF7 and T47D cell lines after transfection with the circKIAA1617 overexpression vector (*n* = 3). **L** Western blot assays were used to quantify the levels of cell cycle-associated proteins after circKIAA1617 overexpression (*n* = 3). **M** Flow cytometry assays were performed to determine the percentage of MCF7 and T47D cells with the CD44+/CD24- phenotype after circKIAA1617 overexpression (*n* = 3). **N** Patient-derived organoid (PDO) models were generated after transfection with circKIAA1617 overexpression vectors (*n* = 3). Scale bars = 100 μm. **O**,** P** In vivo limiting dilution xenograft assays were conducted to evaluate the stemness of circKIAA1617-overexpressing MCF7 cells. **Q** IHC assays were used to detect the expression levels of CD44 and CD133 after circKIAA1617 overexpression (*n* = 3). Scale bars = 100 μm. *P < 0.05; **P < 0.01; and ***P < 0.001

Previous studies have proven that estrogen plays an important role in facilitating the malignant behaviors of ER-positive BC, including proliferation and stemness [16, 17]; thus, we evaluated whether estrogen-induced circKIAA1617 plays regulatory roles in ER-positive BC cells. CircKIAA1617 was initially knocked down with two specific siRNAs, and the position, sequence, and efficiency of the siRNAs are shown in Figure S2. A, B. The mRNA expression levels of its host gene KIAA1617 were also examined using qRT-PCR, which indicated that the outcomes of subsequent experiments did not result from nonspecific knockdown of its host gene (Figure S2. B). MTT and colony formation assays revealed that the knockdown of circKIAA1617 expression significantly decreased the proliferation rate (Fig. 2. A, Figure S2. C). EdU (5-ethynyl-2’-deoxyuridine) and cell cycle assays were subsequently performed and showed that the proliferative activities of MCF7 and T47D cells were impaired following circKIAA1617 silencing (Fig. 2. B, Figure S2. D). Western blot analysis further proved that circKIAA1617 knockdown led to cell cycle arrest in both ER-positive BC cell lines (Fig. 2. C). Given that estrogen can increase stemness to facilitate the progression of ER-positive BC [18], we further assessed the tumor stemness of ER-positive BC cells by performing flow cytometry, tumor sphere formation and immunofluorescence assays and demonstrated that the knockdown of circKIAA1617 markedly attenuated the stemness of ER-positive BC cells (Fig. 2. D, E; Figure S2. E). Previous studies have proven that organoids are in vitro, cell-based models derived from stem cells that possess inherent stemness potential [19]. Therefore, we further verified the association between circKIAA1617 and ER-positive BC stemness using patient-derived organoid (PDO) models, which further exhibited reduced stemness after circKIAA1617 knockdown (Figure S2. F; Fig. 2. F). The levels of proteins related to tumor stemness were also detected, with similar results (Fig. 2. G). Consistent with the in vitro results, we further confirmed that the downregulation of circKIAA1617 suppressed the proliferation and stemness of ER-positive BC cells in vivo (Fig. 2. H-J, Figure S2. G, H).

Given that our findings proved that circKIAA1617 knockdown suppressed the proliferation and stemness of ER-positive BC cells in vitro and in vivo, we further investigated the effects of circKIAA1617 overexpression to comprehensively evaluate its function. A circKIAA1617 overexpression vector (circKIAA1617-OV) was constructed and transfected into MCF7 and T47D cells, and the efficiency was evaluated using qRT-PCR (Figure S3. A). In vitro assays revealed that the overexpression of circKIAA1617 increased the proliferation of MCF7 and T47D cells (Fig. 2. K, L; Figure S3. B-D). In vivo experiments further demonstrated that the overexpression of circKIAA1617 significantly promoted the proliferation of ER-positive BC cells (Figure S3. E-G). Additionally, in vitro assays revealed that the upregulation of circKIAA1617 expression markedly increased the tumor stemness of ER-positive BC cells (Fig. 2. M, Figure S3. H, I). We also overexpressed circKIAA1617 in PDOs derived from ER-positive BC tissues, which further confirmed that circKIAA1617 increased the stemness of ER-positive BC (Fig. 2. N). Western blot analysis of stemness associated markers also proved the stemness-promoting effects of circKIAA1617 (Figure S3. J). Furthermore, rescue experiments were performed and verified that circKIAA1617 did not affect the expression of host gene KIAA1617, and the attenuation of proliferation and stemness after circKIAA1617 knockdown could be reversed by circKIAA1617 overexpression (Figure S3. K-N). We subsequently performed xenograft experiments using limiting dilution assays of MCF7 cells, which indicated that compared with control mice, mice injected with circKIAA1617-overexpressing MCF7 cells exhibited a significantly higher incidence of tumor initiation (Fig. 2. O, P). The expression of the stemness-related proteins CD44 and CD133 was detected using IHC in tumors obtained from mice, and the results were consistent with those described above (Fig. 2. Q). In conclusion, our results showed that circKIAA1617 could increase the proliferation and stemness of ER-positive BC cells both in vitro and in vivo.

### CircKIAA1617 promoted proliferation and stemness by inducing autophagy in ER-positive BC cells

**Fig. 3:**
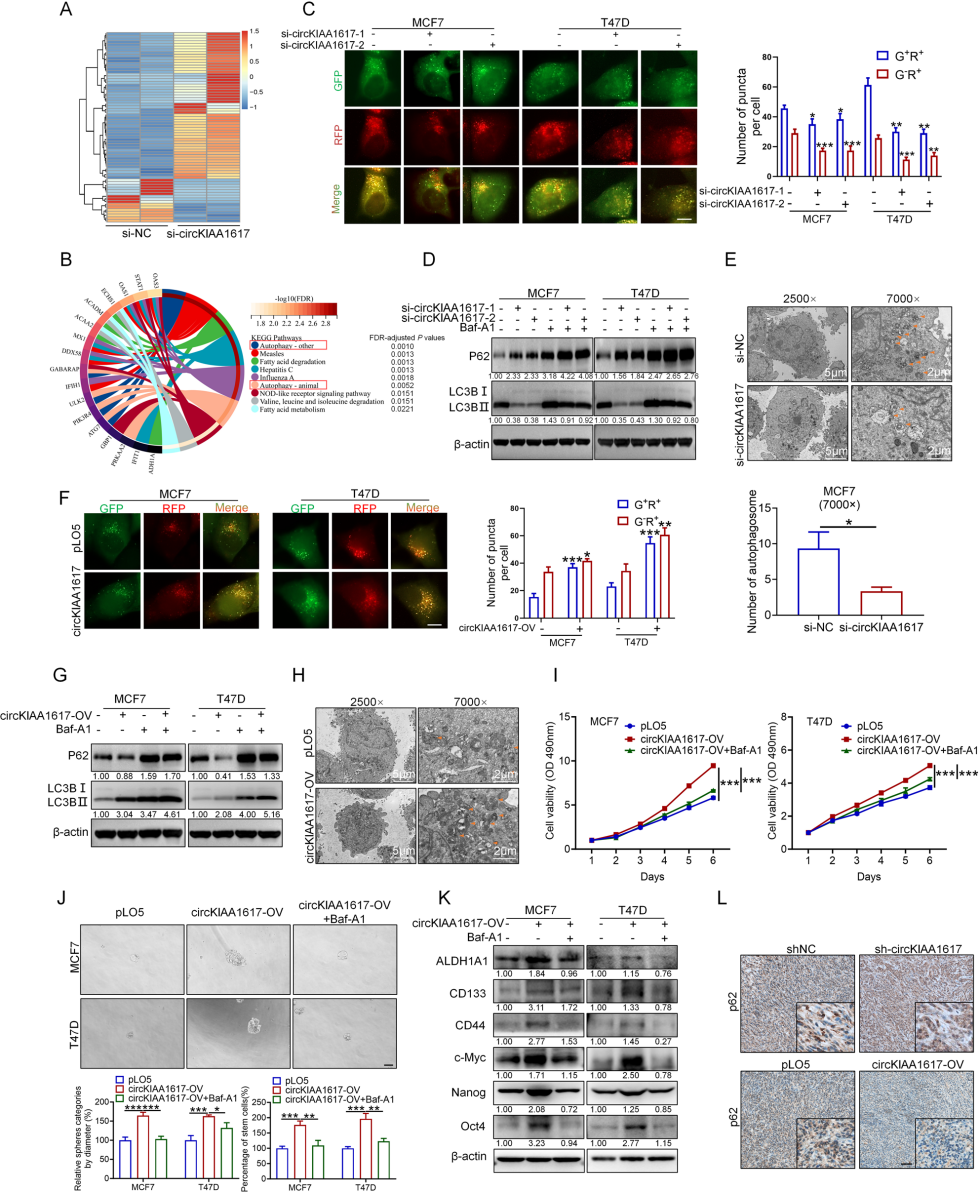
CircKIAA1617 promoted the proliferation and stemness of ER-positive BC cells by inducing autophagy. **(A)** RNA sequencing analysis was performed in circKIAA1617-knockdown ER-positive BC cells. **(B)** KEGG enrichment analysis was used to identify the pathways influenced by circKIAA1617 silencing. **(C)** mCherry-GFP-LC3B was transfected into ER-positive BC cells to assess autophagy flux in MCF7 and T47D cells after circKIAA1617 silencing (*n* = 3). Scale bars = 10 μm. **(D)** Autophagy-related proteins were quantified by western blot analysis after transfection with circKIAA1617 siRNAs (*n* = 3). **(E)** Electron microscopy images of MCF7 cells with circKIAA1617 knockdown (*n* = 3). **(F)** mCherry-GFP-LC3B was transfected into ER-positive BC cells to assess autophagy flux in MCF7 and T47D cells after circKIAA1617 overexpression (*n* = 3). Scale bars = 10 μm. **(G)** Autophagy-related proteins were quantified by western blot analysis after transfection with the circKIAA1617 overexpression vector (*n* = 3). **(H)** Electron microscopy images of MCF7 cells with circKIAA1617 overexpression (*n* = 3). **(I)** MTT assays evaluating the proliferation of ER-positive BC cells after circKIAA1617 overexpression and Baf-A1 (50 nM) treatment (*n* = 3). **(J)** Tumor sphere formation assays examining the effects of Baf-A1 (50 nM) on the circKIAA1617-induced stemness of ER-positive BC cells (*n* = 3). Scale bars = 100 μm. **K.** CircKIAA1617-overexpressing MCF7 and T47D cells were treated with Baf-A1 (50 nM), and the expression of proteins associated with tumor stemness was evaluated (*n* = 3). **L.** IHC assays were performed to detect the expression of the p62 protein in tumors obtained from a xenograft model (*n* = 3). Scale bars = 100 μm. *P < 0.05; **P < 0.01; and ***P < 0.001

Since circKIAA1617 has been shown to be a cancer-promoting gene in ER-positive BC, we further identified the critical downstream pathways correlated with the oncogenic effects of circKIAA1617. RNA-seq was first performed in circKIAA1617 knockdown and control MCF7 cells to identify significantly dysregulated genes (Fig. 3. A), and the Kyoto Encyclopedia of Genes and Genomes (KEGG) enrichment analysis indicated that circKIAA1617 expression was significantly correlated with autophagy in ER-positive BC cells (Fig. 3. B). Monodansylcadaverine (MDC) staining was first performed to verify the correlation between circKIAA1617 expression and autophagy in ER-positive BC cells, which proved that circKIAA1617 knockdown decreased the formation of intracellular autophagosomes (Figure S4. A). Moreover, the mCherry-GFP-LC3B vector was co-transfected with circKIAA1617 siRNAs to evaluate the effects of circKIAA1617 knockdown on autophagy flux in ER-positive BC, and the results revealed that autophagy was significantly inhibited after circKIAA1617 was silenced (Fig. 3. C). Additionally, western blotting further confirmed that circKIAA1617 knockdown attenuated autophagy flux in ER-positive BC (Fig. 3. D). Furthermore, electron microscopy revealed decreased cellular autolysosome formation in circKIAA1617-knockdown cells (Fig. 3. E). In addition, we also examined the effects of circKIAA1617 overexpression on ER-positive BC autophagy. As shown in Fig. 3. F-H and Figure S4. B, C, our results proved that circKIAA1617 overexpression increased the level of autophagy flux in ER-positive BC. Taken together, our results proved that circKIAA1617 increased autophagy in ER-positive BC cells.

Bafilomycin A1 (Baf-A1, an autophagy inhibitor [20]) was used to inhibit circKIAA1617-induced autophagy, and in vitro experiments examining the proliferation and stemness of ER-positive BC cells were performed to verify the regulatory role of circKIAA1617-induced autophagy in the proliferation and stemness of ER-positive BC cells. As shown in Fig. 3. I and Figure S4. D, the suppression of autophagy by Baf-A1 antagonized the acceleration of proliferation induced by circKIAA1617. Moreover, tumor sphere formation and flow cytometry assays further demonstrated that blocking autophagy decreased the stemness of ER-positive BC cells (Fig. 3. J, K and Figure S4. E). In addition, the levels of p62 in xenograft tissues with circKIAA1617 knockdown or overexpression were also evaluated, which further confirmed the positive correlation between circKIAA1617 expression and autophagy in vivo (Fig. 3. L). In conclusion, our results confirmed that circKIAA1617 promoted proliferation and stemness via the induction of autophagy in ER-positive BC.

### CircKIAA1617 was activated by classical Estrogen signaling and cyclized by EIF4A3

**Fig. 4:**
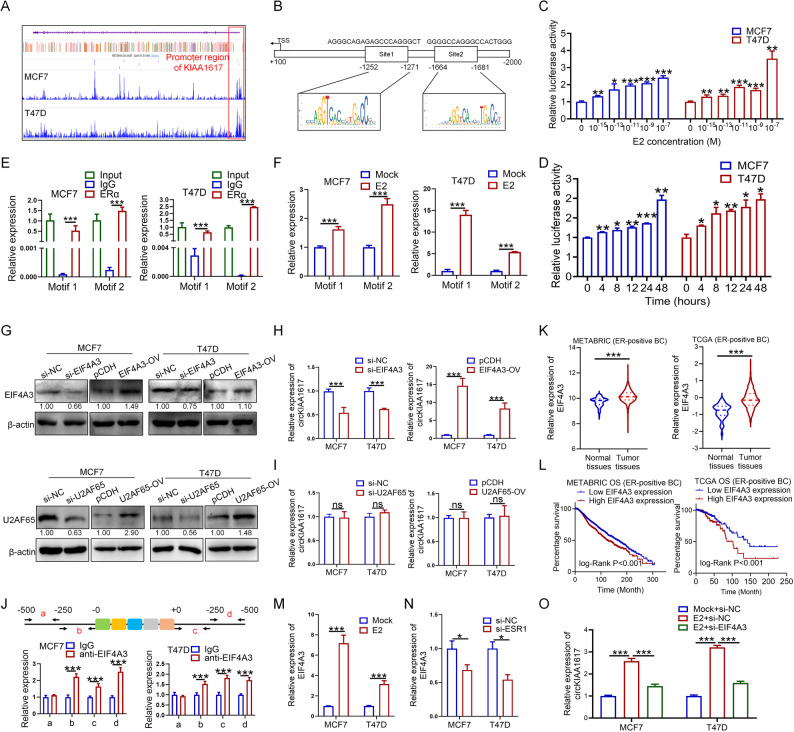
CircKIAA1617 was activated by estrogen and cyclized by EIF4A3 in ER-positive BC cells. **(A)** Genome-wide enrichment profiling of ERα at the promoter region of circKIAA1617 in the MCF7 and T47D cell lines was performed using the Cistrome database. **(B)** The location and sequence of potential binding motifs in the circKIAA1617 promoter region predicted by the JASPAR database. Luciferase activity was detected to assess the transcriptional activity of the wild-type circKIAA1617 promoter in MCF7 and T47D cells after treatment with different concentrations of estrogen (**C**) for different times (**D**) (*n* = 3). **E** ChIP assays were performed to analyze the binding between ERα and the binding motif of the circKIAA1617 promoter, as predicted by the JASPAR database (*n* = 3). **F** ChIP assays evaluating the binding ability between ERα and the motifs in the circKIAA1617 promoter after treatment with estrogen (1 nM) (*n* = 3). **G** EIF4A3 and U2AF65 were silenced or overexpressed in ER-positive BC cells, and the protein levels were determined by western blotting (*n* = 3). qRT-PCR assays were performed to evaluate the effects of EIF4A3 (**H**) and U2AF65 (**I**) on circKIAA1617 expression (*n* = 3). **J** The upstream and downstream flanking sequences of circKIAA1617 were obtained, and primers were designed to verify the binding between EIF4A3 and the circKIAA1617 flanking sequence through RIP assays (*n* = 3). The data from TCGA and METABRIC databases were analyzed, and the expression of EIF4A3 in normal and tumor tissues from patients with ER-positive BC (**K**) and its prognostic significance (**L**) were analyzed using t test and log-rank analyses. The effects of estrogen treatment (**M**) and ESR1 knockdown (**N**) on EIF4A3 expression were evaluated in ER-positive BC cells (*n* = 3). **O** Effects of EIF4A3 knockdown on the estrogen-induced expression of circKIAA1617 (*n* = 3). *P < 0.05; **P < 0.01; and ***P < 0.001

Based on the results described above, we determined that circKIAA1617 is an estrogen-responsive circRNA, and we further evaluated how estrogen regulates circKIAA1617 expression in ER-positive BC. The classical estrogen signaling pathway involves direct binding of ERα to the promoter to activate gene transcription [21]; thus, we hypothesized that circKIAA1617 might be transcriptionally activated by estrogen in ER-positive BC. As shown in Fig. 4. A, ChIP-seq data from Cistrome (http://cistrome.org/) revealed that ERα was enriched at the promoter region of circKIAA1617 in both the MCF7 and T47D cell lines, indicating that ERα has the potential to activate circKIAA1617 transcription. In addition, the sequence of the circKIAA1617 promoter was analyzed, and two binding motifs for ERα were predicted by the JASPAR database (https://jaspar.elixir.no/) (Fig. 4. B). pGL4.26 vectors expressing the wild-type circKIAA1617 promoter were constructed and transfected into ER-positive BC cells to evaluate the effects of estrogen on the transcriptional activity of circKIAA1617 in ER-positive BC cells. Dual-luciferase assays proved that E2 treatment increased the transcriptional activity of circKIAA1617 in a concentration- and time-dependent manner (Fig. 4. C, D), while ESR1 knockdown suppressed the transcriptional activity of circKIAA1617 (Figure S5. A), proving that estrogen could increase the transcriptional activity of the circKIAA1617 promoter. Furthermore, we constructed pGL4.26 vectors expressing the circKIAA1617 promoter with mutant motifs, and the results indicated that both motifs were crucial for the transcription of circKIAA1617 (Figure S5. B). ChIP assays were subsequently performed, which revealed that ERα could bind to both of the predicted motifs (Fig. 4. E). Moreover, the binding of ERα to both motifs could be enhanced by treatment with estrogen (Fig. 4. F). Taken together, these results indicated that estrogen induced the expression of circKIAA1617 via transcriptional activation.

Previous studies have demonstrated that some RNA-binding proteins (RBPs) can bind to circRNA flanking intron sequences and play important roles in the cyclization and biogenesis of circRNAs [13]. Given the well-documented role of ERα as an RNA binding protein [22], we first investigated whether ERα was also involved in this process and played a dual role in circKIAA1617 modulation. As shown in Figure S5. C, RNA Immunoprecipitation (RIP) assays revealed that ERα could not bind to the flanking introns of circKIAA1617. We further explored the underlying mechanism of circKIAA1617 biogenesis in ER-positive BC by predicting the potential RBPs that bind to the flanking regions of circKIAA1617 through a search of the CircInteractome database [23]. As shown in Figure S5. D, EIF4A3 and U2AF65 were suggested to have the potential to bind with the flanking regions of circKIAA1617. Specific siRNAs and overexpression vectors targeting EIF4A3 and U2AF65 were designed and transfected into ER-positive BC cells to verify the effects of EIF4A3 and U2AF65 on the expression of circKIAA1617 (Fig. 4. G, Figure S5. E), which proved that the expression of EIF4A3 but not U2AF65 was positively correlated with the expression of circKIAA1617 (Fig. 4. H, I). RIP assays were also conducted, which confirmed that endogenous EIF4A3 could bind to both upstream and downstream flanking intron sequences of circKIAA1617 (Fig. 4. J), further indicating that EIF4A3 is involved in the cyclization and biogenesis of circKIAA1617. Moreover, a total of four motifs were predicted within the flanking introns of circKIAA1617 and were individually mutated (Figure S5. F, upper panel). RIP assays showed that motif 1, motif 2 and motif 4 were responsible for the binding of EIF4A3 to the flanking introns (Figure S5. F, lower panel). An analysis of the METABRIC and TCGA databases revealed that EIF4A3 was overexpressed in ER-positive BC tissues and correlated with a poor prognosis for ER-positive BC patients, which indicated the potential functions of EIF4A3 in ER-positive BC (Fig. 4. K, L). In addition, we found that the expression of EIF4A3 could be induced by estrogen and inhibited by ESR1 knockdown in ER-positive BC cells (Fig. 4. M, N), indicating the regulatory effects of EIF4A3 on circKIAA1617 expression upon estrogen treatment. Furthermore, EIF4A3 knockdown partly reversed the effects of estrogen on upregulating circKIAA1617 expression in ER-positive BC cells, proving that EIF4A3 was crucial for estrogen-induced circKIAA1617 expression (Fig. 4. O). In conclusion, these results suggested that EIF4A3 could bind to the flanking introns of circKIAA1617 to facilitate its cyclization and biogenesis.

### CircKIAA1617 directly interacted with PGRMC1

**Fig. 5:**
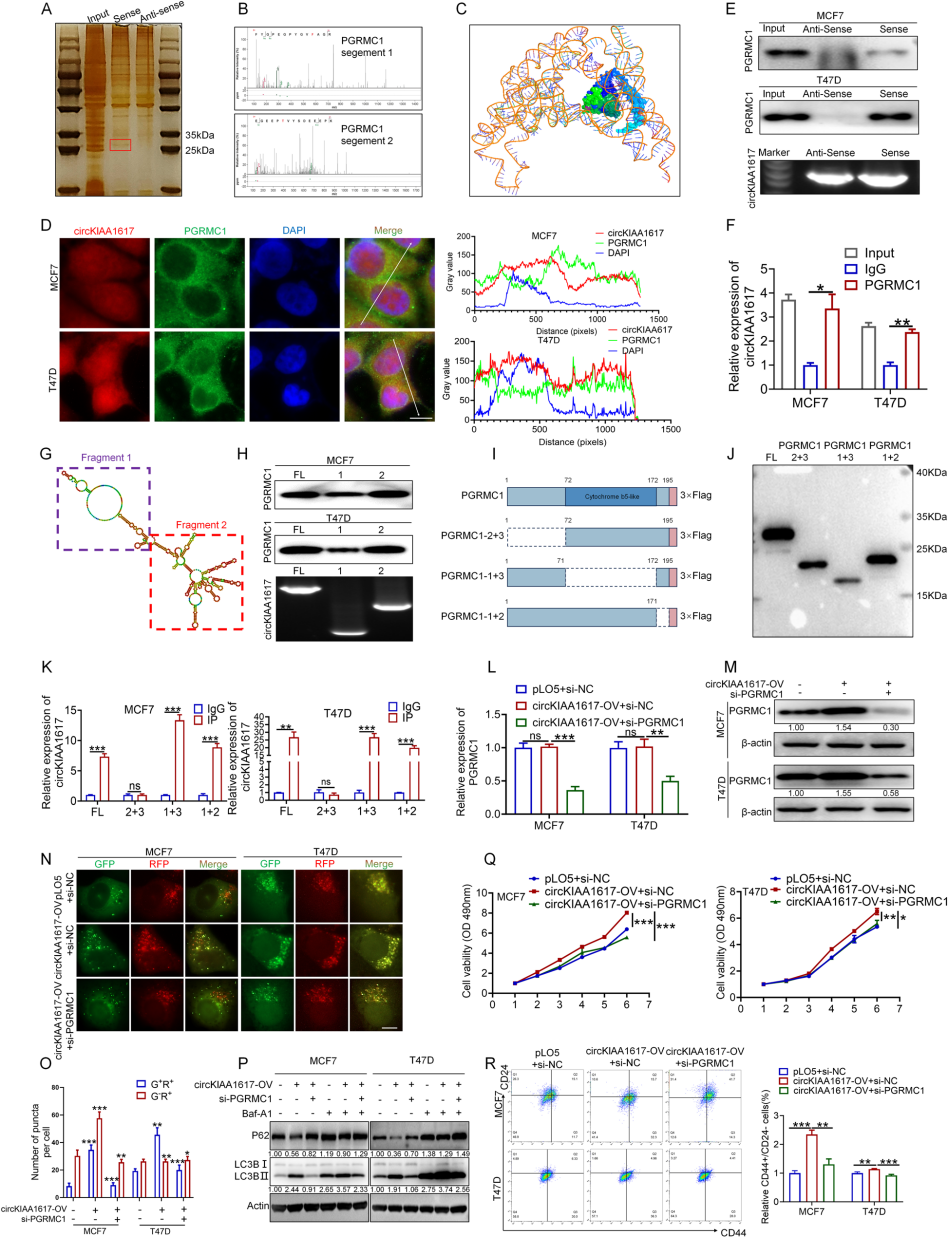
CircKIAA1617 directly interacted with PGRMC1 to promote autophagy, proliferation and stemness in ER-positive BC cells. **(A)** Silver staining was used to identify proteins enriched in RNA pull-down assays using circKIAA1617 (*n* = 3). **(B)** Representative segments of the PGRMC1 protein identified by mass spectrometry (MS). **(C)** Graphical representation of the molecular docking between circKIAA1617 and the PGRMC1 protein by AlphaFold3. **(D)** FISH and IF assays were performed to evaluate the subcellular localization of circKIAA1617 and PGRMC1 (left panel) (*n* = 3). Scale bars = 10 μm. Colocalization of circKIAA1617 and PGRMC1 analyzed using ImageJ (right panel). **(E)** RNA pull-down assays were performed to validate the interaction between circKIAA1617 and PGRMC1 in MCF7 and T47D cells (*n* = 3). Northern blot assays detected biotin-labeled sense and antisense RNAs derived from the reverse transcription of circKIAA1617 (*n* = 3). **(F)** The interaction between circKIAA1617 and PGRMC1 was confirmed by RIP assays in MCF7 and T47D cells (*n* = 3). **(G)** RNAfold was used to predict the secondary structure of circKIAA1617, and circKIAA1617 was divided into two truncations based on the secondary structure. **(H)** RNA pull-down assays were performed to determine the specific truncations responsible for the interaction between circKIAA1617 and PGRMC1 (*n* = 3). Northern blot assays detected full-length and truncated biotin-labeled RNAs derived from the reverse transcription of circKIAA1617 (*n* = 3). **(I)** Schematic diagram of full-length and truncated PGRMC1. **(J)** The expression efficiency of the PGRMC1 full-length and truncation mutant vectors was determined by performing western blot assays. **K** RIP assays were performed to identify the specific domain of PGRMC1 that interacts with circKIAA1617 in MCF7 and T47D cells (*n* = 3). qRT-PCR (**L**) and western blot (**M**) assays were performed to evaluate the effects of the circKIAA1617 overexpression vector and PGRMC1 siRNA on the PGRMC1 mRNA and protein levels (*n* = 3). **N**,** O** mCherry-GFP-LC3B was transfected into ER-positive BC cells to assess autophagy flux in MCF7 and T47D cells after circKIAA1617 overexpression and PGRMC1 knockdown (*n* = 3). Scale bars = 10 μm. **P.** Western blot analysis of the levels of autophagy-associated proteins after circKIAA1617 overexpression and PGRMC1 knockdown (*n* = 3). MTT (**Q**) and flow cytometry (**R**) assays were performed to evaluate the effects of PGRMC1 knockdown on the circKIAA1617-induced proliferation and stemness of ER-positive BC (*n* = 3). ns, not significant; *P < 0.05; **P < 0.01; and ***P < 0.001

We further explored the potential molecular mechanisms by which circKIAA1617 enhances autophagy to promote the proliferation and stemness of ER-positive BC by first performing an RNA pull-down assay to explore potential proteins that bind to circKIAA1617 in MCF7 cells. As shown in Fig. 5. A, a protein band at approximately 25 kDa was markedly enriched with the circKIAA1617 sense sequence compared with the antisense sequence. A liquid chromatography-tandem mass spectrometry (LC-MS) assay was then performed to identify specific proteins of approximately 25 kDa, and 5 potential downstream targets of circKIAA1617 that were significantly enriched in the sense group were identified (Figure S6. A). Among the five proteins, progesterone receptor membrane component 1 (PGRMC1), a protein with a predicted molecular weight of 22 kDa, has been widely reported to participate in autophagy regulation [24, 25], and this PGRMC1 could be the functional target of circKIAA1617 and was selected for further analyses. Previous studies have reported that PGRCM1 can regulate autophagy in various cancers [14, 26], and we hypothesized that PGRCM1 might be the downstream functional target of circKIAA1617. The identified peptide sequences of PGRMC1 are shown in Fig. 5. B. AlphaFold3 (https://alphafoldserver.com/) was further used to predict the molecular docking between circKIAA1617 and the PGRMC1 protein, providing further evidence of the potential interaction between the two molecules (Fig. 5. C).

The subcellular localization of both circKIAA1617 and PGRMC1 was first examined by performing FISH and immunofluorescence (IF) assays to further verify the potential binding between circKIAA1617 and PGRMC1, and the results suggested that circKIAA1617 and PGRMC1 were localized in the cytoplasm of ER-positive BC cells, indicating that circKIAA1617 and PGRMC1 can bind to each other (Fig. 5. D). RNA pull-down assays were subsequently performed in both the MCF7 and T47D cell lines, which proved that PGRMC1 could interact with circKIAA1617 (Fig. 5. E). Moreover, RIP analyses further confirmed that circKIAA1617 was significantly enriched in the immunoprecipitate of PGRMC1 (Fig. 5. F). We generated two truncated isoforms of circKIAA1617, guided by the stem-loop structure predicted using RNAfold (http://rna.tbi.univie.ac.at//cgi-bin/RNAWebSuite/RNAfold.cgi), to identify the specific regions of circKIAA1617 that are essential for the PGRMC1 interaction (Fig. 5. G). RNA pull-down assays were then employed to detect the affinity between the two isoforms and circKIAA1617, and the results showed that both isoforms had the potential to interact with PGRMC1, with the fragment 2 isoform exhibiting a higher binding affinity (Fig. 5. H). Furthermore, three truncated vectors of the PGRMC1 protein were constructed based on the special domain to identify the specific domain of PGRMC1 that is crucial for the circKIAA1617 interaction (Fig. 5. I), and the expression efficiency of the vectors was determined by western blotting (Fig. 5. J). RIP assays were subsequently performed in MCF7 and T47D cell lines transfected with full-length and truncated PGRMC1, confirming that the N-terminal domain spanning residues 1–72 of PGRMC1 constitutes the principal region responsible for mediating its interaction with circKIAA1617 (Fig. 5. K). In conclusion, our results proved that circKIAA1617 could directly interact with the PGRMC1 protein in ER-positive BC cells.

Building upon the established direct interaction between circKIAA1617 and PGRMC1 protein, we investigated whether the expression of PGRMC1 could be influenced by circKIAA1617. Knockdown or overexpression assays showed that circKIAA1617 expression was positively correlated with PGRMC1 protein expression but did not affect its mRNA level (Figure S6. B, C; Fig. 5. L, M), indicating that circKIAA1617 might influence the posttranslational modification of PGRMC1. Moreover, rescue assays were performed in which circKIAA1617 was overexpressed and PGRMC1 was knocked down in ER-positive BC cells to verify whether PGRMC1 is a functional downstream target of circKIAA1617. As shown in Fig. 5. N-P, PGRMC1 knockdown inhibited the induction of autophagy by circKIAA1617. Moreover, in vitro assays further proved that the oncogenic effects of circKIAA1617 on ER-positive BC proliferation and stemness could be inhibited by the PGRMC1 siRNA (Fig. 5. Q, R; Figure S6. D, E). Additionally, PGRMC1 was inhibited by a specific inhibitor (AG-205), further demonstrating that PGRMC1 is functional downstream of circKIAA1617 in ER-positive BC (Figure S6. F-J). In conclusion, the above results confirmed that circKIAA1617 could interact with and modulate the expression of the PGRMC1 protein, a functional target that was responsible for the circKIAA1617-induced autophagy and malignant behaviors of ER-positive BC cells.

### CircKIAA1617 served as a scaffold to enhance the interaction between PGRMC1 and USP14

**Fig. 6:**
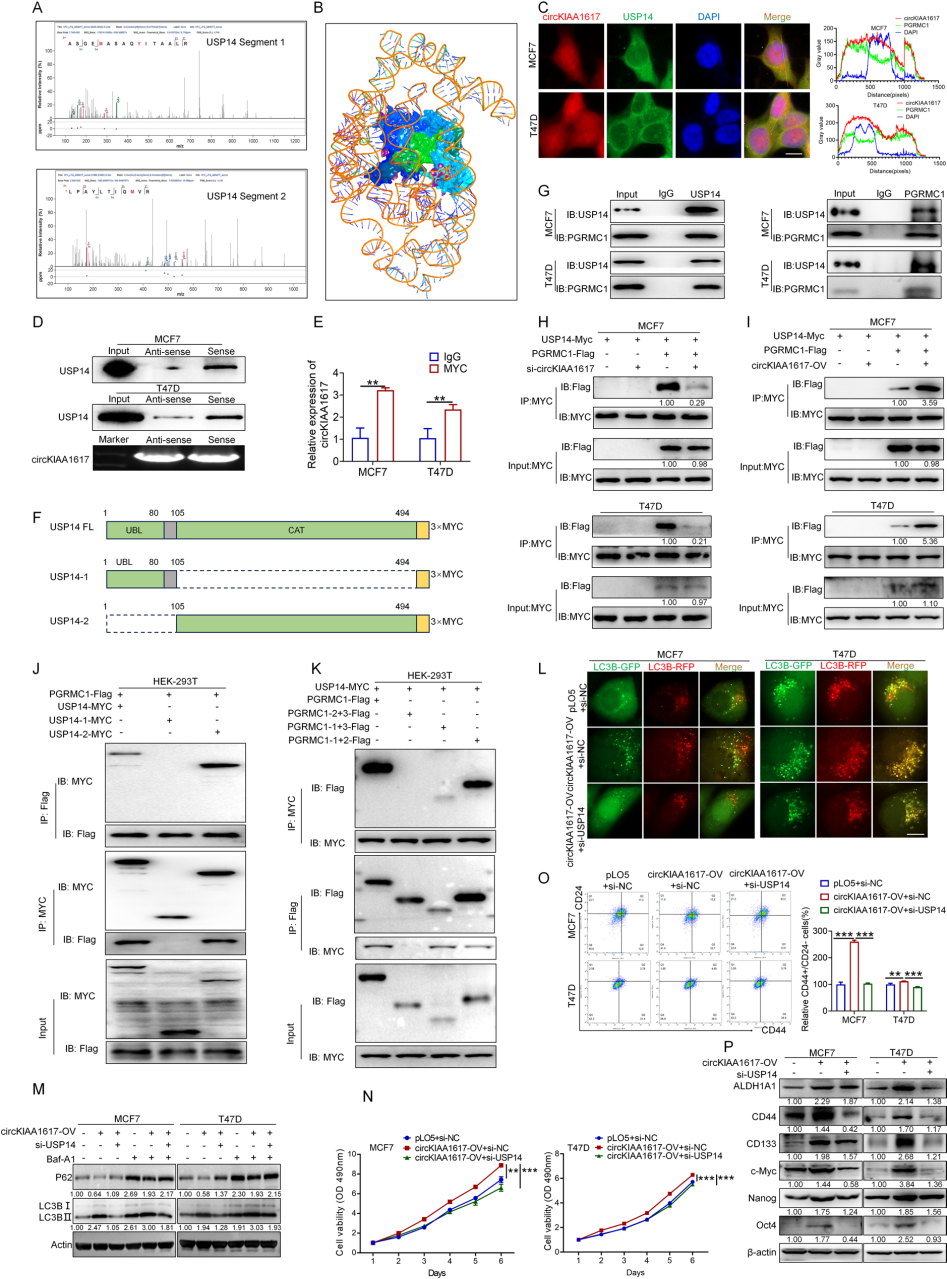
CircKIAA1617 acted as a scaffold to enhance the interaction between USP14 and PGRMC1. **(A)** Representative segments of the USP14 protein identified by mass spectrometry (MS). **(B)** Graphical representation of the molecular docking between the circKIAA1617 protein and USP14 protein by AlphaFold3. **(C)** FISH and IF assays were used to determine the subcellular localization of circKIAA1617 and USP14 (left panel) (*n* = 3). Scale bars = 10 μm. The colocalization of circKIAA1617 and USP14 was analyzed using ImageJ (right panel) (*n* = 3). **(D)** RNA pull-down assays were performed to confirm the interaction between circKIAA1617 and USP14 in MCF7 and T47D cells. Northern blot assays were used to assess biotin-labeled sense and antisense RNAs derived from the reverse transcription of circKIAA1617 (*n* = 3). **(E)** RIP assays were performed to validate the binding between circKIAA1617 and USP14 (*n* = 3). **(F)** Schematic diagram of full-length and truncated USP14. **(G)** Coimmunoprecipitation (Co-IP) and western blot assays were performed to confirm the PGRMC1-USP14 interaction in MCF7 and T47D cells (*n* = 3). **(H)** Interference with circKIAA1617 expression affected the interaction between PGRMC1 and USP14 (*n* = 3). **(I)** The overexpression of circKIAA1617 enhanced the interaction between PGRMC1 and USP14 (*n* = 3). **(J)** Co-IP and western blot assays revealed that the C-terminal catalytic domain of USP14 was required for PGRMC1 binding (*n* = 3). **K** Co-IP and western blot assays demonstrated that the N-terminal domain of PGRMC1 mediated USP14 binding (*n* = 3). **L** mCherry-GFP-LC3B was transfected into ER-positive BC cells to assess autophagy flux in MCF7 and T47D cells after circKIAA1617 overexpression and USP14 knockdown (*n* = 3). Scale bars = 10 μm. **M** Western blot analysis of the levels of autophagy-associated proteins after circKIAA1617 overexpression and USP14 knockdown (*n* = 3). MTT (**N**) and flow cytometry (**O**) assays were performed to evaluate the effects of USP14 knockdown on the circKIAA1617-induced proliferation and stemness of ER-positive BC cells (*n* = 3). **P.** Western blot analysis of the levels of protein markers of tumor stemness after circKIAA1617 overexpression and USP14 knockdown (*n* = 3). **P < 0.01 and ***P < 0.001

Based on the results described, we further evaluated how circKIAA1617 affects the expression of the PGRMC1 protein. Since numerous studies have demonstrated that circRNAs can serve as scaffolds to enhance protein binding and further modulate the stabilization and function of proteins [27], we hypothesized that circKIAA1617 might regulate the interaction between PGRMC1 and its regulatory protein. The RNA pull-down results are shown in Fig. 5. A, and ubiquitin-specific proteinase 14 (USP14), a deubiquitinating enzyme, was selected because of its ability to regulate the ubiquitin-proteasome system [28]. The peptide sequences of USP14 identified by LC-MS are shown, and the molecular docking between circKIAA1617 and the USP14 protein was predicted by AlphaFold3 (Fig. 6. A, B). FISH and IF assays proved that circKIAA1617 and USP14 could colocalize in the cytoplasm of MCF7 and T47D cells (Fig. 6. C). RNA pull-down and RIP assays further showed the interaction between circKIAA1617 and USP14 (Fig. 6. D, E). Moreover, two truncation vectors based on the structural domains of USP14 were constructed (Fig. 6. F, Figure S7. A), and RIP assays were performed to determine the specific domain of USP14 that could bind to circKIAA1617, revealing that the C-terminal domain of USP14 was responsible for the interaction between circKIAA1617 and USP14 in ER-positive BC cells (Figure S7. B).

Since we have proved circKIAA1617 could interact with both the PGRMC1 and USP14 proteins, the binding between PGRMC1 and USP14 was further evaluated. As shown in Fig. 6. G and Figure S7. C, immunoprecipitation (IP) assays were performed with specific antibodies against PGRMC1 and USP14, and the results revealed that PGRMC1 and USP14 could bind to each other. The evidence obtained above suggested that circKIAA1617 might function as a central scaffold to enhance the binding between PGRMC1 and USP14. The effects of circKIAA1617 expression on the binding affinity between PGRMC1 and USP14 were further detected via IP assays to validate our hypothesis, and the results revealed a decreased PGRMC1-USP14 binding affinity following circKIAA1617 knockdown, whereas circKIAA1617 overexpression enhanced this interaction (Fig. 6. H, I). Full-length and truncated USP14 vectors were overexpressed with PGRMC1 to further explore the binding domains between PGRMC1 and USP14, and IP assays revealed that the C-terminal catalytic domain (CAT, 105–494 aa) of USP14 directly binds to PGRMC1 (Fig. 6. J). Moreover, the full-length and truncated PGRMC1 vectors mentioned in Fig. 5 together with the USP14 overexpression vector were transfected into HEK-293T cells, and USP14 specifically bound to the N-terminal domain (1–72 aa) of PGRMC1, as evidenced by IP assays (Fig. 6. K).

USP14 was silenced after circKIAA1617 overexpression in MCF7 and T47D cells to further investigate whether USP14 is a functional downstream target of circKIAA1617 in ER-positive BC cells. As shown in Figure S7. D, E, circKIAA1617 overexpression did not affect the mRNA or protein levels of USP14, whereas USP14 knockdown decreased the PGRMC1 protein level induced by circKIAA1617, indicating that USP14 was crucial for circKIAA1617-regulated PGRMC1 expression. In vitro assays demonstrated that the downregulation of USP14 inhibited the effects of circKIAA1617 on the autophagy, proliferation and stemness of ER-positive BC cells (Fig. 6. L-P, Figure S7. F, G). In conclusion, our results proved that circKIAA1617 served as a scaffold to enhance the interaction between PGRMC1 and USP14, which promoted autophagy, proliferation and stemness in ER-positive BC cells.

### The circKIAA1617/USP14 axis increased the stabilization of PGRMC1 by catalyzing K48-linked deubiquitination at lysine 105

**Fig. 7:**
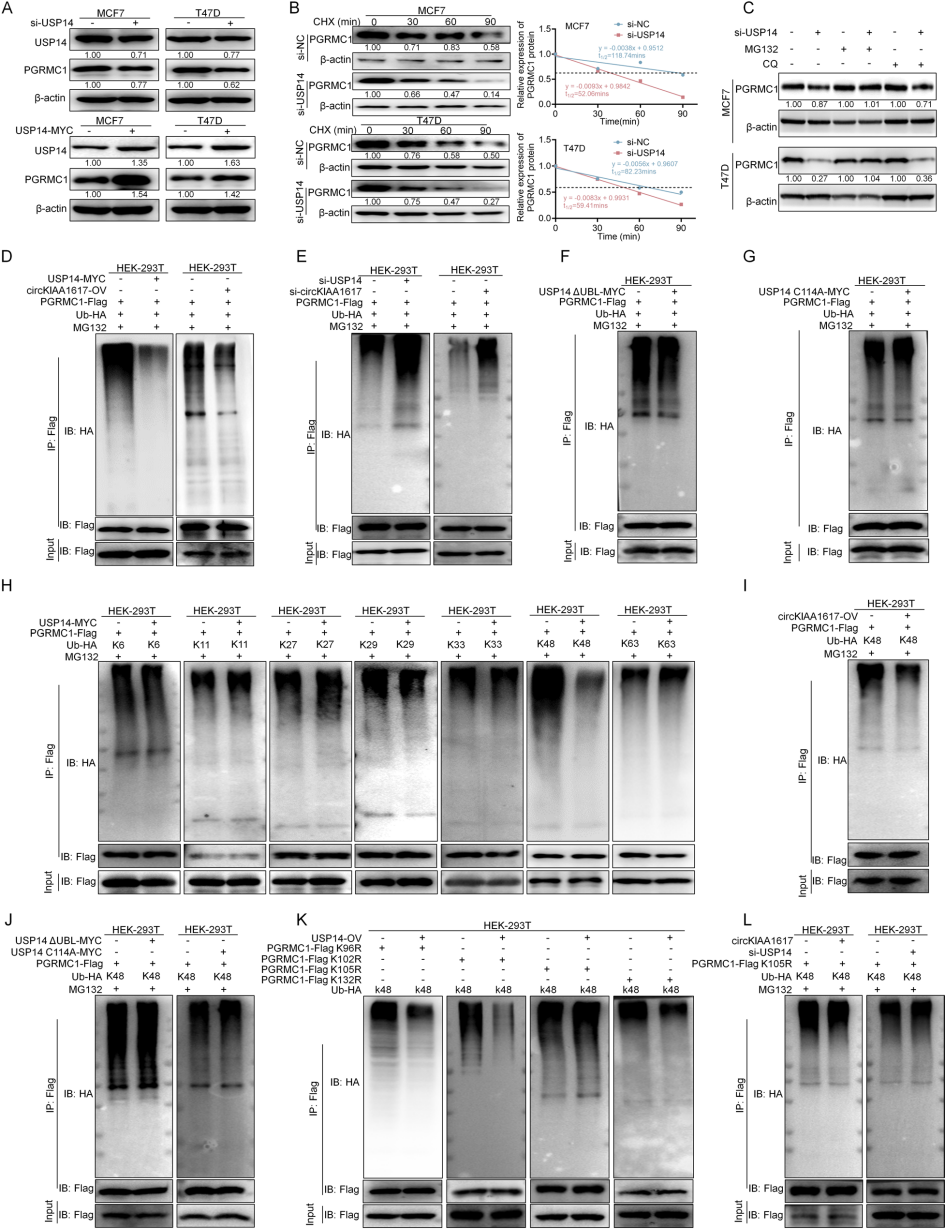
The circKIAA1617/USP14 axis increased the stabilization of the PGRMC1 protein by catalyzing the K48-linked deubiquitination of PGRMC1 on lysine 105. **(A)** USP14 knockdown or overexpression affected PGRMC1 protein expression (*n* = 3). **(B)** MCF7 and T47D cells were transfected with a USP14 siRNA, followed by treatment with 20 µg/ml cycloheximide (CHX) for 0, 30, or 60. 90 min. Immunoblotting and ImageJ software were used to detect the stability of PGRMC1 (*n* = 3). **(C)** ER-positive BC cells were treated with 10 µM MG132 and 10 µM CQ (chloroquine), and USP14 affected the stabilization of the PGRMC1 protein via the proteasomal pathway (*n* = 3). **(D)** Effects of USP14 and circKIAA1617 overexpression on the level of PGRMC1 ubiquitination (*n* = 3). **(E)** Co-IP and western blot assays were performed to determine the effects of USP14 and circKIAA1617 siRNAs on the level of PGRMC1 ubiquitination (*n* = 3). **(F)** Co-IP and western blot assays were performed to evaluate the level of PGRMC1 ubiquitination following USP14-ΔUBL overexpression (*n* = 3). **(G)** The USP14-C114A vector was overexpressed and its effect on the level of PGRMC1 ubiquitination was examined (*n* = 3). **(H)** Co-IP and western blotting revealed that USP14 specifically regulated the K48-linked deubiquitination of PGRMC1 (*n* = 3). **(I)** The effect of circKIAA1617 overexpression on the K48-linked deubiquitination of PGRMC1 was examined (*n* = 3). **(J)** The level of K48-linked ubiquitination of PGRMC1 detected following USP14-ΔUBL and USP14-C114A overexpression (*n* = 3). **K** Identification of a lysine site in the PGRMC1 sequence that was deubiquitinated by USP14 (*n* = 3). **L.** K105 of PGRMC1 was mutated, and the effects of circKIAA1617 and USP14 on the ubiquitination level of mutant PGRMC1 were examined (*n* = 3)

We explored the potential mechanism by which the circKIAA1617/USP14 axis influences PGRMC1 by assessing the PGRMC1 protein and mRNA levels after the knockdown and overexpression of USP14. As shown in Fig. 7. A and Figure S8. A, B, the protein level but not the mRNA level of PGRMC1 was positively correlated with the expression of USP14. Additionally, CHX (cycloheximide) chase assays were performed, which revealed that the knockdown of USP14 decreased the stabilization of the PGRMC1 protein (Fig. 7. B). The proteasome inhibitor MG132 and the lysosome inhibitor CQ (chloroquine) were used to verify whether USP14 influenced the stabilization of PGRMC1 via the ubiquitin-proteasome system or lysosome, and the results revealed that inhibition of the ubiquitin-proteasome pathway blocked the effect of USP14 on PGRMC1 protein expression (Fig. 7. C).

Based on these results, the effects of USP14 and circKIAA1617 on the level of PGRMC1 ubiquitination were evaluated, and we found that both USP14 and circKIAA1617 decreased the ubiquitination level of PGRMC1 (Fig. 7. D, E). Moreover, USP14 overexpression vectors with a deletion of the UBL domain (ΔUBL) or carrying a C114A inactive mutation [29] were constructed (Figure S8. C), and the results showed that the overexpression of the ΔUBL or C114A mutant vector failed to decrease the level of PGRMC1 ubiquitination (Fig. 7. F, G), further confirming the regulatory effect of USP14 on the PGRMC1 protein. Moreover, our results demonstrated that USP14 and circKIAA1617 selectively catalyzed the removal of K48-linked polyubiquitin chains from PGRMC1 but exhibited no activity toward K6, K11, K27, K29, K33 or K63-linked chains (Fig. 7. H, I). In addition, the overexpression of USP14 mutant vectors or the K48-resistant (K48R) form of ubiquitin further proved that USP14 and circKIAA1617 regulated the K48-linked polyubiquitination of the PGRMC1 protein (Figs. 7. J and S8. D).

PGRMC1 was partitioned into three regions (residues 1–71, 72–171, and 172–195), and all lysine residues in each region were mutated (K1R, K2R, K3R) to identify the specific lysine site in the PGRMC1 protein that is regulated by the circKIAA1617/USP14 axis, and we found that USP14 affects mainly the K48-linked polyubiquitination of the cytochrome b5-like domain of PGRMC1 (72–171 aa) (Figure S8. E). Furthermore, the PGRMC1 protein sequence was predicted using GPS-UBER [30] (Figure S8. F), and four high-confidence lysine ubiquitination sites (K96, K102, K105 and K132) clustered within the cytochrome b5-like domain were predicted to be possibly regulated by USP14. Vectors containing mutations of the four lysine sites were constructed (K-R) and cotransfected with the USP14 overexpression vector; the results revealed that lysine 105 of PGRMC1 was the specific site that was deubiquitinated by USP14 (Figure S8. G; Fig. 7. K). Moreover, the ubiquitination levels of the mutant PGRMC1 protein (K105R) were further examined after the overexpression of circKIAA1617 or silencing of circKIAA1617 and USP14, further proving that lysine 105 of the PGRMC1 protein was regulated by the circKIAA1617/USP14 axis (Figure S8. H; Fig. 7. L). In addition, a K48-Ub-specific antibody was used, confirming that USP14 modulated the K48-linked ubiquitination of PGRMC1 at lysine 105 (Figure S8. I). A conservation analysis revealed that lysine 105 was conserved across species, indicating the potential functional role of lysine 105 in the PGRMC1 protein (Figure S8. J). Furthermore, we generated two siRNA-resistant PGRMC1 constructs encoding wild-type PGRMC1 (PGRMC1_res_-Flag WT) or the K105R mutant (PGRMC1_res_-Flag K105R) by synonymous mutation (Figure S8. K). The constructs were transfected into cells with endogenous PGRMC1 knockdown, and the effects of circKIAA1617 silencing on PGRMC1res-Flag WT/PGRMC1res-Flag K105R mediated proliferation and stemness were examined. In vitro assays demonstrated that the circKIAA1617 phenotype depended on PGRMC1 stabilization via the deubiquitination of K105 (Figure S8. L-N). Taken together, our results demonstrated that the circKIAA1617/USP14 axis increased the stabilization of the PGRMC1 protein by catalyzing its K48-linked deubiquitination at lysine 105.

### CircKIAA1617-induced autophagy modulated lipophagy to facilitate proliferation and stemness

**Fig. 8:**
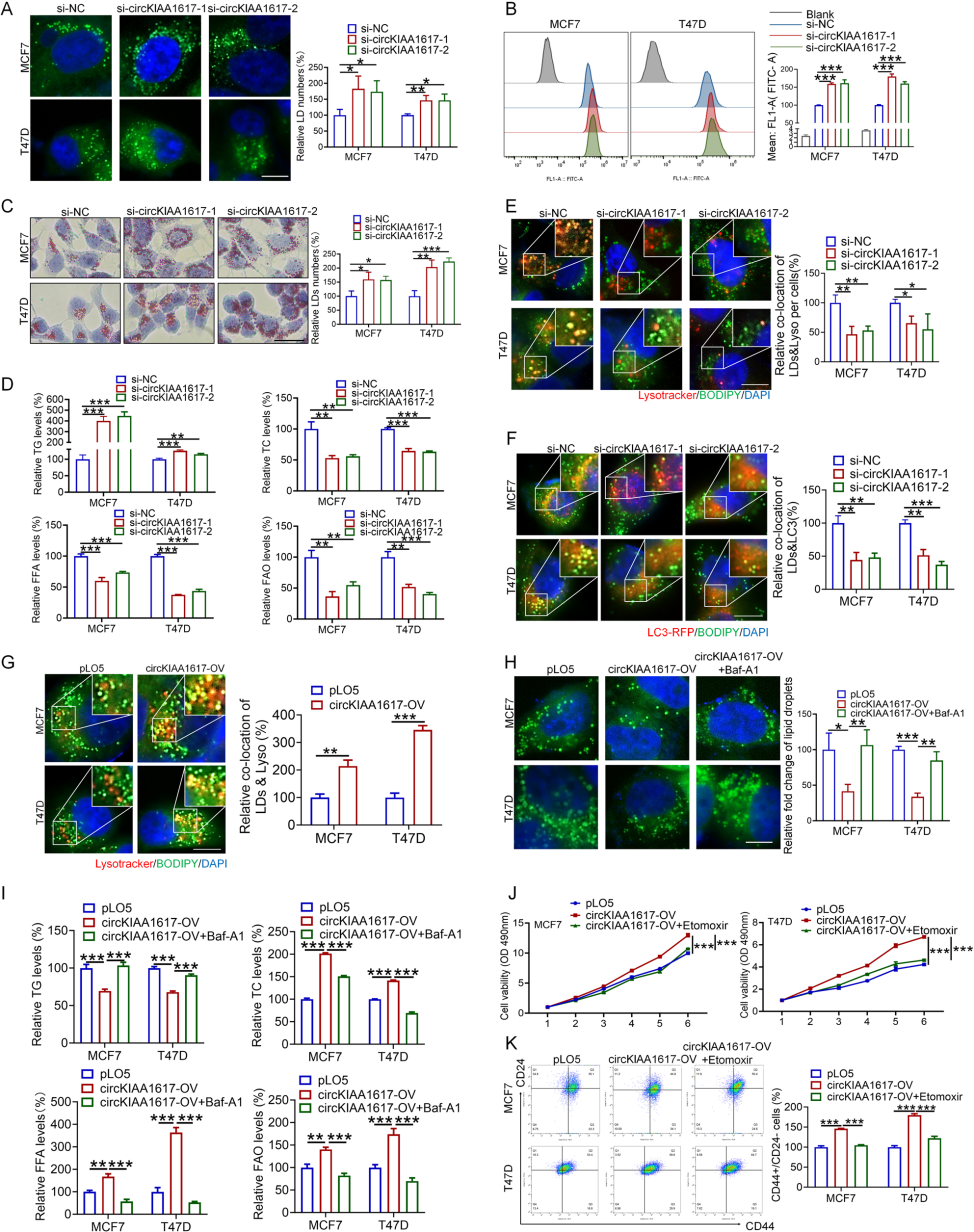
CircKIAA1617-induced autophagy modulated lipophagy to facilitate the proliferation and stemness of ER-positive BC cells. **A** BODIPY staining was used to determine the number of LDs in MCF7 and T47D cells after circKIAA1617 knockdown (*n* = 3). Scale bars = 10 μm. **B** LDs were labeled with BODIPY, and the number of intracellular LDs in MCF7 and T47D cells transfected with si-circKIAA1617 was assessed using flow cytometry (*n* = 3). **C** Oil red O staining was performed to determine the number of LDs in MCF7 and T47D cells after circKIAA1617 knockdown (*n* = 3). Scale bars = 50 μm. **D** TG, TC, FFA and FAO levels were measured after transfection with si-circKIAA1617 (*n* = 3). **E** BODIPY and LysoTracker staining were performed to determine the colocalization of LDs and lysosomes after circKIAA1617 knockdown (*n* = 3). Scale bars = 10 μm. **F** The colocalization of LC3 and LDs was examined after circKIAA1617 silencing in MCF7 and T47D cells (*n* = 3). Scale bars = 10 μm. **G** Colocalization of LDs and lysosomes was assessed in MCF7 and T47D cells overexpressing circKIAA1617 (*n* = 3). Scale bars = 10 μm. **(H)** CircKIAA1617-overexpressing ER-positive BC cells were treated with Baf-A1 (50 nM), and the number of intracellular LDs was determined (*n* = 3). **(I)** CircKIAA1617-overexpressing ER-positive BC cells were treated with Baf-A1 (50 nM), and the levels of TG, TC, FFAs and FAO were measured (*n* = 3). CircKIAA1617-overexpressing ER-positive BC cells were treated with Baf-A1 (50 nM), and the proliferation and stemness of ER-positive BC cells were evaluated by performing MTT (**J**) and tumor sphere formation (**K**) assays (*n* = 3). *P < 0.05; **P < 0.01; and ***P < 0.001

Since we have proven that circKIAA1617 promoted the proliferation and stemness of ER-positive BC by inducing autophagy, we further evaluated how circKIAA1617/USP14/PGRMC1-induced autophagy facilitated the malignant behaviors of ER-positive BC. As shown in Fig. 3. B, in addition to autophagy, we found that the genes whose expression was dysregulated after circKIAA1617 knockdown were also enriched in pathways associated with fatty acid degradation and fatty acid metabolism, indicating that circKIAA1617 might also play crucial roles in regulating lipid metabolism in ER-positive BC. The number of lipid droplets (LDs), which store free fatty acids (FFAs) and total cholesterol (TC) as triglycerides (TGs) in cells, was first determined by staining with BODIPY 493/503 or Oil red O in ER-positive BC cells to verify our hypothesis. As shown in Fig. 8. A-C, the number of LDs in ER-positive BC cells increased significantly after circKIAA1617 was silenced. Moreover, circKIAA1617 knockdown increased the level of TGs but decreased the levels of TC and FFAs, indicating that circKIAA1617 might promote the degradation of LDs (Fig. 8. D). Furthermore, the detection of fatty acid oxidation and carnitine palmitoyl transferase 1 A (CPT1A) levels in ER-positive BC cells also proved that circKIAA1617 knockdown blocked FAO in ER-positive BC cells (Fig. 8. D, Figure S9. A). Since we demonstrated the correlations between circKIAA1617, autophagy and lipid metabolism, we further evaluated whether lipophagy, a novel type of autophagy that selectively degrades LDs into FFAs and cholesterol through the lysosome-dependent lipolytic pathway, accounted for these results. As shown in Fig. 8. E, BODIPY was used to stain LDs, and LysoTracker was used to stain lysosomes; circKIAA1617 knockdown inhibited the colocalization of LDs and lysosomes. Furthermore, circKIAA1617 silencing suppressed the colocalization of LDs and LC3, indicating that circKIAA1617 knockdown inhibited the degradation of LDs by lipophagy (Fig. 8. F). Moreover, the effects of circKIAA1617 overexpression on the levels of lipophagy and fatty acid oxidation (FAO) were evaluated, which further confirmed that circKIAA1617 enhanced lipophagy and FAO in ER-positive BC cells (Fig. 8. G, Figure S9. B-G). Additionally, the knockdown of adipose triacylglyceride lipase (ATGL) further proved the effects of circKIAA1617 on lipophagy (Figure S9. H, I). Furthermore, circKIAA1617-overexpressing cells were treated with Baf-A1 to verify whether circKIAA1617-induced autophagy was responsible for lipophagy in ER-positive BC cells, and the results proved that the inhibition of autophagy suppressed LD degradation in ER-positive BC cells (Fig. 8. H, I).

Since we demonstrated that circKIAA1617-induced autophagy enhanced lipophagy and subsequent FAO in ER-positive BC cells, we explored whether lipophagy-induced FAO was required for the proliferation and stemness of ER-positive BC cells. As shown in Figure S9. J, etomoxir was used to inhibit circKIAA1617-induced FAO. The proliferation and stemness of ER-positive BC cells were evaluated, and the results confirmed that the attenuation of FAO could suppress the oncogenic effects of circKIAA1617 (Fig. 8. J, K, Figure S9. K-N), indicating that lipophagy-mediated fatty acid metabolism is necessary for the circKIAA1617-driven phenotype. In conclusion, our results proved that circKIAA1617-induced autophagy enhanced lipophagy and fatty acid oxidation to facilitate the proliferation and stemness of ER-positive BC cells.

## Discussion

Estrogen receptor-positive breast cancer consists of the luminal A and luminal B molecular subtypes, accounting for approximately 70–80% of all newly diagnosed breast cancers globally, which makes it the most prevalent and clinically heterogeneous form of the disease [1]. Estrogen overexposure and aberrant activation of the ER signaling pathway are thought to be the main drivers of ER-positive BC [31], influencing key malignant biological behaviors such as proliferation and stemness in ER-positive BC [32]. For instance, Kumar et al. reported that the stabilization of DLL1 by estrogen signaling mediated Notch signaling in breast cancer to promote tumor cell progression [33]. In another study, researchers reported that estrogen promoted cancer stemness in ER-positive BC cells via Gli1 [18]. Accumulating evidence indicates that estrogen regulates tumor progression through effects on noncoding RNAs, notably circRNAs. For example, estrogen-regulated circPGR drives ER-positive breast cancer progression by acting as a competing endogenous RNA to sponge miR-301a-5p and regulate the expression of multiple cell cycle genes [10]. In addition, estrogen increases circFAM171A1 levels in myoblasts, which in turn modulates the oar-miR-485-5p/MAPK15 axis to increase myoblast proliferation and limit apoptosis [34]. However, the regulatory roles and mechanisms of estrogen-induced circRNAs have not been fully elucidated and need further investigation.

In this study, using circRNA-seq screening, bioinformatics analyses and qRT-PCR validation, we identified circKIAA1617 as an estrogen-responsive circRNA whose expression is upregulated in ER-positive BC cells and tissues. In vitro and in vivo studies verified that circKIAA1617 knockdown could inhibit the proliferation and stemness of ER-positive BC cells and that circKIAA1617 overexpression had oncogenic effects, which confirmed the vital role of circKIAA1617 in ER-positive BC. Moreover, circKIAA1617 expression is correlated with the clinicopathological characteristics and poor prognosis of patients with ER-positive BC. Notably, considering that patients with ER-positive breast cancer routinely receive adjuvant endocrine therapy, the association between high circKIAA1617 expression and shorter overall survival suggests that elevated circKIAA1617 levels might indicate potential endocrine resistance. Further functional assays and multicenter validation should be performed to verify the roles and predictive values of circKIAA1617 in endocrine resistance of ER-positive BC. RNA-seq was subsequently performed in MCF7 cells with circKIAA1617 knockdown to explore the downstream pathway regulated by circKIAA1617 and responsible for the oncogenic effects of circKIAA1617. The enrichment analysis indicated that the expression of circKIAA1617 was closely associated with the progression of autophagy in ER-positive BC cells. As a cellular process involved in the degradation and recycling of cellular components, autophagy has been implicated in multiple aspects of cancer development [35]. Previous studies have shown the oncogenic roles of autophagy in various cancers, including the survival, proliferation and stemness of cancer cells. For instance, Kao et al. reported that TFEB- and TFE3-dependent autophagy activation supported cancer cell proliferation in the absence of centrosomes [36]; hypoxia-induced galectin-8 expression maintained stemness in glioma stem cells by regulating autophagy [37]. In the present study, our in vitro and in vivo results confirmed the effect of circKIAA1617 on driving the autophagy of ER-positive BC, and the inhibition of autophagy by Baf-A1 reversed the oncogenic effects of circKIAA1617, indicating that circKIAA1617 promoted ER-positive BC proliferation and stemness via the induction of autophagy.

Given that we have proven that circKIAA1617 is an estrogen-responsive circRNA, we further evaluated the mechanisms underlying estrogen-induced circKIAA1617 biogenesis in ER-positive BC. The binding of estrogen to ERα can recruit coactivators to initiate the transcription of oncogenes [38], further promoting the progression of ER-positive BC. For example, estrogen increases the transcription of ERINA to facilitate the progression of breast cancer [39], and the estrogen-inducible kinase SGK3 promotes the survival of breast cancer cells [40]. We thus hypothesized that circKIAA1617 might be an oncogene that is also transcriptionally activated by estrogen in ER-positive BC, which was further confirmed in our ChIP and luciferase assays. The back-splicing of circRNAs is catalyzed by the canonical spliceosomal machinery and modulated by both intronic complementary sequences (ICSs) and RNA binding proteins (RBPs) [7]; thus, we further explored potential RBPs responsible for the biogenesis of circKIAA1617. EIF4A3 is the core component of the exon junction complex (EJC), which is considered an important regulator of posttranscriptional regulatory processes, including mRNA splicing, transport, translation, and surveillance [41], and has been reported to regulate the biogenesis of oncogenic circRNAs such as circSIPA1L3 and circSEPT9 to facilitate the progression of cancers [42, 43]. Through bioinformatics analysis and experimental verification, we found that EIF4A3 could bind to both the upstream and downstream flanking sequences of circKIAA1617 pre-mRNA and was positively correlated with the expression of circKIAA1617, which proved that EIF4A3 could regulate the biogenesis and cyclization of circKIAA1617. Given that circKIAA1617 is identified as a direct transcriptional target of ERα, the circKIAA1617-mediated oncogenic axis should be broadly applicable across ER-positive breast cancer subtypes, including both Luminal A and Luminal B tumors. It was important to note that the intensity of ER signaling and the landscape of transcriptional co-regulators could vary significantly between subtypes [44], and differences in ER signaling strength might influence circKIAA1617 regulation.

Recently, the ability of circRNAs to bind with proteins has attracted increasing attention, as circRNAs can act as scaffolds that promote the binding of different proteins, further regulating the expression, functions and subcellular locations of downstream proteins [45, 46]. We explored the molecular mechanism by which circKIAA1617 enhances autophagy to promote proliferation and stemness in ER-positive BC by performing RNA pull-down and LC-MS to identify downstream targets of circKIAA1617, which revealed that circKIAA1617 could directly bind to the PGRMC1 protein. PGRMC1 has emerged as a heme-binding protein that mechanistically underpins various cellular and tissue functions, including cytochrome P450 activity, heme homeostasis, protein quality control, female reproduction, and cancer [47]. Previous studies have indicated that PGRMC1 converges as a pancancer modulator that promotes proliferation, stemness and chemoresistance in malignant tumors and that its expression is correlated with shorter overall survival of individuals with cancers [48, 49]. For instance, Zhao et al. reported that PGRMC1 potentiated cell proliferation through the suppression of ferroptosis in triple-negative breast cancer [50], and Guan et al. found that cancer stem cell phenotypes and chemotherapy resistance are increased by PGRMC1 through the modulation of AHR ubiquitination in non-small cell lung cancer [51]. Moreover, studies have indicated that PGRMC1 plays regulatory roles in cancer autophagy. For example, Shakeel et al. reported that PGRMC1 is required for the degradative activity of autophagy [24] and that PGRMC1-mediated autophagy increases the resistance of glioblastoma to radiotherapy [14]. We identified PGRMC1 as a direct functional target of circKIAA1617, which modulates PGRMC1 protein levels without altering its mRNA expression. Notably, PGRMC1 knockdown abolished circKIAA1617-mediated autophagy, proliferation, and stemness, validating the circKIAA1617/PGRMC1 oncogenic axis. We elucidated the potential molecular mechanism by which circKIAA1617 regulates PGRMC1 expression by further analyzing the results of RNA pull-down assays, and USP14 was also shown to be enriched by circKIAA1617 probes. We hypothesized that circKIAA1617 might act as a scaffold to enhance the interaction between PGRCM1 and USP14, as demonstrated in our study. USP14 is a member of the ubiquitin-specific protease (USP) family, which is the largest family of deubiquitylating enzymes and orchestrates various cellular processes through the removal of ubiquitins from target proteins to modulate the stabilization, localization and function of proteins [52, 53]. USP14 plays a critical role in the posttranslational regulation of proteins related to tumorigenesis and development. For instance, Shi et al. reported that USP14 deubiquitinates and upregulates IDO1, enhancing immune suppression to promote colorectal cancer progression [28]; Li et al. revealed a mechanism underlying USP14-mediated stabilization of SDC2 in gastric cancer tissue, which promotes the growth and invasive capability of gastric cancer cells [54]. In the present study, our results proved that the knockdown or overexpression of USP14 could affect the expression and stabilization of PGRMC1, which was mediated by the ubiquitin-proteasome pathway, indicating that circKIAA1617 increased PGRMC1 protein expression via USP14-mediated deubiquitination. Ubiquitination is among the most common and important posttranslational modifications of proteins, and different types of ubiquitination mediate distinct fates of substrate proteins [55]. For instance, K48-linked polyubiquitination usually leads to the proteasome-dependent degradation of proteins, whereas K63-linked polyubiquitination is implicated in signal transduction [55]. In our study, we demonstrated that USP14 increased the stabilization of the PGRMC1 protein by catalyzing the K48-linked deubiquitination of PGRMC1 at lysine 105, which accounted for the role of circKIAA1617 in regulating autophagy in ER-positive BC cells.

Autophagy is a complex process that can be divided into several distinct types depending on the specific cellular components targeted for degradation, including mitophagy, lipophagy, and ER-phagy, each of which plays crucial roles in maintaining cellular homeostasis and cancer progression [56]. In this study, we further explored which aspect of cellular progression was influenced by circKIAA1617/USP14/PGRMC1-mediated autophagy to facilitate the proliferation and stemness of ER-positive BC. Based on the KEGG enrichment results from circKIAA1617 knockdown cells, we noticed that pathways correlated with lipid metabolism reprogramming were also significantly dysregulated in circKIAA1617-silenced cells. Notably, lipid metabolic reprogramming refers to adaptive changes in lipid synthesis, uptake, storage, and breakdown in cells, and it plays crucial roles in the regulation of tumor cell fate [57]. The detection of LDs, TG, TC, FFAs and FAO indicated that circKIAA1617-induced autophagy might be associated with the degradation of LDs and increased fatty acid metabolism. Recent studies have shown that autophagy intersects with lipid metabolism in tumors by driving lipid droplet catabolism and fatty acid release, playing a pivotal role in orchestrating the metabolic mechanisms underlying tumor progression [58, 59]. Lipophagy, an autophagy-lysosome-dependent process that mobilizes intracellular lipid droplets into cholesterol and FFAs for β-oxidation, plays critical roles in the pathogenesis and progression of various diseases [60–63]. In tumor cells, lipophagy is a dynamically regulated process governed by complicated molecular networks to alter the malignant biological behavior of cancers. For example, lipophagy facilitates the generation of FFAs to promote senescence in prostate cancer [64], and lipophagy mediated by the AURKA/DDX5/TMEM147-AS1/let-7 signaling axis promotes cisplatin resistance in epithelial ovarian cancer [65]. Moreover, PGRMC1, the downstream functional target of circKIAA1617, has been reported to be associated with lipophagy in cancer cells [66]. In our study, in vitro and in vivo experiments were performed, and we confirmed that circKIAA1617-induced autophagy enhanced lipophagy to facilitate the proliferation and stemness of ER-positive BC cells. Moreover, etomoxir was used to specific inhibite carnitine palmitoyltransferase 1 (CPT1), which serves as the rate-limiting enzyme for mitochondrial β-oxidation [67], further proving that circKIAA1617 associated lipophagy facilitated proliferation and stemness of ER-positive BC cells via enhancing FAO. Crucially, this process established a hierarchical axis where lipophagy-dependent mobilization of lipid droplets provided essential substrates to fuel fatty acid oxidation, thereby positioning FAO as a downstream metabolic effector that sustained the energetic demands of tumor proliferation and stemness.

Our findings highlighted a mechanistic paradigm that was distinct from the majority of previously reported autophagy-regulating circRNAs [68–70]. Firstly, circKIAA1617 was an estrogen responsive circRNA that facilitated autophagy in ER-positive BC, which could partly explicate the autophagy-promoting roles of estrogen reported in tumors [71, 72]. Moreover, our study demonstrated that circKIAA1617 enhanced autophagy via USP14/PGRMC1 axis, which specifically targeted LDs and promoted lipid metabolism reprogramming. It is important to note that our study establishes the necessity of the FAO pathway, as its inhibition effectively reversed circKIAA1617-induced malignant phenotypes. However, whether lipophagy activation is sufficient on its own to drive these behaviors warrants further investigation, and other circKIAA1617-mediated mechanisms may also contribute to tumor progression.

## Conclusions

**Fig. 9:**
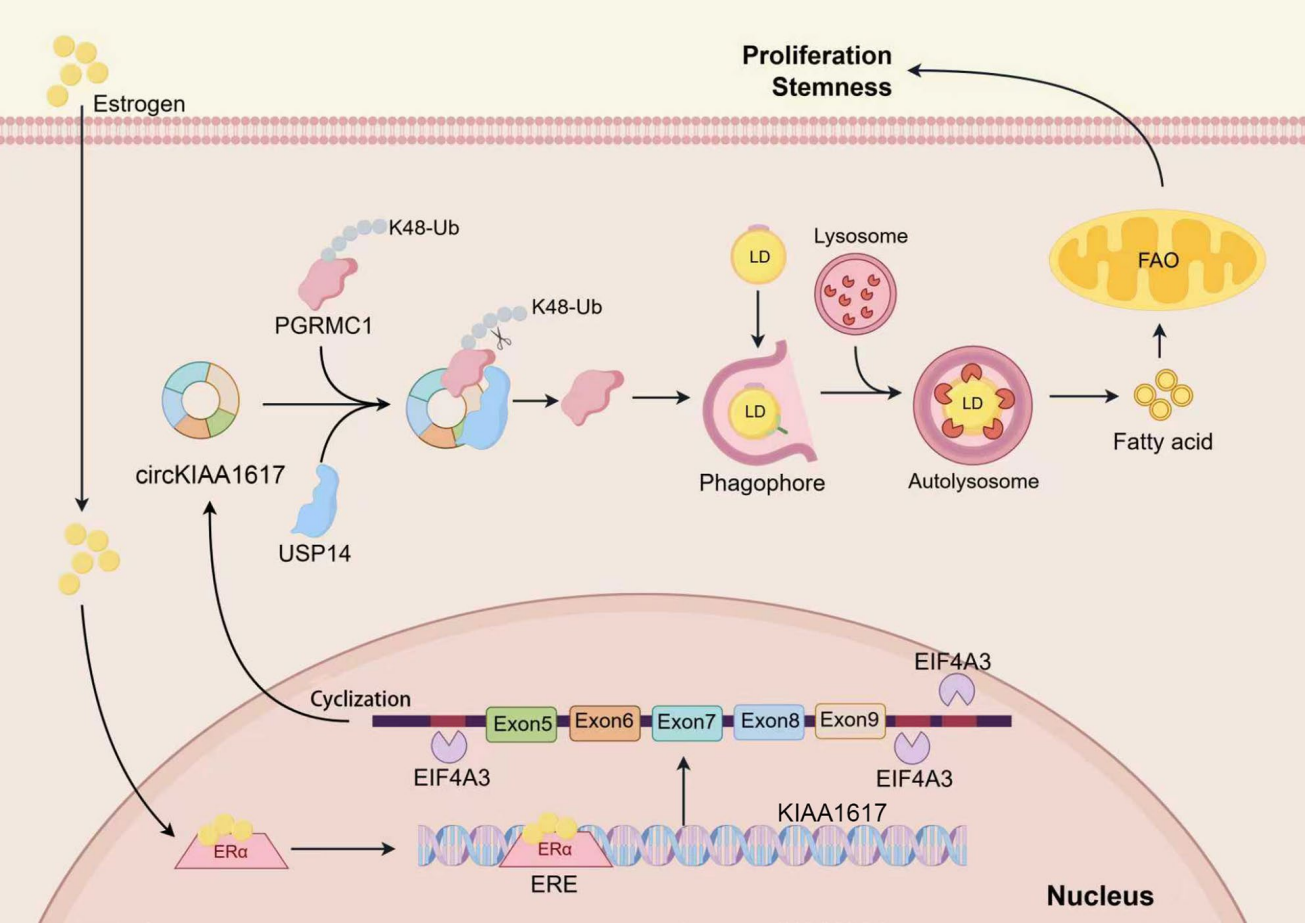
Schematic diagram showing that circKIAA1617 is transcriptionally activated by estrogen and cyclized by EIF4A3 to modulate the interaction between USP14 and PGRCM1, which increases PGRMC1 stabilization via USP14-mediated K48-linked deubiquitination, further promoting autophagy to facilitate proliferation, stemness and lipid metabolic reprogramming in ER-positive BC

In summary, we demonstrated that circKIAA1617 promoted the proliferation and stemness of ER-positive BC cells by inducing autophagy. Mechanistically, circKIAA1617 transcription was upregulated by estrogen and it was cyclized by EIF4A3. Moreover, circKIAA1617 functioned as a scaffold to enhance the interaction between PGRMC1 and USP14, enabling the K48-linked deubiquitination of PGRMC1 at lysine 105 to stabilize its expression, which accounted for circKIAA1617-induced autophagy, proliferation, and stemness of ER-positive BC cells. In addition, we demonstrated that autophagy induced by the circKIAA1617/USP14/PGRMC1 axis modulates lipid metabolic reprogramming in ER-positive BC by enhancing lipophagy. Clinical analyses revealed that circKIAA1617 is an independent prognostic biomarker correlated with a poor prognosis for patients with ER-positive BC. Collectively, our findings provide mechanistic insights into ER-positive BC proliferation and stemness through the circKIAA1617/USP14/PGRMC1 axis, positioning it as a potential diagnostic biomarker for the treatment of patients with ER-positive BC (Fig. 9).

## Supplementary Information

Supplementary Material 1.

Supplementary Material 2.

Supplementary Material 3.

Supplementary Material 4.

## Data Availability

No datasets were generated or analysed during the current study.

## References

[1] Bray F, et al. Global cancer statistics 2022: GLOBOCAN estimates of incidence and mortality worldwide for 36 cancers in 185 countries. CA Cancer J Clin. 2024;74(3):229–63. 38572751 10.3322/caac.21834

[2] Aggelis V, Johnston SRD. Advances in Endocrine-Based therapies for Estrogen Receptor-Positive metastatic breast cancer. Drugs. 2019;79(17):1849–66. 31630379 10.1007/s40265-019-01208-8

[3] Artham S, et al. Estrogen signaling suppresses tumor-associated tissue eosinophilia to promote breast tumor growth. Sci Adv. 2024;10(39):eadp2442. 39331714 10.1126/sciadv.adp2442 PMC11430468

[4] Kulkoyluoglu-Cotul E, Arca A, Madak-Erdogan Z. Crosstalk between Estrogen signaling and breast cancer metabolism. Trends Endocrinol Metab. 2019;30(1):25–38. 30471920 10.1016/j.tem.2018.10.006

[5] Will M, et al. Therapeutic resistance to anti-oestrogen therapy in breast cancer. Nat Rev Cancer. 2023;23(10):673–85. 37500767 10.1038/s41568-023-00604-3 PMC10529099

[6] Sanger HL, et al. Viroids are single-stranded covalently closed circular RNA molecules existing as highly base-paired rod-like structures. Proc Natl Acad Sci U S A. 1976;73(11):3852–6. 1069269 10.1073/pnas.73.11.3852 PMC431239

[7] Li X, Yang L, Chen LL. The Biogenesis, Functions, and challenges of circular RNAs. Mol Cell. 2018;71(3):428–42. 30057200 10.1016/j.molcel.2018.06.034

[8] Vo JN, et al. The landscape of circular RNA in cancer. Cell. 2019;176(4):869–81. e13. 30735636 10.1016/j.cell.2018.12.021 PMC6601354

[9] Conn VM, Chinnaiyan AM, Conn SJ. Circular RNA in cancer. Nat Rev Cancer. 2024;24(9):597–613. 39075222 10.1038/s41568-024-00721-7

[10] Wang L, et al. Estrogen-induced circRNA, circPGR, functions as a CeRNA to promote Estrogen receptor-positive breast cancer cell growth by regulating cell cycle-related genes. Theranostics. 2021;11(4):1732–52. 33408778 10.7150/thno.45302 PMC7778588

[11] Du WW, et al. Induction of tumor apoptosis through a circular RNA enhancing Foxo3 activity. Cell Death Differ. 2017;24(2):357–70. 27886165 10.1038/cdd.2016.133 PMC5299715

[12] Glazar P, Papavasileiou, Rajewsky N. CircBase: a database for circular RNAs. RNA. 2014;20(11):1666–70. 25234927 10.1261/rna.043687.113 PMC4201819

[13] Jiang X, et al. EIF4A3-Induced circARHGAP29 promotes aerobic Glycolysis in Docetaxel-Resistant prostate cancer through IGF2BP2/c-Myc/LDHA signaling. Cancer Res. 2022;82(5):831–45. 34965937 10.1158/0008-5472.CAN-21-2988

[14] He Y, et al. Ultrasound-triggered microbubble destruction enhances the radiosensitivity of glioblastoma by inhibiting PGRMC1-mediated autophagy in vitro and in vivo. Mil Med Res. 2022;9(1):9. 35152910 10.1186/s40779-022-00369-0 PMC8842919

[15] Coscujuela Tarrero L, et al. Luminal breast cancer-specific circular RNAs uncovered by a novel tool for data analysis. Oncotarget. 2018;9(18):14580–96. 29581865 10.18632/oncotarget.24522 PMC5865691

[16] Zhang J, et al. The feedback loop between MTA1 and MTA3/TRIM21 modulates stemness of breast cancer in response to Estrogen. Cell Death Dis. 2024;15(8):597. 39154024 10.1038/s41419-024-06942-w PMC11330498

[17] Yang F, et al. Stabilization of MORC2 by Estrogen and antiestrogens through GPER1- PRKACA-CMA pathway contributes to Estrogen-induced proliferation and endocrine resistance of breast cancer cells. Autophagy. 2020;16(6):1061–76. 32401166 10.1080/15548627.2019.1659609 PMC7469550

[18] Sun Y, et al. Estrogen promotes stemness and invasiveness of ER-positive breast cancer cells through Gli1 activation. Mol Cancer. 2014;13:137. 24889938 10.1186/1476-4598-13-137 PMC4057898

[19] Yi SA, et al. Bioengineering approaches for the advanced organoid research. Adv Mater. 2021;33(45):e2007949. 34561899 10.1002/adma.202007949 PMC8682947

[20] Mauvezin C, Neufeld TP. Bafilomycin A1 disrupts autophagic flux by inhibiting both V-ATPase-dependent acidification and Ca-P60A/SERCA-dependent autophagosome-lysosome fusion. Autophagy. 2015;11(8):1437–8. 26156798 10.1080/15548627.2015.1066957 PMC4590655

[21] McKenna NJ, O’Malley BW. Combinatorial control of gene expression by nuclear receptors and coregulators. Cell. 2002;108(4):465–74. 11909518 10.1016/s0092-8674(02)00641-4

[22] Xu Y, et al. ERalpha is an RNA-binding protein sustaining tumor cell survival and drug resistance. Cell. 2021;184(20):5215–29. e17. 34559986 10.1016/j.cell.2021.08.036 PMC8547373

[23] Dudekula DB, et al. CircInteractome: A web tool for exploring circular RNAs and their interacting proteins and MicroRNAs. RNA Biol. 2016;13(1):34–42. 26669964 10.1080/15476286.2015.1128065 PMC4829301

[24] Mir SU, et al. Progesterone receptor membrane component 1/Sigma-2 receptor associates with MAP1LC3B and promotes autophagy. Autophagy. 2013;9(10):1566–78. 24113030 10.4161/auto.25889

[25] Knupp J, et al. The ER transmembrane protein PGRMC1 recruits misfolded proteins for reticulophagic clearance. Autophagy. 2022;18(1):228–30. 34779709 10.1080/15548627.2021.1997062 PMC8865224

[26] Zhu X, et al. PGRMC1-dependent autophagy by Hyperoside induces apoptosis and sensitizes ovarian cancer cells to cisplatin treatment. Int J Oncol. 2017;50(3):835–46. 28197632 10.3892/ijo.2017.3873

[27] Zhou WY, et al. Circular RNA: metabolism, functions and interactions with proteins. Mol Cancer. 2020;19(1):172. 33317550 10.1186/s12943-020-01286-3 PMC7734744

[28] Shi D, et al. USP14 promotes Tryptophan metabolism and immune suppression by stabilizing IDO1 in colorectal cancer. Nat Commun. 2022;13(1):5644. 36163134 10.1038/s41467-022-33285-x PMC9513055

[29] Zhao C, et al. A self-amplifying USP14-TAZ loop drives the progression and liver metastasis of pancreatic ductal adenocarcinoma. Cell Death Differ. 2023;30(1):1–15. 35906484 10.1038/s41418-022-01040-w PMC9883464

[30] Wang C, et al. GPS-Uber: a hybrid-learning framework for prediction of general and E3-specific lysine ubiquitination sites. Brief Bioinform. 2022;23(2):1–15. 10.1093/bib/bbab574 35037020

[31] Arnal JF, et al. Membrane and nuclear Estrogen receptor alpha actions: from tissue specificity to medical implications. Physiol Rev. 2017;97(3):1045–87. 28539435 10.1152/physrev.00024.2016

[32] Vrtacnik P, et al. The many faces of Estrogen signaling. Biochem Med (Zagreb). 2014;24(3):329–42. 25351351 10.11613/BM.2014.035 PMC4210253

[33] Kumar S, et al. Estrogen-dependent DLL1-mediated Notch signaling promotes luminal breast cancer. Oncogene. 2019;38(12):2092–107. 30442981 10.1038/s41388-018-0562-z PMC6756232

[34] Chi R, et al. Estrogen-induced circFAM171A1 regulates sheep myoblast proliferation through the oar-miR-485-5p/MAPK15/MAPK pathway. Cell Mol Life Sci. 2025;82(1):123. 40105989 10.1007/s00018-025-05639-3 PMC11923336

[35] Liu W, et al. TRIM22 inhibits osteosarcoma progression through destabilizing NRF2 and thus activation of ROS/AMPK/mTOR/autophagy signaling. Redox Biol. 2022;53:102344. 35636015 10.1016/j.redox.2022.102344 PMC9144049

[36] Kao CH, et al. TFEB- and TFE3-dependent autophagy activation supports cancer proliferation in the absence of centrosomes. Autophagy. 2022;18(12):2830–50. 35316161 10.1080/15548627.2022.2051880 PMC9673955

[37] Liu D, et al. Hypoxia-induced galectin-8 maintains stemness in glioma stem cells via autophagy regulation. Neuro Oncol. 2024;26(5):872–88. 38158714 10.1093/neuonc/noad264 PMC11066898

[38] Thomas C, Gustafsson JA. Estrogen receptor mutations and functional consequences for breast cancer. Trends Endocrinol Metab. 2015;26(9):467–76. 26183887 10.1016/j.tem.2015.06.007

[39] Fang Z, et al. Is an Estrogen-Responsive LncRNA that drives breast cancer through the E2F1/RB1 pathway. Cancer Res. 2020;80(20):4399–413. 32826278 10.1158/0008-5472.CAN-20-1031 PMC7572695

[40] Wang Y, et al. SGK3 is an estrogen-inducible kinase promoting estrogen-mediated survival of breast cancer cells. Mol Endocrinol. 2011;25(1):72–82. 21084382 10.1210/me.2010-0294 PMC3089033

[41] Lin Y, et al. Systematic analysis of gene expression alteration and Co-Expression network of eukaryotic initiation factor 4A-3 in cancer. J Cancer. 2018;9(24):4568–77. 30588240 10.7150/jca.27655 PMC6299400

[42] Liang Y, et al. Exosomal circSIPA1L3-mediated intercellular communication contributes to glucose metabolic reprogramming and progression of triple negative breast cancer. Mol Cancer. 2024;23(1):125. 38849860 10.1186/s12943-024-02037-4 PMC11161950

[43] Zheng X, et al. The circrna circSEPT9 mediated by E2F1 and EIF4A3 facilitates the carcinogenesis and development of triple-negative breast cancer. Mol Cancer. 2020;19(1):73. 32264877 10.1186/s12943-020-01183-9 PMC7137343

[44] Cancer Genome Atlas N. Comprehensive molecular portraits of human breast tumours. Nature. 2012;490(7418):61–70. 23000897 10.1038/nature11412 PMC3465532

[45] Li B, et al. circNDUFB2 inhibits non-small cell lung cancer progression via destabilizing IGF2BPs and activating anti-tumor immunity. Nat Commun. 2021;12(1):295. 33436560 10.1038/s41467-020-20527-z PMC7804955

[46] Bahrulolum H, et al. CircRNA: unlocking new frontiers in therapeutic and vaccine development. Mol Ther. 2025.S1525-0016(25)00859-7. Advance online publication. 10.1016/j.ymthe.2025.10.038 PMC12882336 41109952

[47] McGuire MR, Espenshade PJ. PGRMC1: an enigmatic heme-binding protein. Pharmacol Ther. 2023;241:108326. 36463977 10.1016/j.pharmthera.2022.108326 PMC9839567

[48] Ruan X, et al. Association of Circulating progesterone receptor membrane Component-1 (PGRMC1) with breast tumor characteristics and comparison with known tumor markers. Menopause. 2020;27(2):183–93. 31876619 10.1097/GME.0000000000001436

[49] Rohe HJ, et al. PGRMC1 (progesterone receptor membrane component 1): a targetable protein with multiple functions in steroid signaling, P450 activation and drug binding. Pharmacol Ther. 2009;121(1):14–9. 18992768 10.1016/j.pharmthera.2008.09.006 PMC2659782

[50] Zhao Y, et al. PGRMC1 promotes triple-negative breast cancer cell growth via suppressing ferroptosis. Climacteric. 2023;26(2):135–42. 36724820 10.1080/13697137.2023.2170225

[51] Guan A, et al. PGRMC1 promotes NSCLC stemness phenotypes by disrupting TRIM56-mediated ubiquitination of AHR. Biochim Biophys Acta Mol Basis Dis. 2024;1870(7):167440. 39059592 10.1016/j.bbadis.2024.167440

[52] Nijman SM, et al. A genomic and functional inventory of deubiquitinating enzymes. Cell. 2005;123(5):773–86. 16325574 10.1016/j.cell.2005.11.007

[53] Liu B, et al. Proteome-wide analysis of USP14 substrates revealed its role in hepatosteatosis via stabilization of FASN. Nat Commun. 2018;9(1):4770. 30425250 10.1038/s41467-018-07185-y PMC6233205

[54] You L, et al. SDC2 stabilization by USP14 promotes gastric cancer progression through Co-option of PDK1. Int J Biol Sci. 2023;19(11):3483–98. 37496999 10.7150/ijbs.84331 PMC10367555

[55] Sun T, Liu Z, Yang Q. The role of ubiquitination and deubiquitination in cancer metabolism. Mol Cancer. 2020;19(1):146. 33004065 10.1186/s12943-020-01262-x PMC7529510

[56] Liu S, et al. Autophagy: regulator of cell death. Cell Death Dis. 2023;14(10):648. 37794028 10.1038/s41419-023-06154-8 PMC10551038

[57] Cheng C, et al. Lipid metabolism reprogramming and its potential targets in cancer. Cancer Commun (Lond). 2018;38(1):27. 29784041 10.1186/s40880-018-0301-4 PMC5993136

[58] Yang Y, et al. Interplay of CD36, autophagy, and lipid metabolism: insights into cancer progression. Metabolism. 2024;155:155905. 38548128 10.1016/j.metabol.2024.155905

[59] Lin Z, et al. The lipid basis of cell death and autophagy. Autophagy. 2024;20(3):469–88. 37768124 10.1080/15548627.2023.2259732 PMC10936693

[60] Li Q, et al. Impaired lipophagy induced-microglial lipid droplets accumulation contributes to the buildup of TREM1 in diabetes-associated cognitive impairment. Autophagy. 2023;19(10):2639–56. 37204119 10.1080/15548627.2023.2213984 PMC10472854

[61] Zhang S, et al. The regulation, function, and role of lipophagy, a form of selective autophagy, in metabolic disorders. Cell Death Dis. 2022;13(2):132. 35136038 10.1038/s41419-022-04593-3 PMC8825858

[62] Laval T, Ouimet M. A role for lipophagy in atherosclerosis. Nat Rev Cardiol. 2023;20(7):431–2. 37161064 10.1038/s41569-023-00885-z PMC10169197

[63] Haidar M, et al. Lipophagy: a new player in CNS disorders. Trends Endocrinol Metab. 2021;32(11):941–51. 34561114 10.1016/j.tem.2021.08.010

[64] Panda PK, et al. Deacetylation of LAMP1 drives lipophagy-dependent generation of free fatty acids by abrus agglutinin to promote senescence in prostate cancer. J Cell Physiol. 2020;235(3):2776–91. 31544977 10.1002/jcp.29182

[65] Shao Y, et al. The feedback loop of AURKA/DDX5/TMEM147-AS1/let-7 drives lipophagy to induce cisplatin resistance in epithelial ovarian cancer. Cancer Lett. 2023;565:216241. 37217070 10.1016/j.canlet.2023.216241

[66] You JH, Lee J, Roh JL. PGRMC1-dependent lipophagy promotes ferroptosis in paclitaxel-tolerant persister cancer cells. J Exp Clin Cancer Res. 2021;40(1):350. 34749765 10.1186/s13046-021-02168-2 PMC8573965

[67] Divakaruni AS, et al. Etomoxir inhibits macrophage polarization by disrupting coa homeostasis. Cell Metab. 2018;28(3):490–503. e7. 30043752 10.1016/j.cmet.2018.06.001 PMC6125190

[68] He Z, et al. Autophagy-associated circrna circATG7 facilitates autophagy and promotes pancreatic cancer progression. Cell Death Dis. 2022;13(3):233. 35288538 10.1038/s41419-022-04677-0 PMC8921308

[69] Liang G, et al. Autophagy-associated circrna circcdyl augments autophagy and promotes breast cancer progression. Mol Cancer. 2020;19(1):65. 32213200 10.1186/s12943-020-01152-2 PMC7093993

[70] Zhou Y, et al. CircEPS15, as a sponge of MIR24-3p ameliorates neuronal damage in Parkinson disease through boosting PINK1-PRKN-mediated mitophagy. Autophagy. 2023;19(9):2520–37. 37014258 10.1080/15548627.2023.2196889 PMC10392753

[71] Xiang J, et al. How does Estrogen work on autophagy? Autophagy. 2019;15(2):197–211. 30208759 10.1080/15548627.2018.1520549 PMC6333457

[72] Zhao Y, et al. The estrogen-autophagy axis: insights into cytoprotection and therapeutic potential in cancer and infection. Int J Mol Sci. 2024;25(23):12576. 10.3390/ijms252312576 PMC11641569 39684286

