# Supplementary material for: CircKIAA1617 promotes stemness via USP14/PGRMC1-mediated autophagy and lipid metabolism reprogramming in ER-positive breast cancer
Source: Mol Cancer. 2026 Jan 31;25:55. doi: 10.1186/s12943-026-02580-2 (PMC12952063; doi:10.1186/s12943-026-02580-2)
Supplement: Supplementary file 1 — Supplementary Material 1. [file 12943_2026_2580_MOESM1_ESM.docx]

**Supplementary Figures**


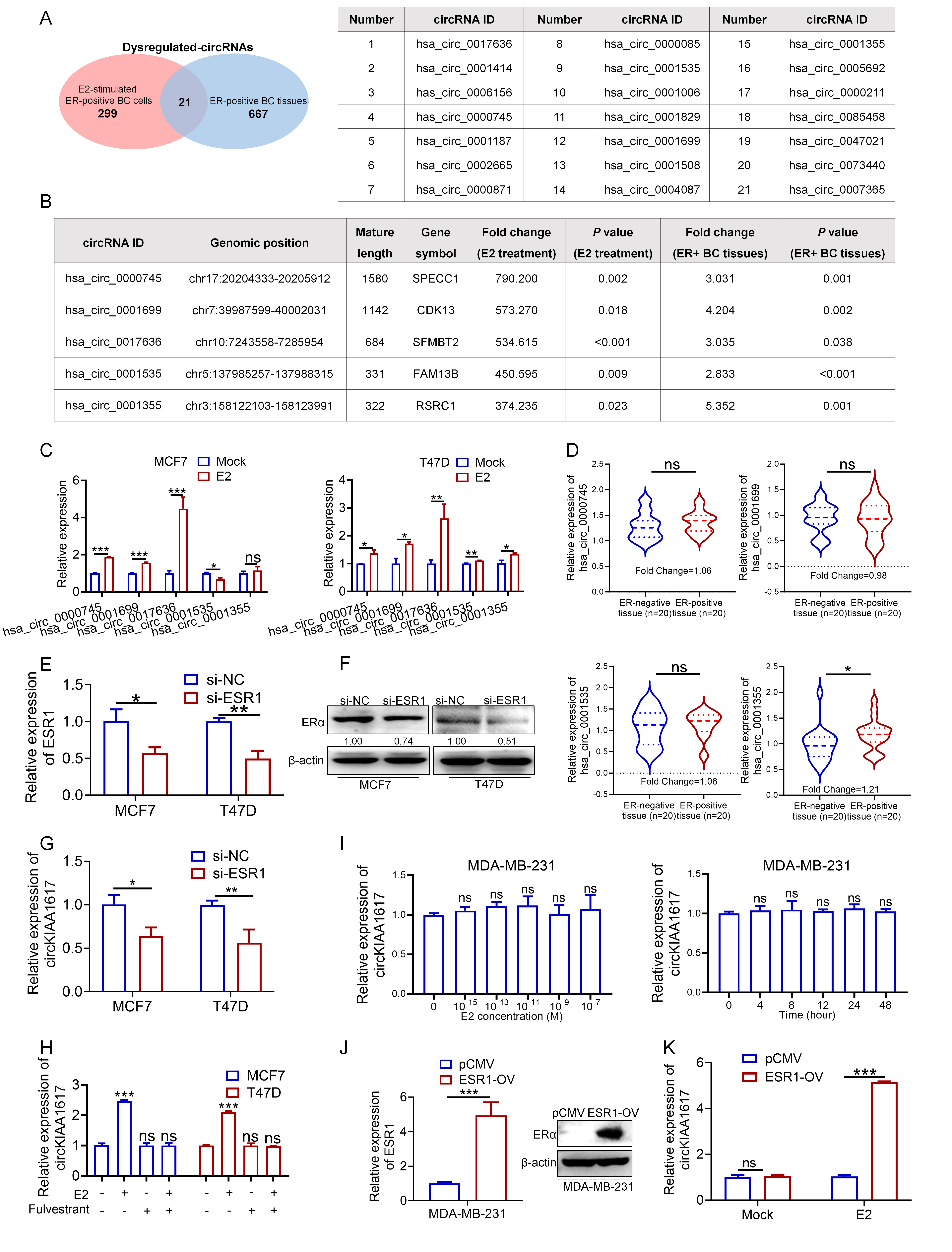


**Figure S1.** **A.** Overlapping dysregulated circRNAs in E2-stimulated ER-positive BC cells and ER-positive BC tissues. **B.** The top 5 circRNAs among the 21 circRNAs whose expression was upregulated in E2-treated MCF7 cells were selected, and the basic information for these circRNAs is shown. **C.** The expression of the 5 circRNAs in both MCF7 and T47D cells was examined after estrogen treatment (1 nM) (n=3). **D.** The expression of hsa_circ_0000745, hsa_circ_0001699, hsa_circ_0001535 and hsa_circ_0001355 in ER-negative and ER-positive BC (n=20 in each group). The efficiency of specific siRNAs targeting ESR1 was measured by qRT-PCR (**E**) and western blotting (**F**) (n=3). **G.** Effects of the ESR1 siRNA on circKIAA1617 expression (n=3). **H.** qRT-PCR was used to measure the expression of circKIAA1617 after estrogen and fulvestrant treatment (n=3). **I.** The expression levels of circKIAA1617 in the MDA-MB-231 cell line treated with different concentrations of estrogen for different periods (n=3). **J.** The efficiency of ESR1 overexpression in MDA-MB-231 cells was measured using qRT-PCR and western blotting (n=3). **K.** qRT-PCR was performed to measure the expression of circKIAA1617 in MDA-MB-231 cells following ESR1 overexpression and estrogen treatment (n=3). ns, not significant; *P < 0.05; **P < 0.01; and ***P < 0.001.


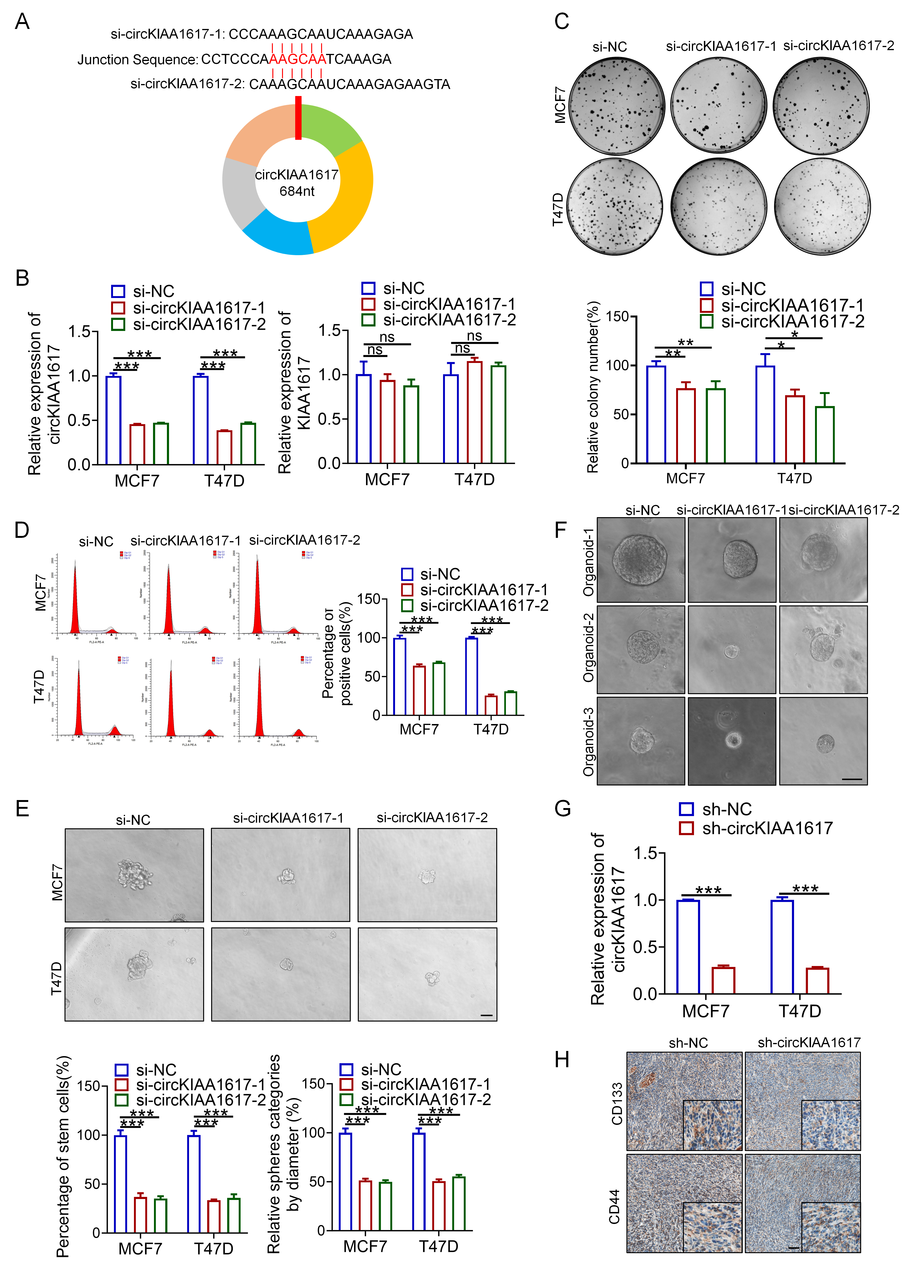


**Figure S2.** **A.** Schematic of siRNA sequences designed against the circKIAA1617 back-splice junction. **B.** Efficiency of circKIAA1617 siRNAs against circKIAA1617 and the KIAA1617 mRNA, as determined by Qrt-PCR (n=3). Colony formation (**C**) and flow cytometry (**D**) assays were used to determine the effect of circKIAA1617 knockdown on ER-positive BC cell proliferation (n=3). **E.** Sphere formation assays were performed to determine the effect of circKIAA1617 silencing on the stemness of MCF7 and T47D cells (n=3). Scale bars=100 μm. **F.** Patient-derived organoid (PDO) models were generated after cells were transfected with the circKIAA1617 siRNA (n=3). Scale bars=100 μm. **G.** The efficiency of sh-circKIAA1617 was assessed by qRT-PCR (n=3). **H.** IHC assays were performed to detect the expression levels of CD44 and CD133 after circKIAA1617 silencing (n=3). Scale bars=100 μm. ns, not significant; *P < 0.05; **P < 0.01; and ***P < 0.001.


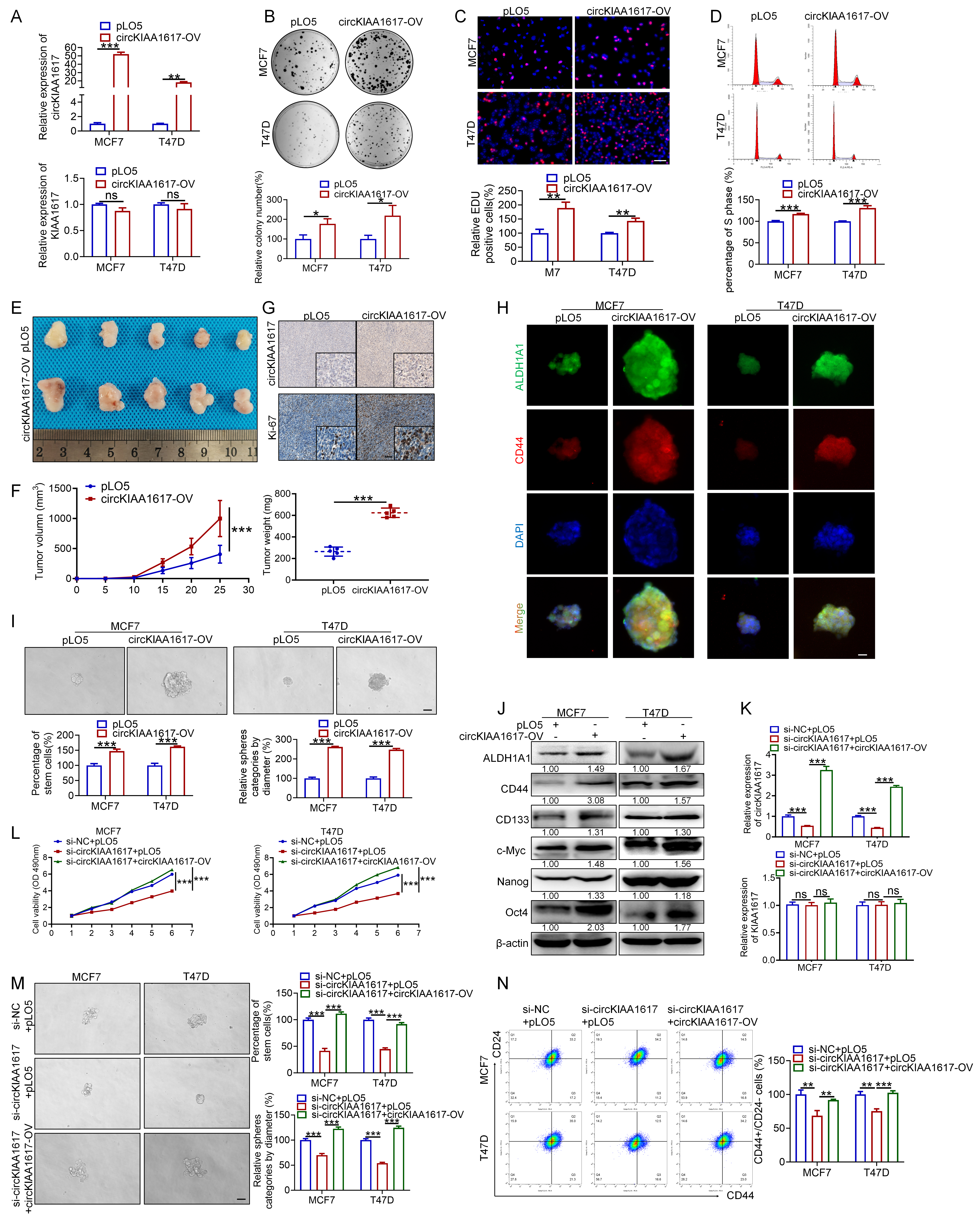


**F****igure S3.** **A.** qRT-PCR was conducted to measure the expression of circKIAA1617 and the KIAA1617 mRNA in MCF7 and T47D cells following circKIAA1617 overexpression (n=3). Colony formation (**B**), EdU (**C**) and flow cytometry assays (**D**) were performed to evaluate the proliferation of MCF7 and T47D cells after circKIAA1617 overexpression. Scale bars=100 μm (n=3). **E, F.** The growth of tumors was monitored after the subcutaneous implantation of MCF7 cells stably overexpressing circKIAA1617. **G.** IHC assays were performed to detect the expression levels of CD44 and CD133 in tumors (n=3). Scale bars=100 μm. **H.** IF staining was used to determine the effects of circKIAA1617 overexpression on the expression of stemness markers (CD44 and ALDH1A1) in the MCF7 and T47D spheres (n=3). Scale bars=20 μm. Sphere formation assays (**I**) and western blot analysis (**J**) were performed to assess the stemness of ER-positive BC cells (n=3). Scale bars=100 μm. **K.** qRT-PCR was used to measure the expression of circKIAA1617 and the KIAA1617 mRNA in MCF7 and T47D cells with circKIAA1617 overexpression following circKIAA1617 silencing (n=3). MTT (**L**), sphere formation (**M**) and flow cytometry (**N**) assays were performed in ER-positive BC cell lines with circKIAA1617 overexpression after interference (n=3). Scale bars=100 μm. ns, not significant; *P < 0.05; **P < 0.01; and ***P < 0.001.


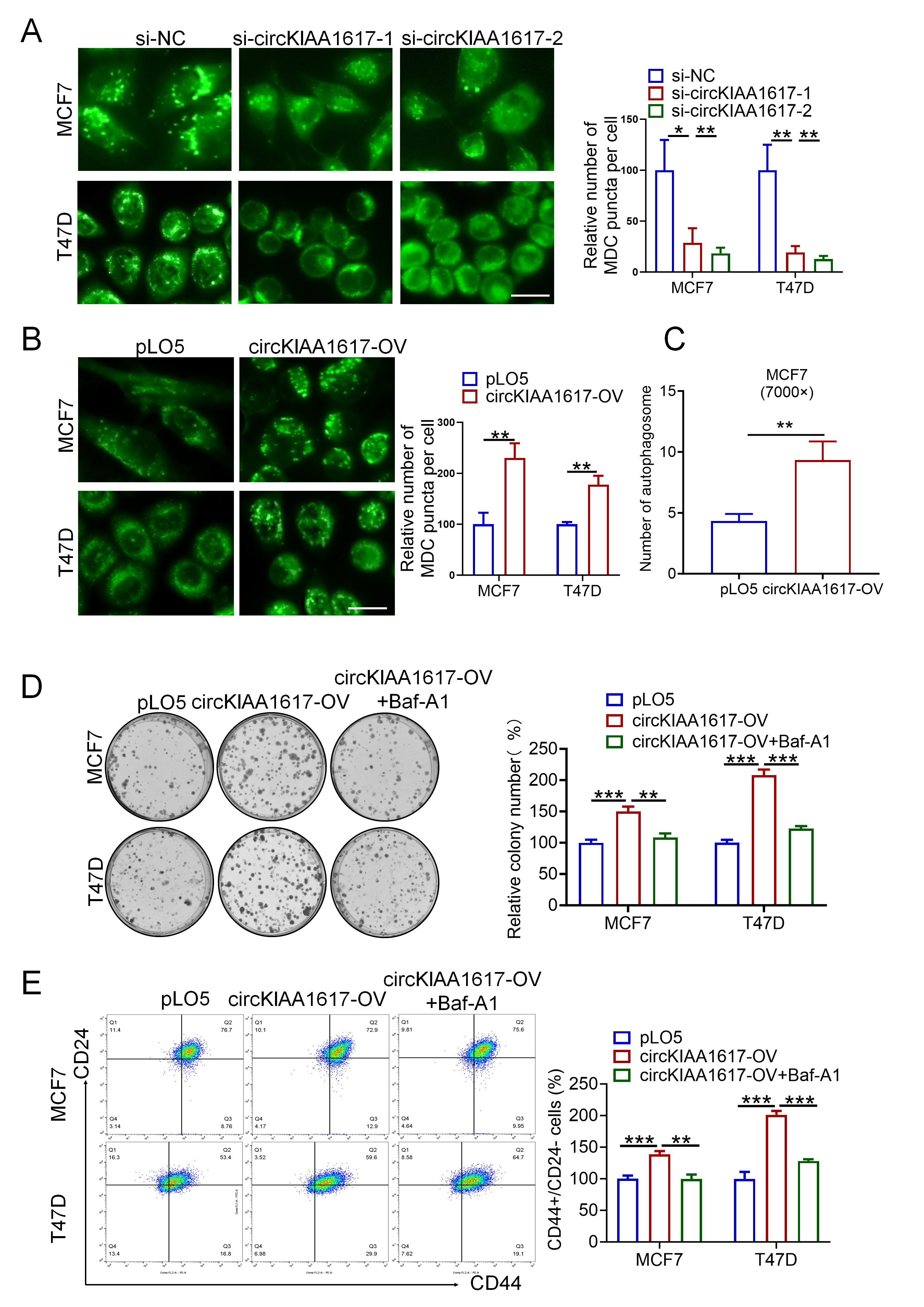


**Figure S4.** MDC assays were performed to evaluate the effects of circKIAA1617 knockdown (**A**) or overexpression (**B**) on autophagy flux in ER-positive BC cells (n=3). Scale bars=20 μm. **C.** The number of autophagosomes in circKIAA1617-overexpressing ER-positive BC cells determined using electron microscopy (n=3). The effects of Baf-A1 (50 nM) on the circKIAA1617-induced proliferation and stemness of ER-positive BC cells were examined by performing MTT (**D**) and flow cytometry (**E**) assays (n=3). *P < 0.05; **P < 0.01; and ***P < 0.001.


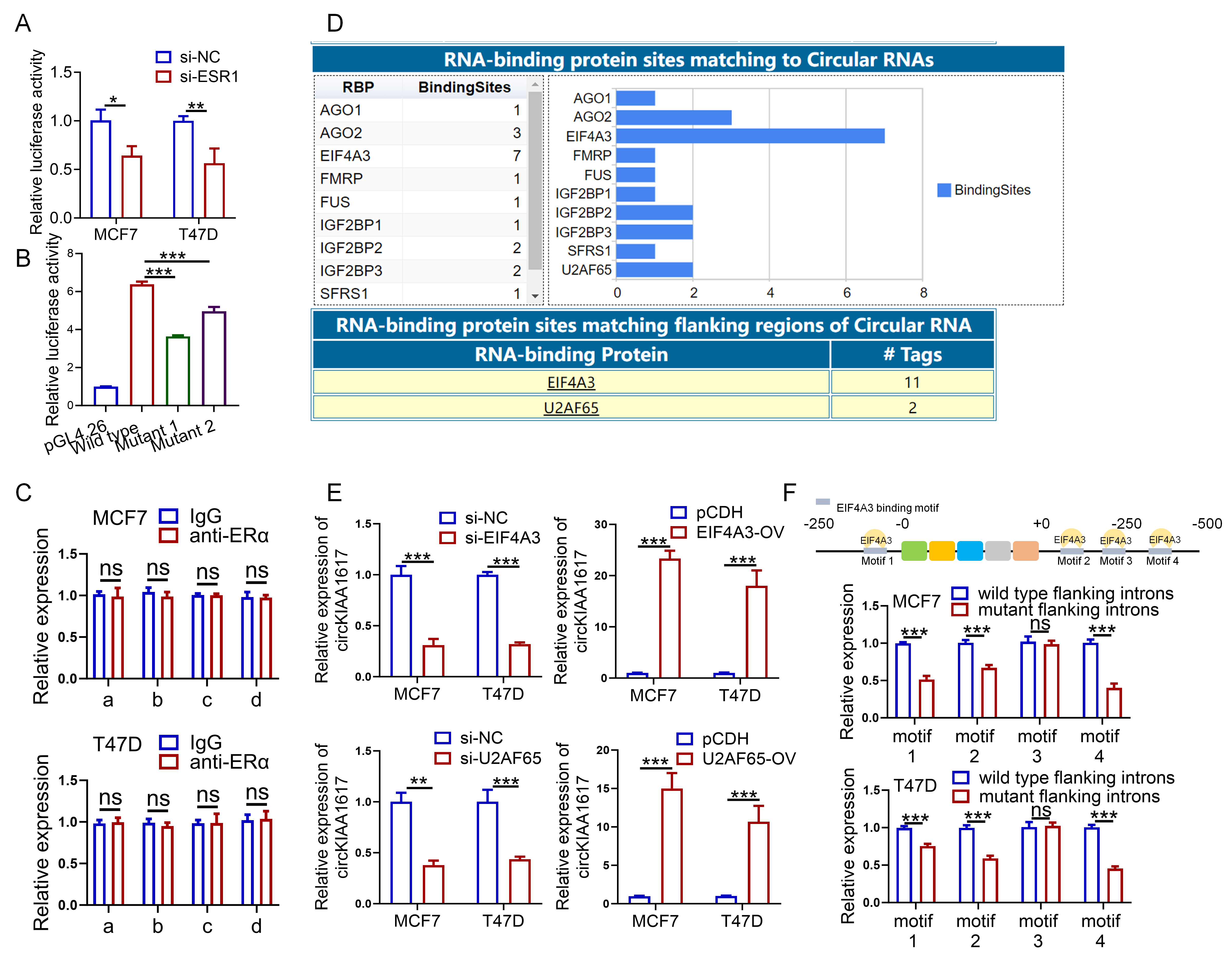


**F****igure S5. A.** Effects of the ESR1 siRNA on the transcriptional activity of the circKIAA1617 promoter (n=3). **B.** Luciferase activity was measured with a pGL4.26 vector containing wild-type or mutant circKIAA1617 promoters (n=3). **C.** RIP assays were utilized to detect the binding between ERα and flanking introns (n=3). **D.** The RNA-binding proteins matching the flanking regions of circKIAA1617 were predicted by the CircInteractome database. **E.** The efficiency of EIF4A3 and U2AF65 overexpression or knockdown was measured by qRT-PCR (n=3). **F.** Upper panel, predicted binding motifs of EIF4A3 within the flanking introns of circKIAA1617; lower panel, RIP assays were performed to validate the binding between EIF4A3 and the predicted motifs (n=3). ns, not significant; *P < 0.05; **P < 0.01; and ***P < 0.001.


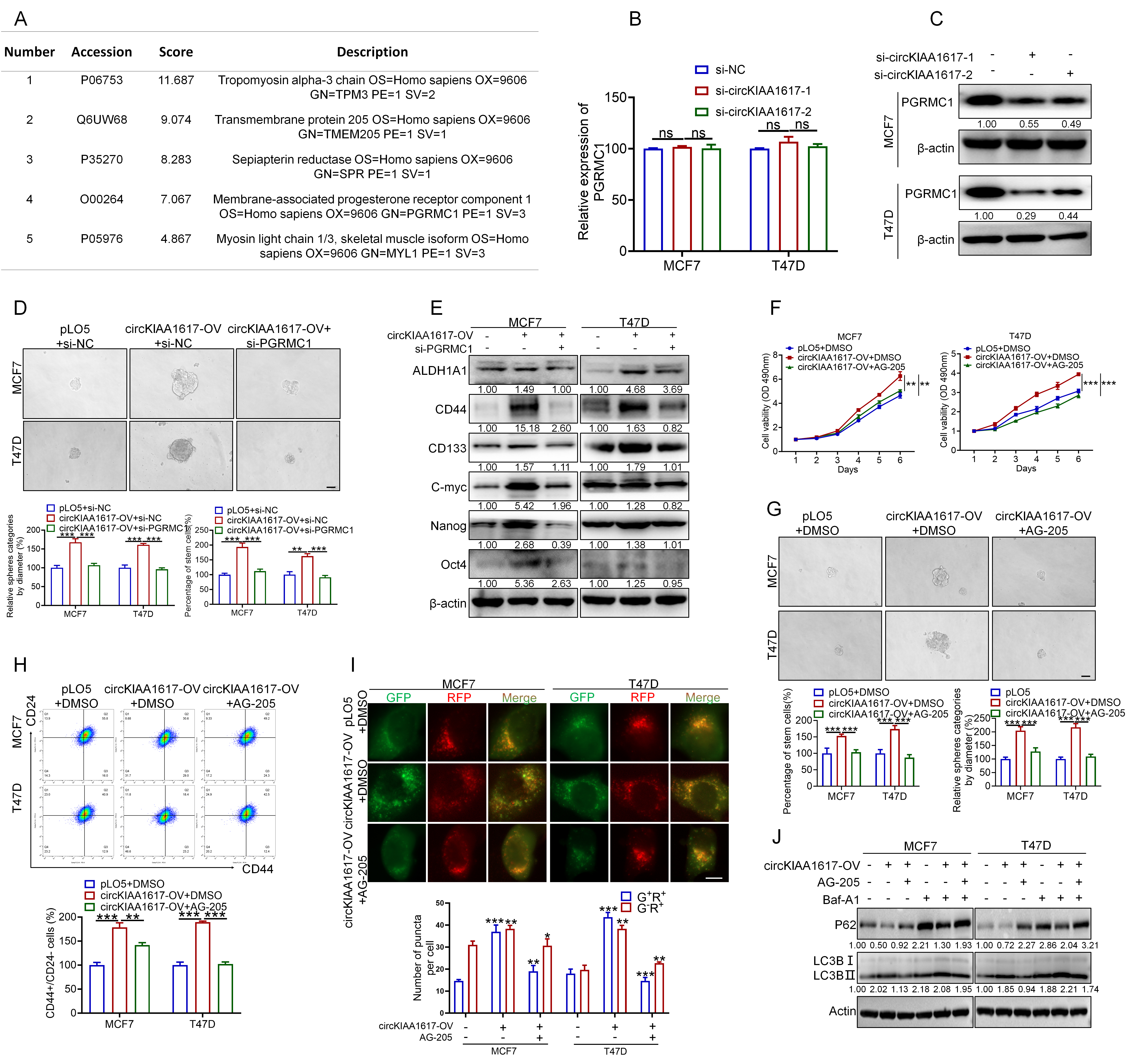


**Figure S6. A.** The top five enriched proteins identified by the RNA pull-down assay. **B.** The influence of circKIAA1617 knockdown on PGRMC1 mRNA expression was evaluated by qRT-PCR (n=3). **C.** Western blot assays were performed to evaluate the expression of the PGRMC1 protein after circKIAA1617 silencing (n=3). The effects of PGRMC1 knockdown on circKIAA1617-induced stemness were detected by performing tumor sphere formation (**D**) and western blot (**E**) assays (n=3). Scale bars=100 μm. MTT (**F**), sphere formation (**G**) and flow cytometry (**H**) assays revealed the effects of AG-205 (10 μM) on the proliferation and stemness of ER-positive BC cells overexpressing circKIAA1617 (n=3). Scale bars=100 μm. **I.** mCherry-GFP-LC3B was transfected to assess autophagy flux in MCF7 and T47D cells after circKIAA1617 overexpression and treatment with or without AG-205 (n=3). Scale bars=10 μm. **J.** Western blot analysis of the levels of autophagy-associated proteins after circKIAA1617 overexpression and AG-205 treatment (n=3). ns, not significant; **P < 0.01; and ***P < 0.001.


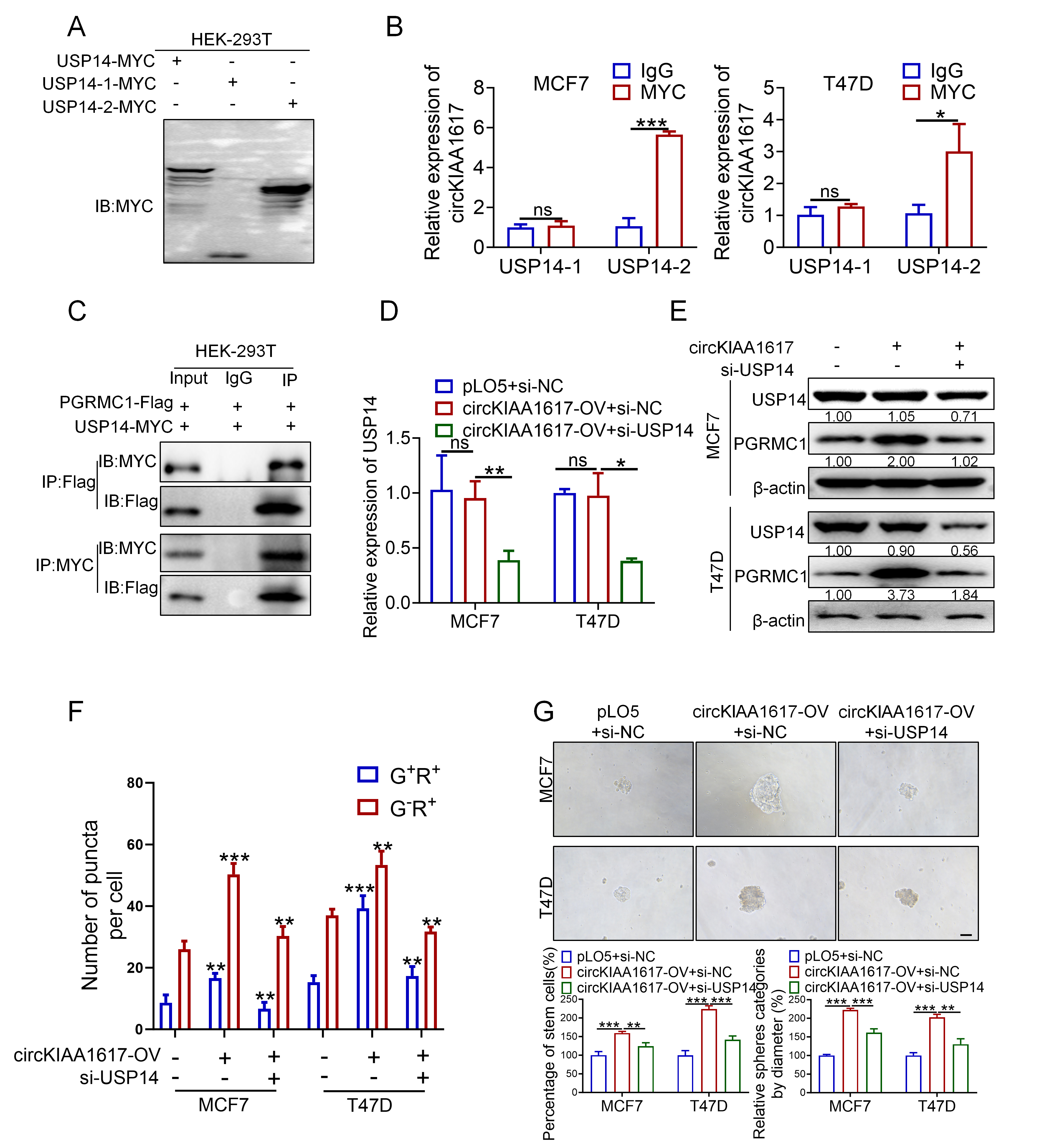


**Figure S7. A.** Efficiency of the expression of the full-length and truncated USP14 constructs (n=3). **B.** RIP assays revealed the specific domain of USP14 required for the circKIAA1617 interaction (n=3). **C.** Co-IP assays demonstrated the interaction between PGRMC1 and USP14 (n=3). The effects of circKIAA1617 overexpression and USP14 knockdown on PGRMC1 expression were measured using qRT-PCR (**D**) and western blot assays (**E**) (n=3). **F.** The effects of circKIAA1617 overexpression and USP14 knockdown on autophagosome formation in ER-positive BC cells were evaluated (n=3). **G.** Effect of USP14 knockdown on the circKIAA1617-induced stemness of ER-positive BC cells (n=3). Scale bars=100 μm. ns, not significant; *P < 0.05; **P < 0.01; and ***P < 0.001.


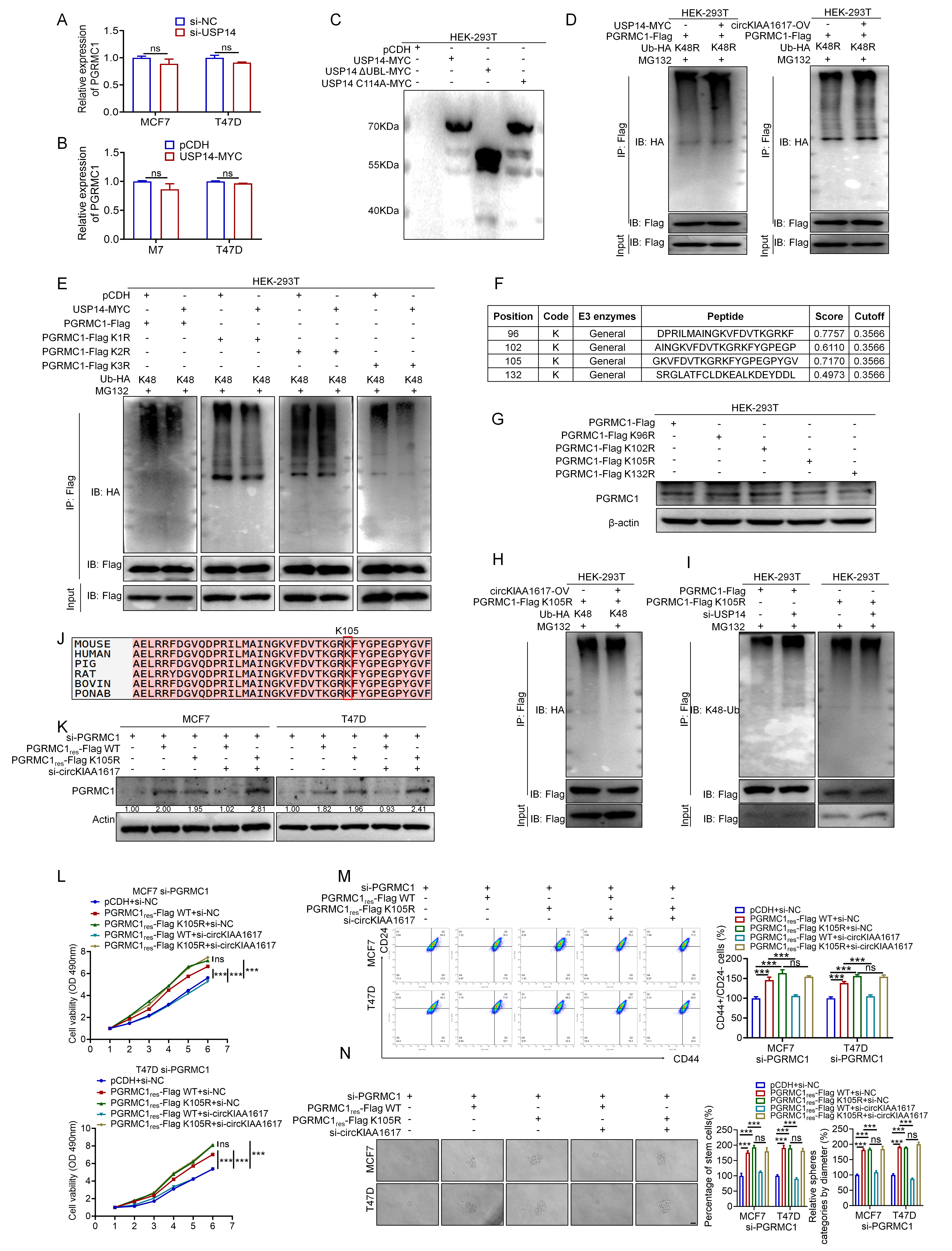


**F****igure S8.** The expression of the PGRMC1 mRNA following USP14 knockdown (**A**) and overexpression (**B**) was measured by qRT-PCR (n=3). **C**. Efficiency of the USP14-ΔUBL truncation vector and C114A mutant vector (n=3). **D.** Co-IP and western blotting confirmed that circKIAA1617 and USP14 facilitated the K48-linked deubiquitination of PGRMC1 (n=3). **E.** The PGRMC1 protein was divided into three segments (residues 1-71aa, 72-171aa, and 172-195aa), and all lysine residues within each segment were mutated (K1R, K2R, K3R). Immunoprecipitation and immunoblotting confirmed that the deubiquitinated domain of the PGRMC1 protein by USP14 was located on the cytochrome b5-like domain (72-171aa) (n=3). **F.** The PGRMC1 protein sequence was predicted by GPS-UBER, and four high-confidence lysine sites (K96, K102, K105 and K132) within the cytochrome b5-like domain were predicted. **G.** Efficiency of the PGRMC1 K96R, K102R, K105R and K132R overexpression vectors (n=3). **H.** Co-IP and western blot assays confirmed that circKIAA1617 promoted the K48-linked deubiquitination of PGRMC1 at lysine 105 (n=3). **I.** Co-IP and western blot assays with a K48-Ub-specific antibody revealed that USP14 modulated the level of the K48-linked ubiquitination of PGRMC1 at lysine 105 (n=3). **J.** Conservation of the PGMRC1 lysine 105 site across different species. **K.** The expression of wild-type (PGRMC1_res_-Flag WT) and K105R (PGRMC1_res_-Flag K105R) siRNA-resistant PGRMC1 vectors in MCF7 and T47D cells with circKIAA1617 knockdown (n=3). **L-N**. Proliferation and stemness were examined after PGRMC1_res_-Flag WT and PGRMC1_res_-Flag K105R overexpression with or without circKIAA1617 knockdown in MCF7 and T47D cells (n=3). Scale bars=100 μm. ns, not significant; ***P < 0.001.


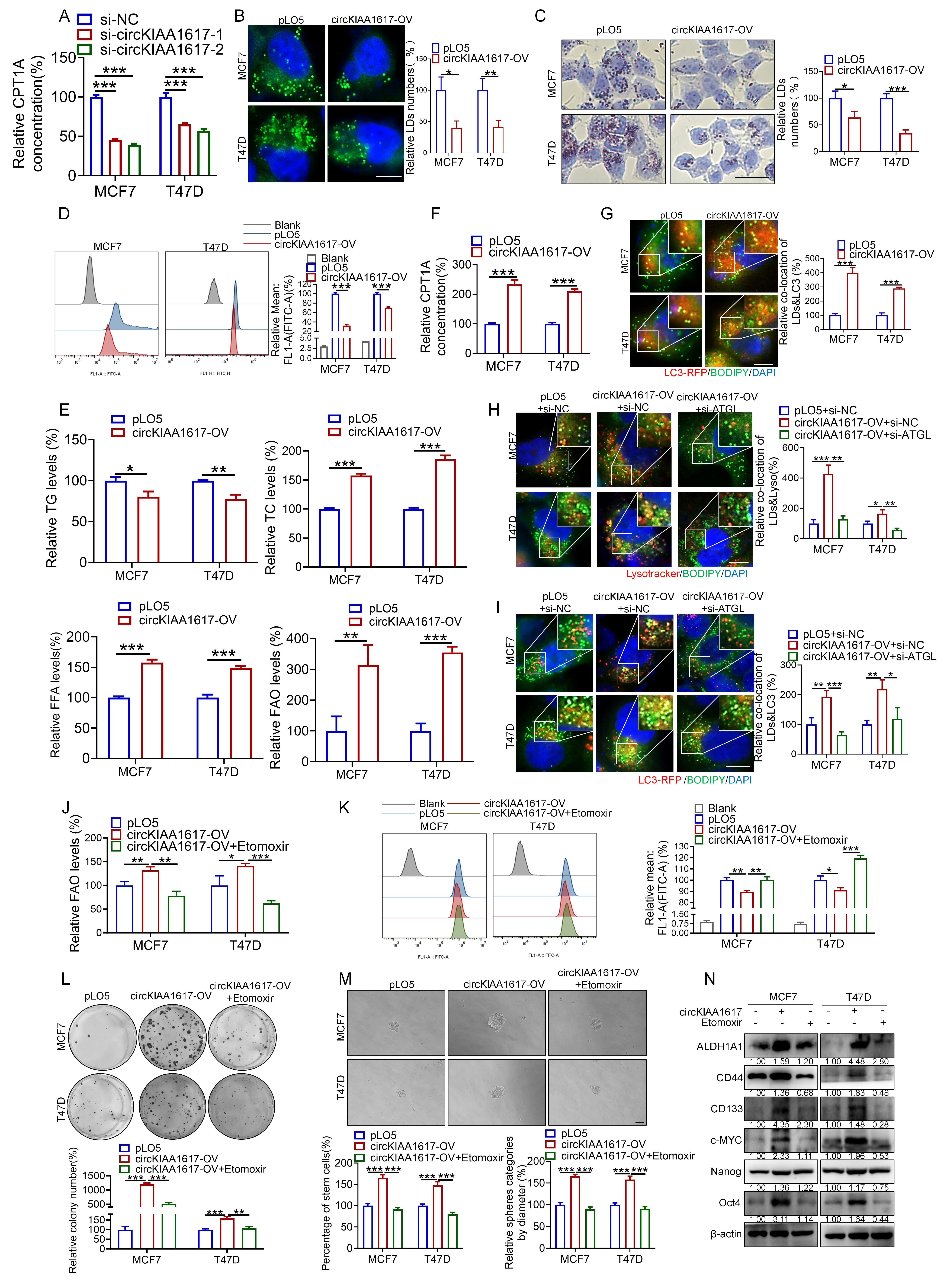


**Figure S9. A.** The concentration of CPT1A was determined by ELISA after circKIAA1617 interference (n=3). BODIPY (**B**) and Oil red O (**C**) staining were performed to determine the number of LDs in MCF7 and T47D cells after circKIAA1617 overexpression (n=3). Scale bars for BODIPY staining=10 μm. Scale bars for Oil red O staining=50 μm. **D.** Flow cytometry assays were conducted to assess the number of LDs in MCF7 and T47D cells after the overexpression of circKIAA1617 (n=3). **E.** TG, TC, FFA and FAO levels in ER-positive BC cells were measured after transfection with the circKIAA1617-OV vector (n=3). **F.** CPT1A levels were assessed by ELISA following the overexpression of circKIAA1617 (n=3). **G.** The colocalization of LC3 and LDs was examined in MCF7 and T47D cells overexpressing circKIAA1617 (n=3). **H, I.** LD colocalization with lysosomes and LC3 were evaluated in rescue experiments involving circKIAA1617 overexpression combined with ATGL knockdown (n=3). Scale bars=100 μm. **J.** Etomoxir was used to treat circKIAA1617-overexpressing ER-positive BC cells, and the FAO values were determined in MCF7 and T47D cells (n=3). **K.** Flow cytometry assays were performed to assess the number of LDs in MCF7 and T47D cells after circKIAA1617 overexpression and etomoxir treatment (n=3). **L.** The proliferation of MCF7 and T47D cells with circKIAA1617 overexpression and etomoxir treatment was measured by performing colony formation assays (n=3). The stemness of ER-positive BC cells with circKIAA1617 overexpression and etomoxir treatment was measured by performing tumor sphere formation (**M**) and western blot (**N**) assays (n=3). Scale bars=100 μm. *P < 0.05; **P < 0.01; and ***P < 0.001.
