# Supplementary material for: CircKIAA1617 promotes stemness via USP14/PGRMC1-mediated autophagy and lipid metabolism reprogramming in ER-positive breast cancer
Source: Mol Cancer. 2026 Jan 31;25:55. doi: 10.1186/s12943-026-02580-2 (PMC12952063; doi:10.1186/s12943-026-02580-2)
Supplement: Supplementary file 2 — Supplementary Material 2. [file 12943_2026_2580_MOESM2_ESM.docx]

**Supplementary Tables**

**Table S1. Antibodies used in the experiments.**

| **Antigen** | **Supplier** | **Catalog #** | **Application** | **RRID** |
| --- | --- | --- | --- | --- |
| ERα | Proteintech | 21244-1-AP | WB: 1:1000 | AB_11042600 |
| CyclinD1 | Cell Signaling  Technology | 55506S | WB: 1:1000 | AB_2827374 |
| CyclinB1 | Cell Signaling  Technology | 12231 | WB: 1:1000 | AB_2783553 |
| CDK6 | Cell Signaling  Technology | 13331 | WB: 1:1000 | AB_2721897 |
| CDK4 | Cell Signaling  Technology | 12790 | WB: 1:1000 | AB_2631166 |
| β-actin | Cell Signaling  Technology | 3700 | WB: 1:1000 | AB_2242334 |
| Beclin1 | Proteintech | 11306-1-AP | WB: 1:1000 | AB_2259061 |
| p62 | Cell Signaling  Technology | 5114S | WB: 1:1000 | AB_10624872 |
| LC3B | Cell Signaling  Technology | 2775S | WB: 1:1000 | AB_915950 |
| CDK2 | Cell Signaling  Technology | 2546S | WB: 1:1000 | AB_2276129 |
| p21 | Proteintech | 10355-1-AP | WB: 1:1000 | AB_2077682 |
| p27 | Proteintech | 25614-1-AP | WB: 1:1000 | AB_2880161 |
| Oct4 | Proteintech | 60242-1-Ig | WB: 1:5000 | AB_2881364 |
| CD133 | Proteintech | 66666-1-Ig | WB: 1:2000  IHC: 1:500 | AB_2801586 |
| Nanog | Cell Signaling  Technology | 3580 | WB: 1:1000 | AB_2150399 |
| ALDH1A1 | Proteintech | 15910-1-AP | WB: 1:1000  IHC: 1:100  IF: 1:50 | AB_2305276 |
| CD44 | Proteintech | 15675-1-AP | WB: 1:2000  IHC: 1:100  IF: 1:50 | AB_2076198 |
| CD44 | BD Biosciences | 555479 | 20µl for 10^6^ cells | AB_395871 |
| CD24 | BD Biosciences | 555427 | 20µl for 10^6^ cells | AB_395821 |
| PGRMC1 | Proteintech | 12990-1-AP | WB: 1:1000  IF: 1:50 | AB_2164342 |
| USP14 | Proteintech | 14517-1-AP | WB: 1:1000  IF: 1:50 | AB_2257124 |
| Myc | Cell Signaling  Technology | 2278S  2276S | WB: 1:1000  IP: 1:50 | AB_490778  AB_331783 |
| Flag | Invitrogen | PA1-984B | WB: 1:500  IP: 1:50 | AB_347227 |
| Flag | Cell Signaling  Technology | 8146S | IP: 1:50 | AB_10950495 |
| HA | Cell Signaling  Technology | 3724 | WB: 1:1000 | AB_1549585 |
| c-Myc | Proteintech | 10828-1-AP | WB: 1:2000 | AB_2148585 |
| Ki67 | Proteintech | 10197-1-AP | IF: 1:100 | AB_2118062 |
| K48-Ub | Cell Signaling  Technology | 4289S | WB: 1: 1000 | AB_10557239 |

**Table S2. Primers and probes sequences used for qRT-PCR ISH and FISH in the experiments.**

| **Name** | **Sense (5'-3’)** | **Antisense (5’-3’)** |
| --- | --- | --- |
| hsa_circ_0000745 | GCAGGGTGAGAAGCAGAAAG | GCCTGTCCGTTTAGTTGTTGT |
| hsa_circ_0001699 | ACAAAGACGGTCTGGAAAATC | TGTTCAATTCAGCTGCCAGG |
| hsa_circ_0001535 | GAAGAAAATACCCAGCACCCA | TACACACCACACTTTGCTGT |
| hsa_circ_0001355 | TCTTCAGCCTCGTTCACATTC | CGACGTCCCATTTCTTCTTGT |
| CircKIAA1617 DIV | ACTCCTCCCAAAGCAATCAAAG | CCTCCAATCCCACATAGCGA |
| CircKIAA1617 CON | CTCCTGGAAGGTCCTCTGC | GTGGGTCCATTCTGTATTTATTCT |
| β-actin DIV | CATTGCTGACAGGATGCAGAAG | GGAAGGCTGGAAAAGAGCC |
| U6 | CTCGCTTCGGCAGCACA | AACGCTTCACGAATTTGCGT |
| β-actin | CACTGTGCCCATCTACGAG | AATGTCACGCACGATTTCC |
| GAPDH | GGAGCGAGATCCCTCCAAAAT | GGCTGTTGTCATACTTCTCATGG |
| ESR1 | ATGACCATGACCCTCCACACC | TCAGACCGTGGCAGGGA |
| EIF4A3 | GGCACAGGAAAAACAGCCACCT | TGTAGTCACCGAGAGCAAGCAG |
| U2AF65 | TACGGGCTTGTCAAGTCCATCG | CTGGCAGTCAAACACAGAGGTG |
| PGRMC1 | ATGGCTGCCGAGGATGTG | ATCATTTTTCCGGGCACTCTCA |
| USP14 | GGGAAATGGCTTCAGCGCAGTA | CACCTTTCTCGGCAAACTGTGG |
| CircKIAA1617 ISH probe | ACTTCTCTTTGATTGCTTTGGGAGG | |
| CircKIAA1617 FISH probe | GTACTTCTCTTTGATTGCTTTGGGA | |

**Table S3. SiRNAs used for transfection.**

| **Name** | **Sense (5'-3’)** | **Antisense (5’-3’)** |
| --- | --- | --- |
| si-circKIAA1617-1 | CCCAAAGCAAUCAAAGAGA | UCUCUUUGAUUGCUUUGGG |
| si-circKIAA1617-2 | CAAAGCAAUCAAAGAGAAG | CUUCUCUUUGAUUGCUUUG |
| si-ESR1 | CAGGCCAAAUUCAGAUAAU | AUUAUCUGAAUUUGGCCUG |
| si-EIF4A3 | GUGGCCAUUAACUUUGUAA | UUACAAAGUUAAUGGCCAC |
| si-U2AF65 | CCUUUGACCAGAGGCGCUA | UAGCGCCUCTGGUCAAAGG |
| si-PGRMC1 | UAUCAUCACGUGGGCAAAC | GUUUGCCCACGUGAUGAUA |
| si-USP14 | CUGUGCCUGAACUCAAAGA | UCUUUGAGUUCAGGCACAG |
| si-ATGL | GCGAGAAGACGUGGAACAU | AUGUUCCACGUCUUCUCGC |
| si-NC | TTCTCCGAACGTGTCACGT | ACGTGACACGTTCGGAGAA |
