# Supplementary material for: CircKIAA1617 promotes stemness via USP14/PGRMC1-mediated autophagy and lipid metabolism reprogramming in ER-positive breast cancer
Source: Mol Cancer. 2026 Jan 31;25:55. doi: 10.1186/s12943-026-02580-2 (PMC12952063; doi:10.1186/s12943-026-02580-2)
Supplement: Supplementary file 3 — Supplementary Material 3. [file 12943_2026_2580_MOESM3_ESM.docx]

**Supplementary methods**

**Plasmids construction and transfection**

CircKIAA1617 overexpressing vector pLO5-circKIAA1617 and control plasmid pLO5-ciR were purchased from Geneseed Biotechnology Company (Guangzhou, China). The overexpression vectors of PGRMC1-Flag and USP14-Myc were also obtained via MiaoLing Plasmid Platform (Wuhan, China). The PGRMC1_res_ plasmids was generated by introducing synonymous mutation that preserve the amino acid sequence while transcripted mRNA regions unaffected by si-PGRMC1 binding. And the mutant vectors or truncation vectors were generated by utilizing KOD-Plus-Mutagenesis Kit (TOYOBO, Osaka, Janpan).

Lipofectamine 2000 (Invitrogen, CA, USA) was used in the plasmid transfection. Cells were seeded to reach 70-90% confluence at the time of transfection. All transfections were performed using Lipofectamine in Opti-MEM reduced-serum medium. DNA or si-RNAs and Lipofectamine were diluted separately in Opti-MEM and mixed at a 1:1 volume ratio; mixtures were incubated at room temperature for 5 minutes to allow complex formation and then added directly to cells without removal of the transfection complexes. Typical reagent amounts per well were: DNA 2,500 ng (6-well); Lipofectamine 2000 5.0-12.5 µL (6-well). The volume of DNA-lipid complex added per well was 250 µL (6-well). After transfection, cells were incubated at 37°C and analyzed 24-72 h later, as indicated for each assay. For transfection of siRNA, a ratio of 7.5 pmol siRNA was used. Reagent storage and general handling followed the manufacturer’s recommendations.

To establish stable cell lines, lentiviral transduction was performed. Briefly, the target plasmid was co-transfected with the packaging plasmids psPAX2 and pMD2.G into HEK-293T cells using Lipofectamine 2000. Viral supernatants were harvested 48 h post-transfection and filtered through a 0.22 μm filter. MCF7 and T47D cells were then infected with the viral supernatant for 48 h, followed by selection with puromycin for 2-3 weeks to obtain stable cells.

**High throughput circRNA-seq assay**

The sample preparation and circRNA sequencing were performed by Lc-Bio Technologies (Hangzhou, China). Significantly differentially expressed circRNAs were retained by screening for fold change R 2.0 and p < 0.05. The circRNA-seq results were deposited in GEO (GSE312147).

**High throughput RNA-seq assay**

Total RNA was extracted with RNAiso Plus (Takara, Japan) according to the manufacturer’s instructions. High-throughput sequencing was performed by Cloud-Seq Biotech (Shanghai, China) following their standard protocols. Sequencing reads were quality-checked and adapter-trimmed, then aligned to the human reference genome (GRCh38) using a splice-aware aligner. Gene-level read counts were obtained with a read-counting tool and used for downstream analysis.

Differential expression analysis was performed using the Bioconductor package edgeR. Raw counts were filtered to remove lowly expressed genes and normalized using the trimmed mean of M-values (TMM) method. Differential expression was assessed with a generalized linear model framework and empirical Bayes moderation as implemented in edge R; resulting P values were adjusted for multiple testing using the Benjamini-Hochberg false discovery rate (FDR) procedure, and genes with FDR<0.05 were considered significant. Functional enrichment (KEGG) was similarly evaluated using FDR-adjusted P values. All computational analyses were carried out in R. The RNA-seq results were deposited in GEO (GSE312149).

**RNA isolation and quantitative real-time PCR (qRT-PCR)**

The RNA-easy Isolation Reagent (Vazyme, Nanjing, China) was used to isolate total RNAs of whole cell lysates from cell lines or tissues, and the purity and concentration of RNAs were evaluated by the NanoDrop 2000 (Thermo Fisher Scientific, Waltham, USA). CDNA was generated from RNAs by the Prime Script reverse transcriptase reagent kit (Takara, Shiga, Japan). Subsequently, qRT-PCR was performed to examine the indicated RNA levels via SYBR Premix Ex Taq II (Takara) and the Light Cycler 480 II Real-Time PCR System (Roche, Switzerland). The β-actin was served as the endogenous control. Table S2 contains the primers utilized in this investigation.

**RNase R and actinomycin D treatment**

RNase was used to verify the stability of the circKIAA1617 and KIAA1617 mRNA. Isolated from MCF7 or T47D cells, RNAs were divided into two parts, one part was treated with RNase R and the other one for Mock. Total RNA (2μg) was incubated with 3 U/mg of RNase R for 30 min at 37℃, and the enzyme would be inactivated at 70℃ for 10 min. In order to test the half-life of circKIAA1617 and KIAA1617 mRNA, cells were treated with 5 μg/ml Actinomycin D (ActD, Sigma, USA) for 0, 4, 8, 12 and 24 h before the extraction of RNA. Subsequently, the expression levels of circKIAA1617 and KIAA1617 mRNA were detected using qRT-PCR.

**Cell proliferation assay**

MTT (3- (4,5-dimethyl-2-thiazolyl)-2,5-diphenyl-2H-tetrazolium bromide) assays were used to monitor the cell proliferation rate. 1500 MCF7 or T47D cells per well were seeded into 96-well cell culture plates for the proliferation rate experiment. After cell attachment, 20μl of MTT (5 mg/ml) was added to each wells culturing cells. Incubation for 4 to 6 hours at 37 ℃, the supernatants were aspirated and the formazan was dissolved by 100μl DMSO per well. Finally, the absorbance values were measured by a microplate reader (Bio-Rad, CA, USA) at 490 nm.

**Flat plate colony formation assay**

Colony formation assay was also performed to assess the proliferation of cells. 1500 ER-positive BC cells after transfection were inoculated into a well of the 6 well cell culture plates. After incubation for around three weeks, those cells would undergo three rounds of PBS washing, a 15-minute methanol fixation, and a 15-minute 0.2% crystal violet staining period in sequence before being rinsed, photographed, and tallied.

**EdU assay**

Cell proliferation abilities were assessed by EdU Proliferation Kit (RiboBio Guangzhou, China). 1×10^4^ ER-positive BC cells transfected with vectors or siRNAs were seeded into a well of the 96-well cell culture plates to be cultured for 48 hours. Following a two-hour incubation period with 50 mM EdU, cells were fixed with 4% paraformaldehyde (PFA) and washed with PBS before being dyed with Apollo Dye Solution. Hoechst was employed to stain the cell nucleus. Images were obtained with a ZEISS microscope (ZEISS, Oberkochen, Germany).

**Flow cytometry (FCM)**

MCF7 and T47D cells were collected and washed three times with PBS. For cell cycle assay, cells were stained by cell cycle staining buffer (Multi Sciences, Hangzhou, China) for 30 min in the dark. For cell stemness assay, PE-CD44 (Invitrogen, USA) and FITC-CD24 (Invitrogen, USA) were used for detecting the ratio of CD44+CD24-cells. Stained cells were washed three times to remove redundant dye or antibodies, and examined by a FACSCalibur flow cytometer (BD Biosciences, CA, USA).

**Fluorescence in situ hybridization (FISH)**

Cy3-labeled probe targeting the splicing junction of circKIAA1617 was designed. The targeted sequence of which was shown in Table S2. FISH assay was performed with the RNA FISH Kit according to the manufacturer’s protocols (GenePharma, Jiangsu, China). The subcellular location of circKIAA1617 was observed and photographed under a fluorescence microscope (Leica, Wetzlar, Germany).

**In situ hybridization (ISH)**

The ISH assay was conducted using the Enhanced Sensitive ISH Detection kit I (BOSTER, China) following the manufacturer’s instruction. The specific digoxin-labeled probe of circKIAA1617 probe was designed and synthesized by GenePharma (Shanghai, China), and the sequence was shown in Table S2. Briefly, the tissue sections were fixed with 4% paraformaldehyde for 15 min, blocked by 30% H2O2+ methanol (1:50) for 30 min, and digested by pepsin for 15–30 min in room temperature. Next, the sections were treated with prehybridization solution in 37 °C for four hours and incubated with the specific digoxin-labeled circKIAA1617 probe in 37 °C overnight. The next day, the sections were incubated with blocking reagent, biotinylation rat anti digoxin, SABC, and biotinylation peroxidase successively. The expression of circKIAA1617 was visualized by DAB staining, and nucleus was stained by hematoxylin (Solarbio, China). Images were captured using a light microscope.

**Immunofluorescent (IF)**

Approximate 6×10^4^ cells were seeded onto glass slides placed in 24 well plates and cultured for 24 hours at 37 ℃. The next day, the cells were washed three times with PBS and fixed with 4% paraformaldehyde for 15 min. Then cells were permeabilized with 0.3% Triton X-100 for 25 min and blocked with 10% goat serum for an hour, followed by incubation with indicated primary antibodies overnight at 4 ℃. The next day, the cells were incubated with fluorescent secondary antibodies (ZSGB-BIO, Beijing, China) at room temperature in dark for one hour. Following three washes with PBS, the nucleus was stained with DAPI. Images were acquired with a fluorescence microscope (ZEISS, Oberkochen, Germany).

**Patient-derived organoid (PDO) culture, treatment and transfection.**

Fresh specimens of breast cancer tissues were cut into 1mm^3^ size fragments on ice and then digested for two hours at 37 °C with gentle shake. The digestion medium used was DMEM/F12 (Macgene, China), which contained 1% BSA, ITS-G (BasalMedia Technologies Company, China), Y-27632 (5μM, MCE, USA), Primocin (Invitrogen, USA), HEPES (10mM, Thermo, USA), hyaluronidase (1000U/mL, Sigma-Aldrich, USA), and collagenase I and III (Worthington, Italy). The digestion mixture was then centrifuged at 300g for 5 min at 4 °C after being filtered through a 100μm filter strainer. Digestion termination solution (DMEM/F12 mixed with 0.1% BSA and primocin) was used to resuspension the cell pellets. Following centrifugation, the erythrocytes were lysed using TAC buffer, and the sediment was twice cleaned with the digestion termination solution. The growth factor-reduced Matrigel (R&D Systems, USA) would be diluted 1:3 in organoid culture media and used to resuscitate the cell pellets. Subsequently, the organoid suspension was seeded into a 48-well plate and allowed to solidify at 37 °C in an incubator. Each well that had previously been seeded with organoid suspension received 300 μl of organoid culture media after an hour. The culture medium of organoid was composed of DMEM/F12 medium supplemented with 0.5µg/ml R-spondin-1 (BioLegend, USA), 0.5 µg/ml Neuregulin 1 (PeproTech, USA), 5ng/ml human recombinant FGF7 (PeproTech, USA), 5ng/ml human EGF (PeproTech, USA), 0.1µg/mL human Noggin (PeproTech, USA), 20ng/ml human FGF10 (PeproTech, USA), 500nM A83-01 (Tocris Bioscience, USA), 5µM Y-27632 (Sigma-Aldrich, USA), 500nM SB202190 (Sigma-Aldrich, USA), 1.25mM N-acetyl-L-cysteine (Sigma-Aldrich, USA), 5mM Nicotinamide (Sigma-Aldrich, USA), 1×Primocin (Invitrogen, USA), 1×GlutaMax (Invitrogen, USA), 1×HEPES (Thermo, USA), and 1×B27 (Gibco, USA). Every three days, fresh medium was added, and organoids were passed through using TrypLE (Gibco, USA).

CircKIAA1617-overexpressing or control lentiviral were employed to infect these cells 48 hours after seeding. PDOs were dissociated in ice-cold medium, centrifuged at 850 rpm, and further broken down by gentle pipetting before special trypsin digestion for 5 min at 37 °C. After quenching with DMEM containing 5% FBS and antibiotics, cells were centrifuged and resuspended in lentivirus supplemented with Polybrene, then spin-infected in 48-well organoid plates. Following a 1-6 h incubation at 37 °C, cells were washed, pelleted, and embedded in Matrigel (50 µL per well of a 24-well plate). Antibiotic selection was initiated 2 days later using empirically determined optimal concentrations.

Silencing circKIAA1617 in PDOs was depend on electroporation which was performed using the Amaxa 4D-Nucleofector system (P3 Primary Cell 4D-Nucleofector™ X Kit). Briefly, PDOs were collected, resuspended in nucleofetion reagent (Nucleofector™ kits for human stem cells, Lonza) containing siRNA and transferred into nucleofection vials. Nucleofection was performed according to the manufacturer’s protocol. Immediately after the electroporation, PDOs were resuspended in prewarmed culture medium (see manufacturer’s recommended post-nucleofection volumes) and plated into pre-equilibrated culture plates. PDOs were incubated at 37 °C/5% CO₂ and analyzed for expression or knockdown 4-48 h post-nucleofection.

Images of PDOs were captured using a light microscope.

**MDC staining assay**

MCF7 and T47D cells were grown in 24-well plates and cultivated at 37°C with 5% CO_2_. Cells were stained with MDC according to the protocol for 45 min. Under a fluorescent microscope, the cells were immediately observed and photographed.

**RNA pulldown assay, silver staining, and mass spectrometry (MS) analysis**

The sense probes and anti-sense probes of circKIAA1617 were biotinylated and incubated with lysates of ER-positive BC cells for RNA pull-down assays. Then, RNA pulldown assay was proceeded by using a Pierce Magnetic RNA-Protein Pull down Kit (Thermo Fisher Scientific, USA) to obtain RNA-binding proteins (RBPs) according to the manufacturer’s protocol, and the candidate proteins were further detected by western blot. Isolated proteins in SDS-PAGE were visualized by the Fast Silver Staining Kit (Beyotime, China). Then the gel strips were cut off and sent to Qinglian Biotech company (Beijing, China) for protein identification by MS analysis.

**RNA immunoprecipitation (RIP) assay**

RIP assays were used to confirm the binding between circKIAA1617 and the indicated proteins by magnetic RIP RNA-binding protein immunoprecipitation kit (Millipore, USA) according to the manufacturer’s instructions. Briefly, approximate 1×10^7^ cells lysed in RIP lysis buffer were stored at -80 °C. Specific antibodies were incubated with magnetic beads with rotation for 30 min at room temperature. The supernatant of lysed cells was collected and immunoprecipitated with magnetic beads conjugated of corresponding primary antibodies at 4℃ overnight. The co-precipitated RNA was washed, purified utilizing RNA extraction reagent, and detected by qRT-PCR.

**Chromatin immunoprecipitation (ChIP)**

ChIP assays were performed using the EZ-ChIP Kit (Millipore, 17-371) in accordance with the manufacturer’s instructions. In brief, 1% formaldehyde was used to cross-link MCF7 and T47D cells for 10 minutes at room temperature. The cells were then quenched in 0.125M glycine for 5 minutes. After the cells were extracted in SDS lysis solution, sonication was used to break the DNA into fragments ranging from 200 to 1000 bp. Following pre-clearing, the samples were incubated for one night with either the primary or control IgG antibodies, and then for two hours at 4°C with protein-A/G agarose beads (Millipore, IP05). Following washing, the chromatin was eluted using TES buffer (1% SDS, 1 mM EDTA, 10 mM Tris-Cl, pH 8.0), and proteinase K (Millipore, 20-298) was then incubated at 55 °C for 45 minutes and then at 65 °C for an overnight period. After the DNA was precipitated with ethanol, extracted using phenol-chloroform, and subjected to qPCR analysis. Comparing fold enrichment to IgG was done.

**Luciferase reporter assay**

The full length of the circKIAA1617 promoter were amplified by PCR, while their mutant fragments were obtained by overlap extension PCR. The PCR fragments were inserted into pGL4.26 vectors (Invitrogen, USA) respectively to construct luciferase reporter plasmids. Cells were co-transfected with the wild type or mutant luciferase reporter plasmids along with PRL-TK vector include Renilla served as substrate to calculate the relative luciferase activity. After 48 hours, the luciferase activity was measured by the Dual-Luciferase® Reporter Assay System (Promega, USA).

**Protein isolation and western blot**

Cells lysis buffer (Beyotime, Shanghai, China) with 1%PMSF (Beyotime, Shanghai, China) and 1%NaF was used to lyse cells for total proteins. The total proteins were quantified by BCA Protein Assay Kit (Millipore, USA). Then, the proteins were isolated by 10% SDS-PAGE and transferred onto 0.22 μm PVDF membranes (Millipore, USA). After blocked by 5% non-fat milk for an hour, the membranes were incubated with corresponding primary antibodies overnight at 4 °C and indicated secondary antibodies of western blot (Cell Signaling Technology, Boston, USA) for two hours at room temperature. Finally, the protein levels could be detected by chemiluminescence (Millipore, Billerica, MA, USA). β-actin was served as endogenous control. The antibodies used in this study were supplied as Table S1. The grayscale intensity of each band was quantified using Image J, and the value was normalized to the intensity of the corresponding β-actin band to obtain the relative densitometry for each blot image.

**Co-immunoprecipitation (co-IP).**

After at least 30 minutes of lysis at 4°C in IP lysis buffer (Beyotime, Shanghai, China), the cells were centrifuged for 30 minutes. Next, 500μg of protein sample supernatant was removed, and 5μg of immunoprecipitating primary antibody or control IgG was added separately. This was done for two hours at 4°C with moderate rotation. Following an overnight (4°C) incubation with 40μl protein-A/G agarose (Santa Cruz Biotechnology, sc-2003), the beads were resuspended in 40μl 2×SDS sample buffer and heated for 10 minutes at 95°C. This process was repeated at least four times using IP lysis buffer at 300g. The samples underwent western blot analysis.

**Cytosolic/nuclear fraction assay**

The PARIS™ Kit (Invitrogen, USA) was used to isolated the cytosolic and nuclear RNAs according to the manufacturer’s instructions.

**Immunohistochemistry (IHC)**

Sections of 4μm were cut from paraffin-embedded tumor samples that were taken from people or mice, deparaffinized in xylene, and then rehydrated with gradient alcohol. Following antigen retrieval and endogenous peroxidase block, the slices were treated with certain primary antibodies for a whole night at 4 °C. Subsequently, they were incubated with secondary antibodies coFL1njugated with HRP. DAB solution was used to view the sections, and hematoxylin was used as a counterstain. Images were taken using a light microscope. Table S1 contained a list of the antibodies.

**BODYPI staining**

Intracellular lipid accumulation was quantified using BODIPY 493/503 (Invitrogen, Carlsbad, USA), a fluorescent dye that binds to neutral lipids. Cells were seeded onto glass slides placed in 24 well plates and cultured for 24 hours at 37 ℃. On the following day, cells were washed with PBS for three times and stained with 1μM BODIPY solution for 30 min. The fluorescence was measured with a FACSCalibur flow cytometer (BD Biosciences, CA, USA). In addition, images of intracellular lipid droplets were acquired with a fluorescence microscope (ZEISS, Oberkochen, Germany).

**Oil red staining**

MCF7 and T47D cells were inoculated on cell slides for 24 hours. Cells were then fixed with 4% paraformaldehyde for 30 minutes and rinsed twice with PBS. After staining with 0.3% Oil Red O (Solarbio Technology, Beijing, China) for 50 minutes, the cells were washed twice with 60% isopropyl alcohol. Cells were stained with hematoxylin for 30 s and immediately washed five times with deionized water. The slides were fixed with glycerol/water (1:1) and the formation of lipid droplets in the cells was observed using a light microscope.

**Ad-mCherry-GFP-LC3B**

The cells were inoculated on cell slides for 24 hours. After growing to a suitable density, Ad-mCherry-GFP-LC3B was added (Beyotime, Shanghai, China), resulting in a final concentration of 10−11 vp/mL. After stimulating for 48 h, the numbers of mCherry and GFP fluorescence points were directly observed under a fluorescence microscope (ZEISS, Oberkochen, Germany).

**Lyso-Tracker staining**

Indicated MCF7 and T47D cells were seeded into cell slides in 24 well plates and cultured for 24 hours. The cells were stained with 50 nM Lyso-Tracker Green (Beyotime, Shanghai, China) working solution at 37 ◦ C for 15 min. Cell nuclei were stained with DAPI (Beyotime, Shanghai, China). Additionally, the co-localization of lysosomes and lipid droplets was analyzed using ImageJ software.

**Sphere formation assay**

MCF7 and T47D cells were harvested and re-suspended in sphere formation medium, which was composed of DMEM supplemented with 1×B27, 20 ng/mL fibroblast growth factor-basic and 20 ng/mL epidermal growth factor (Thermo Fisher Scientific, Waltham, MA, USA). MCF7 and T47D cells were plated in a 48-well Clear Flat Bottom Ultra-Low Attachment Microplate (CORNING, Corning, NY, USA) at a density of 1000 cells per well. After 14 days, the spheres were recorded using a light microscope.
