# Supplementary material for: CircKIAA1617 promotes stemness via USP14/PGRMC1-mediated autophagy and lipid metabolism reprogramming in ER-positive breast cancer
Source: Mol Cancer. 2026 Jan 31;25:55. doi: 10.1186/s12943-026-02580-2 (PMC12952063; doi:10.1186/s12943-026-02580-2)

Figure 1. D

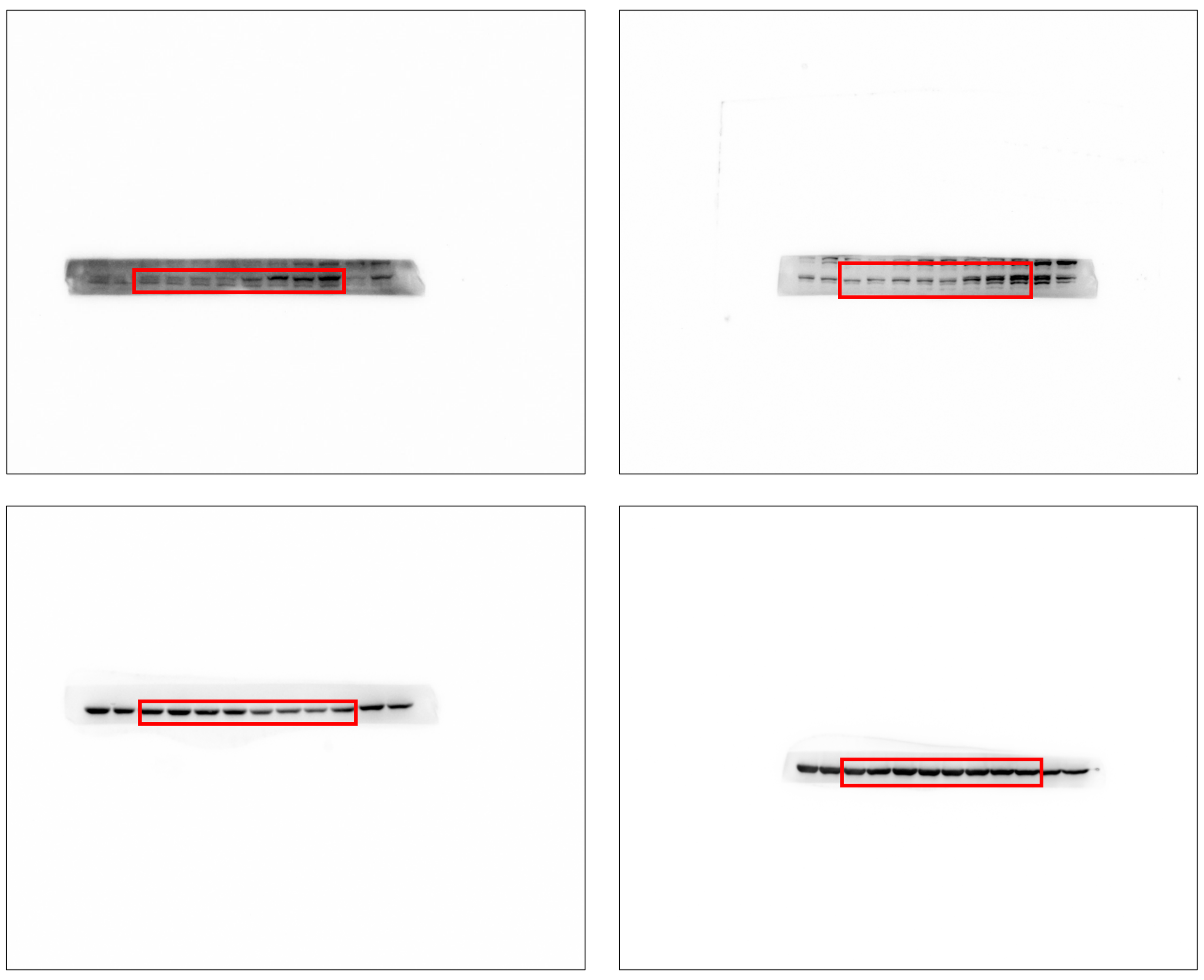

Figure 1. E

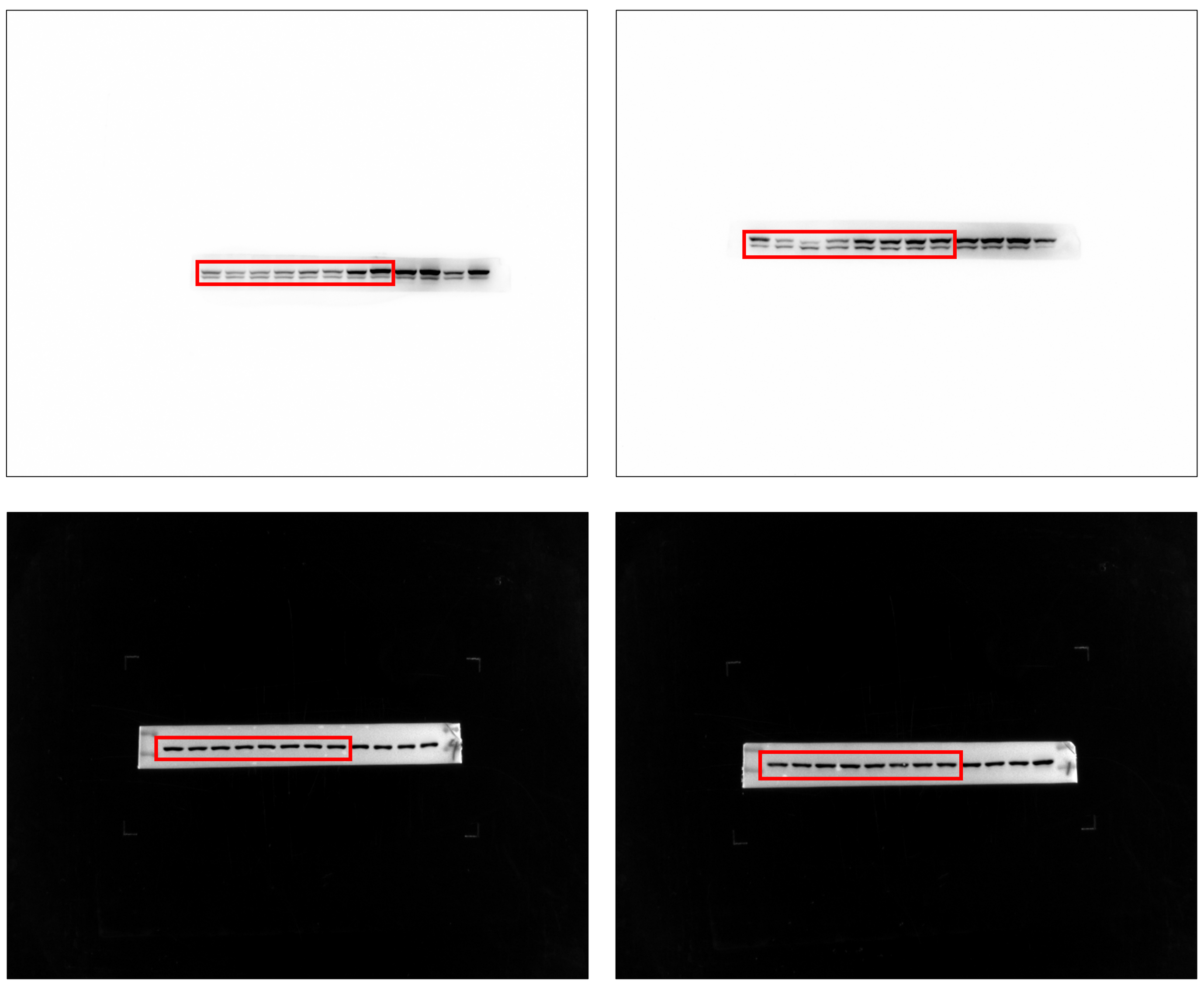

Figure 1. K

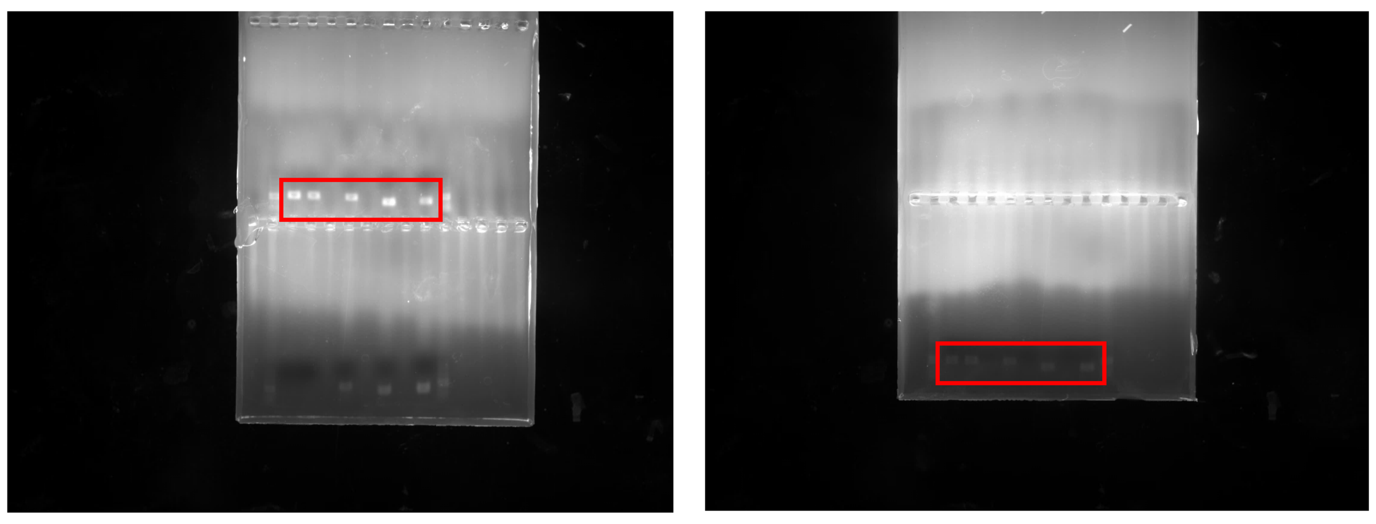

Figure 1. L

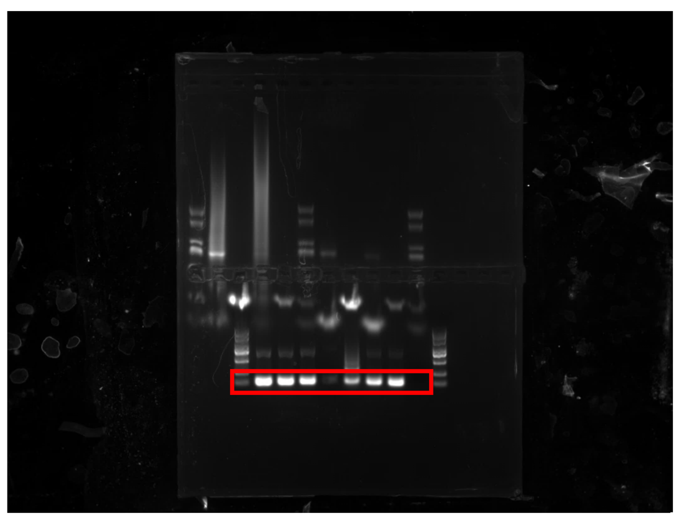

Figure 2. C

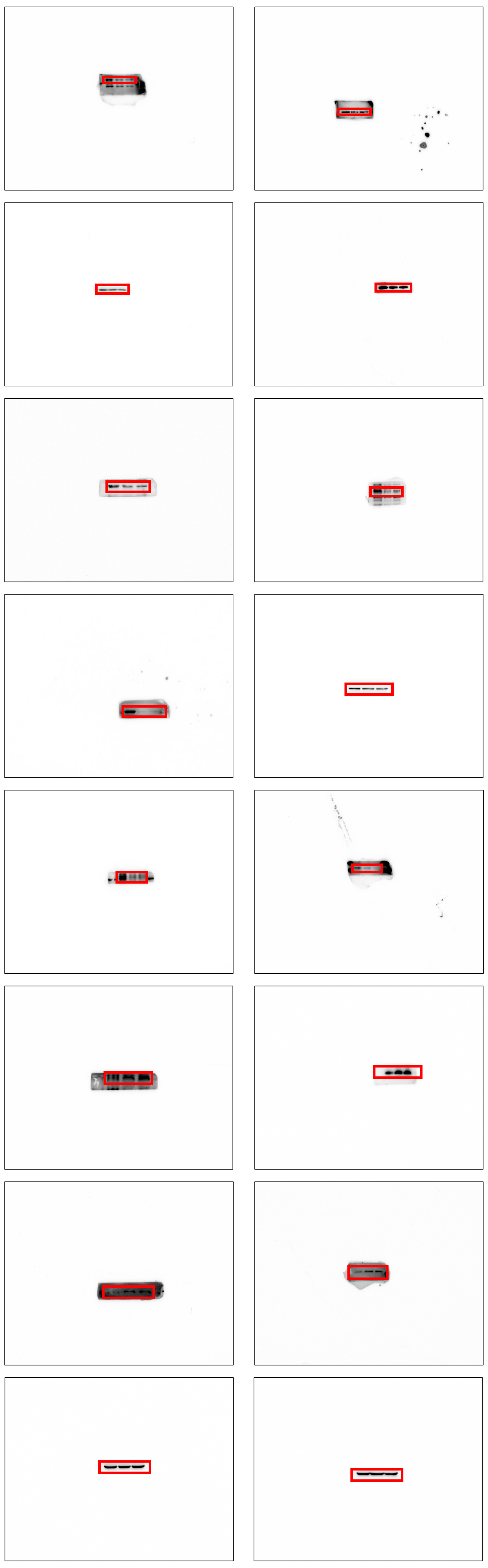

Figure 3. G

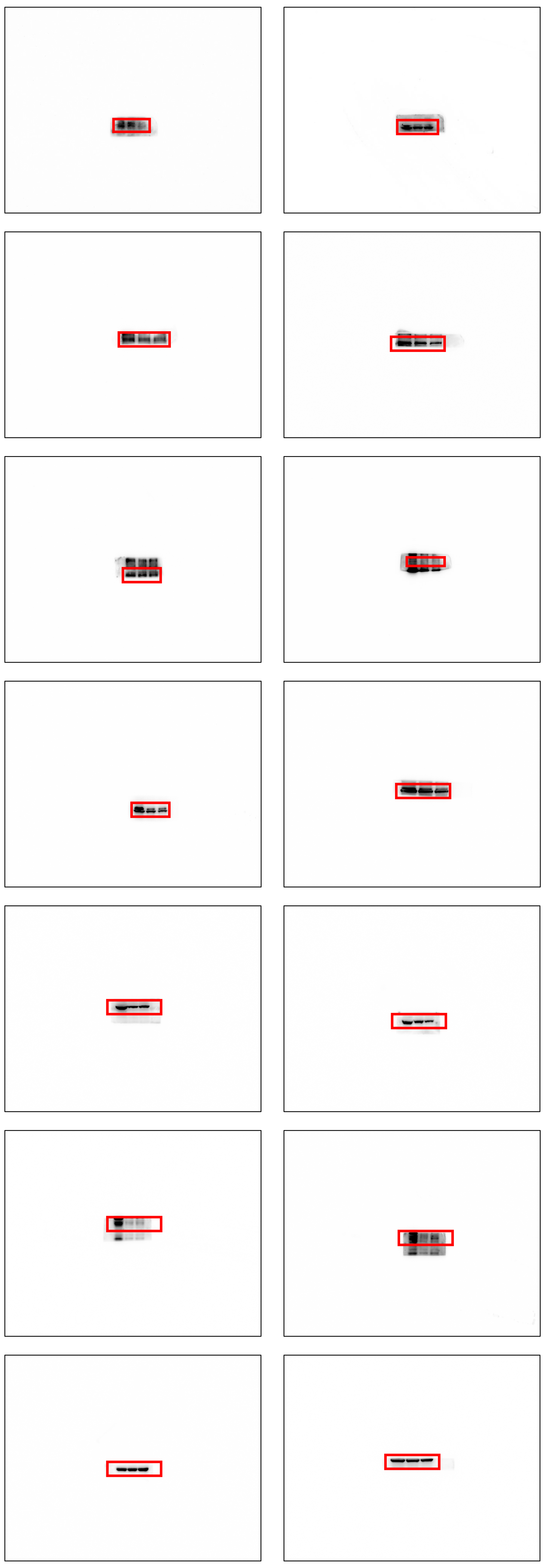

Figure 2. L

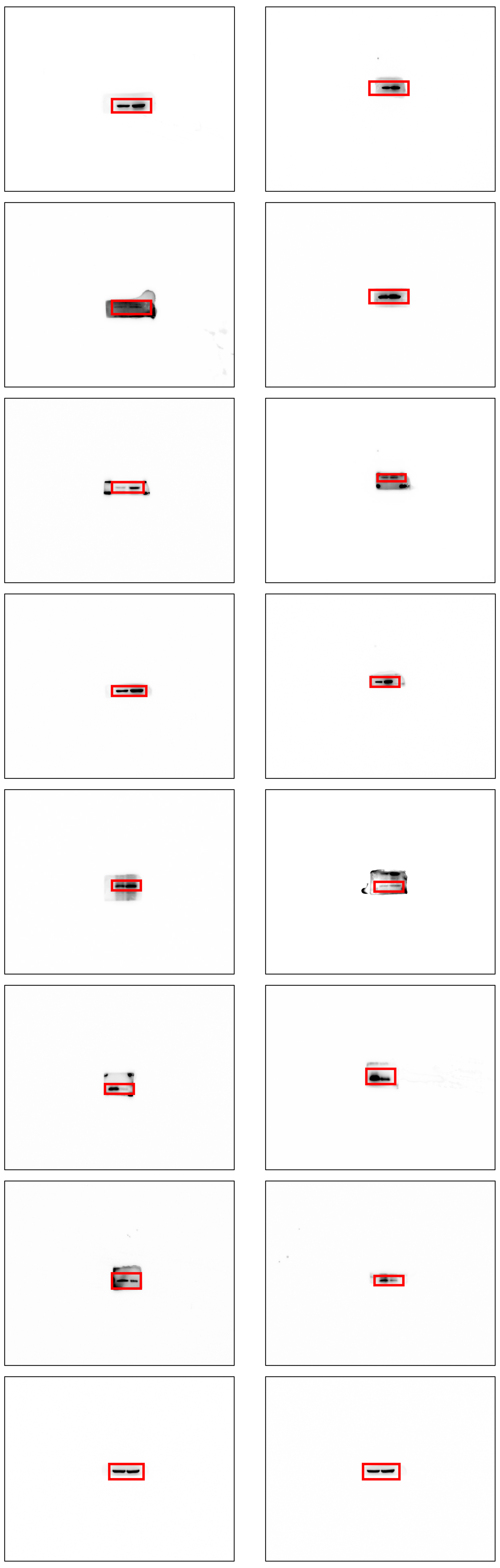

Figure 3. D

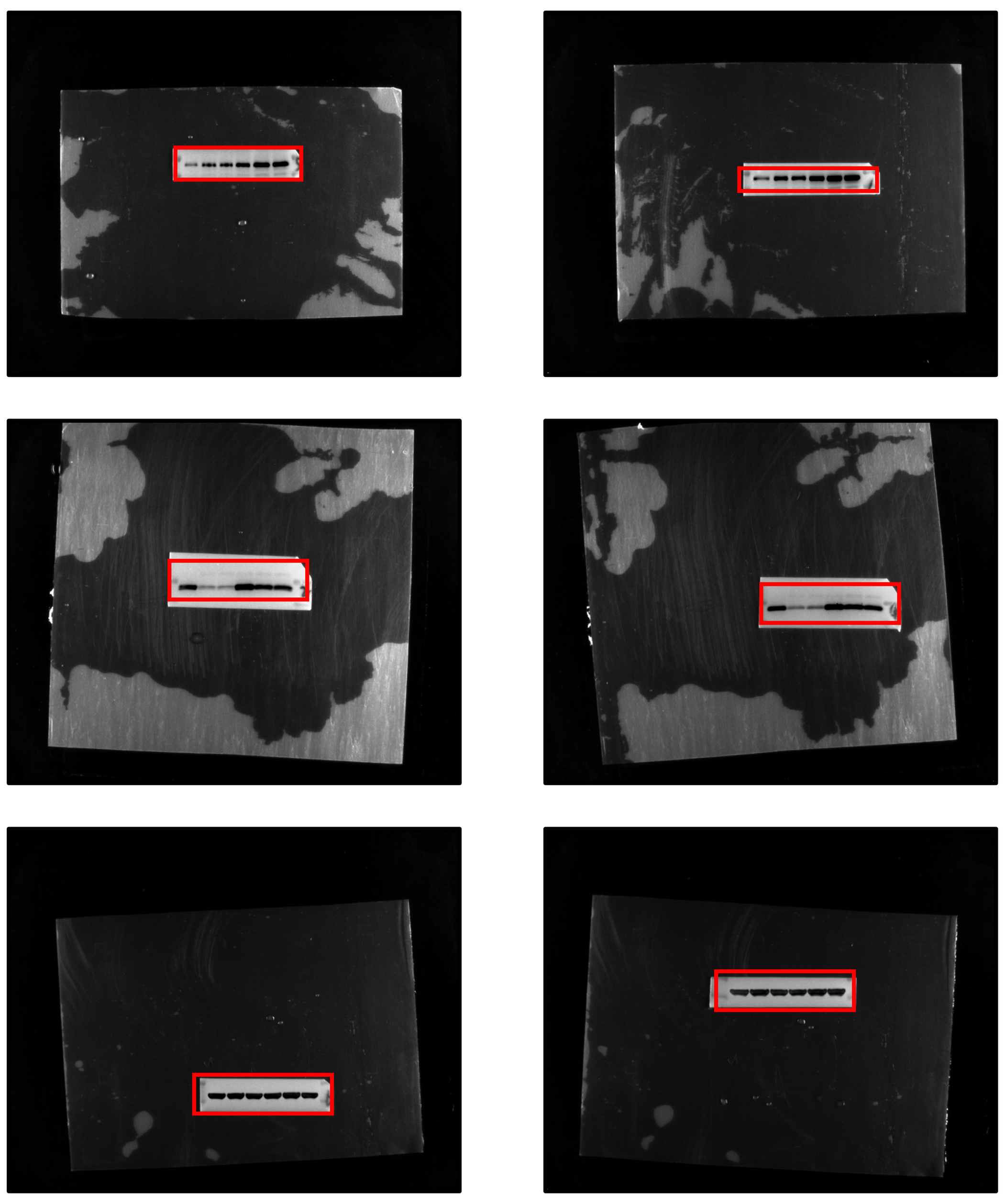

Figure 3. G

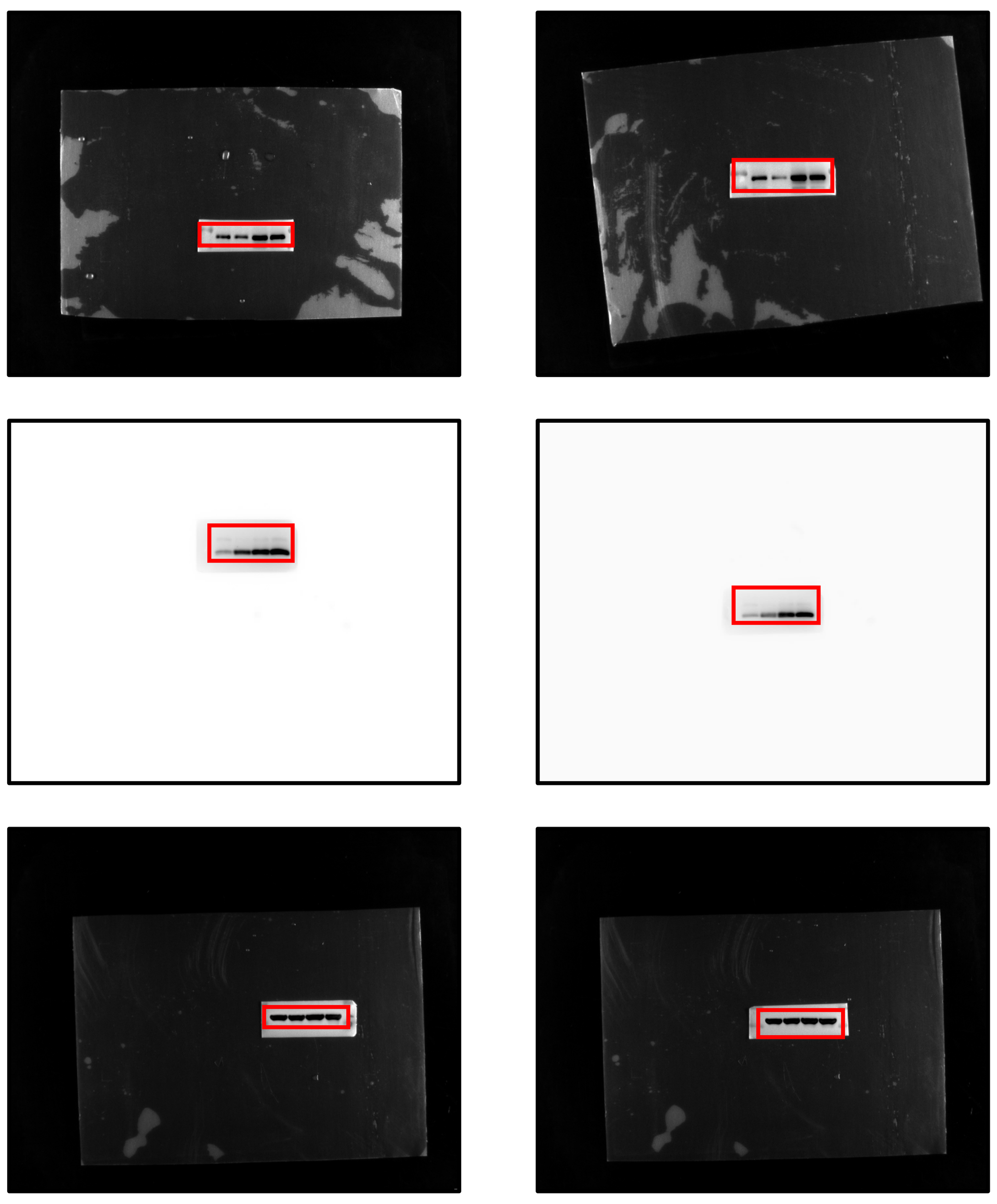

Figure 3. K

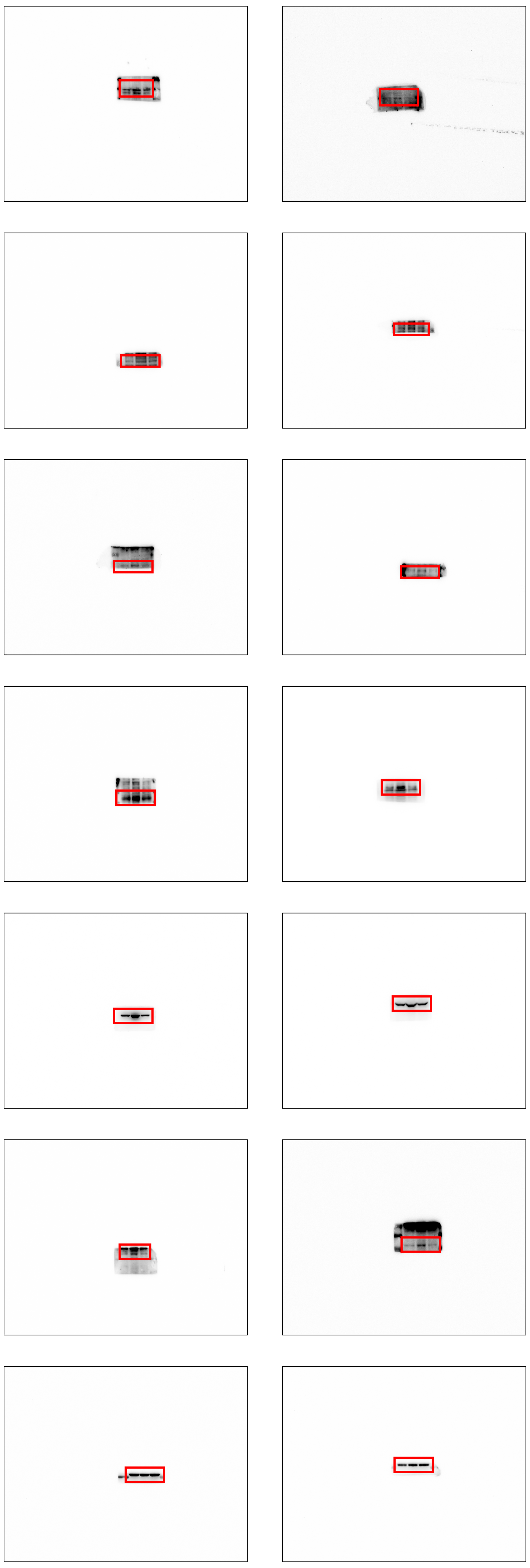

Figure 4. G

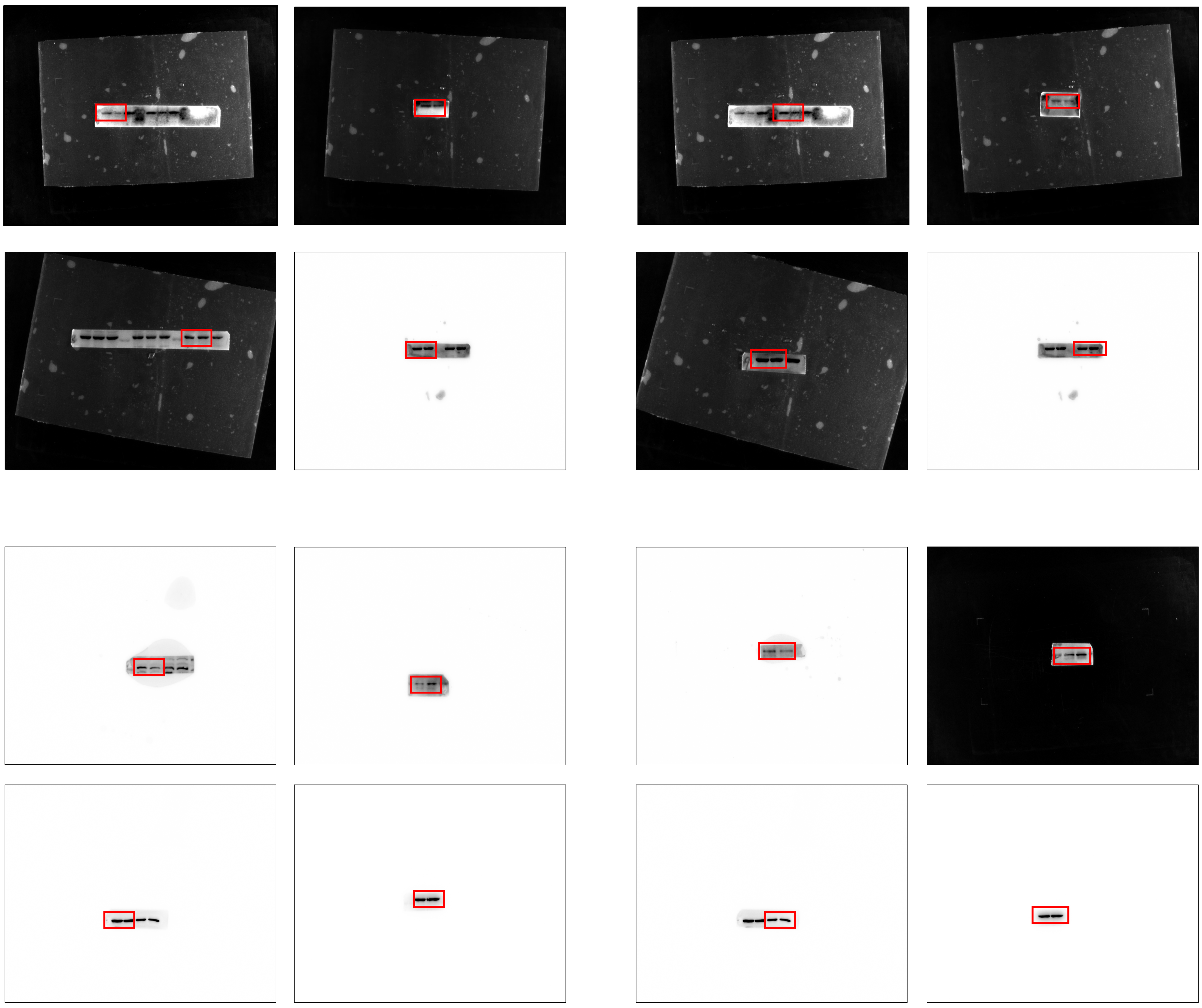

Figure 5. E

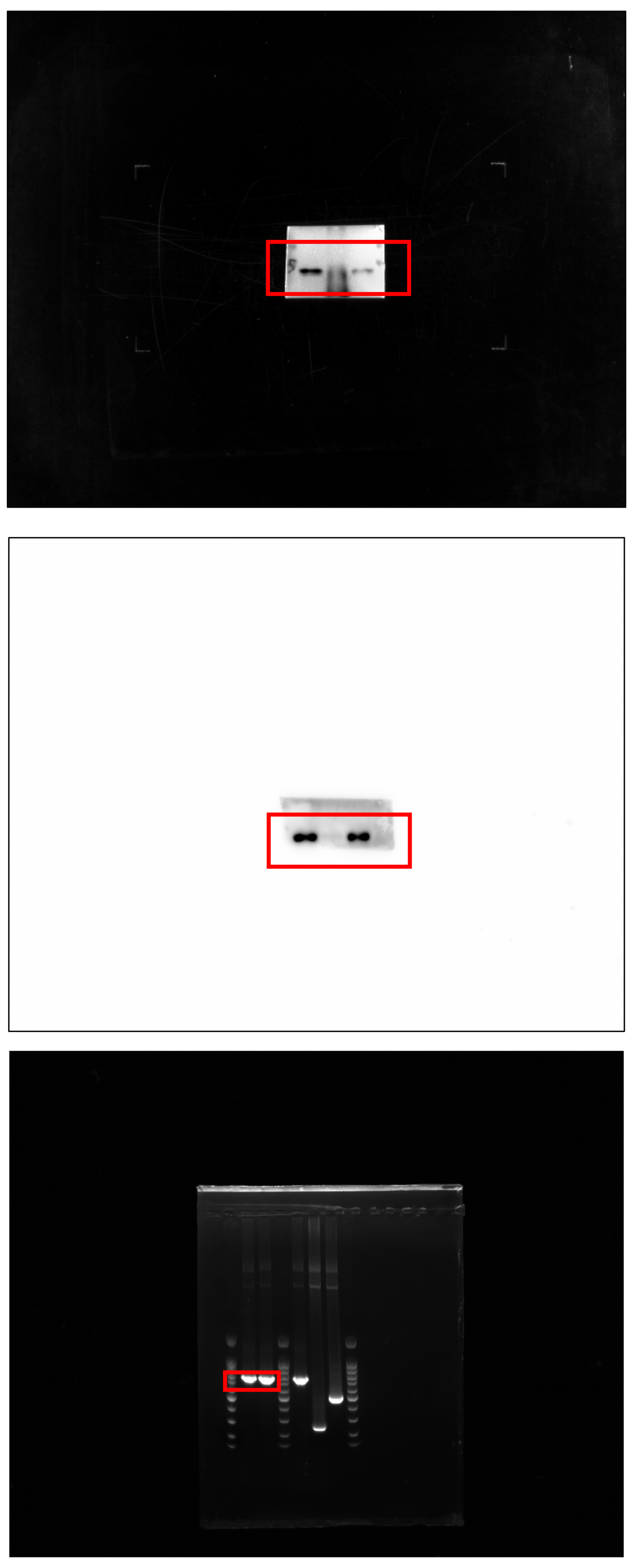

Figure 5. H

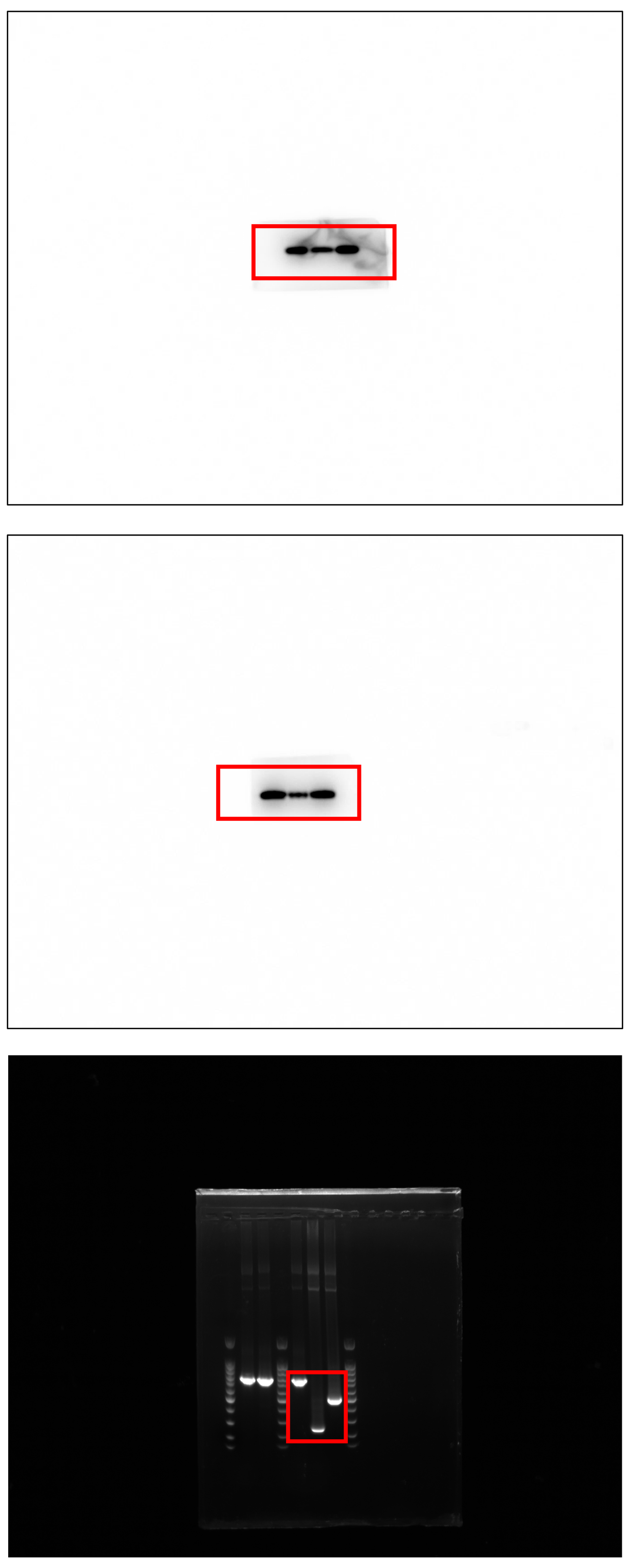

Figure 5. J

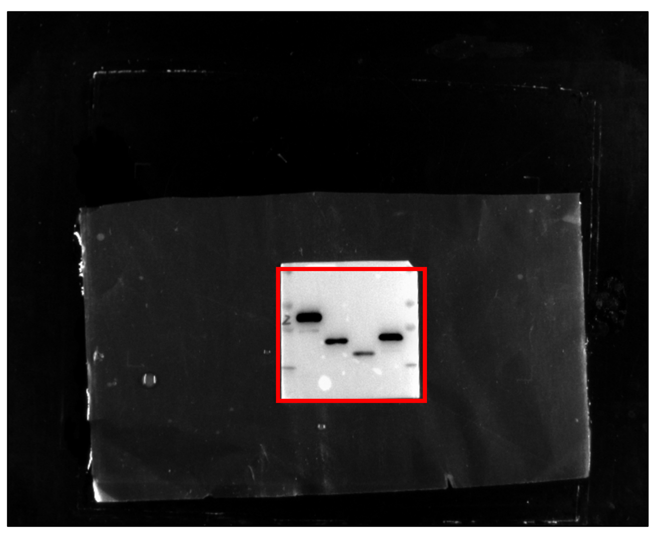

Figure 5. M

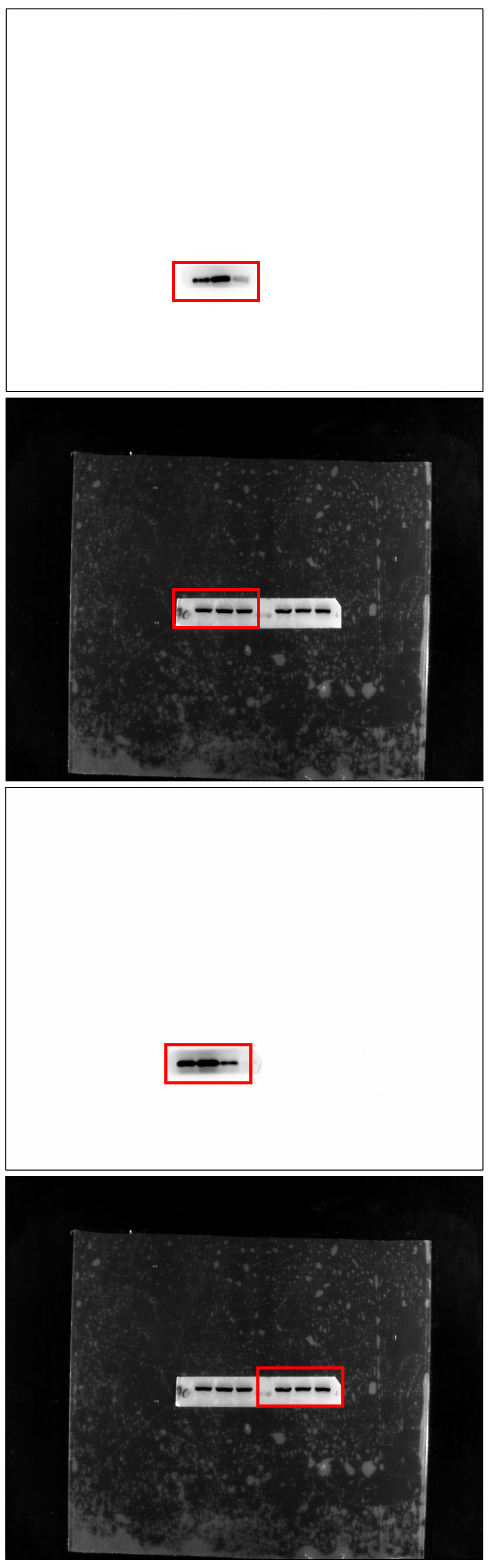

Figure 5. P

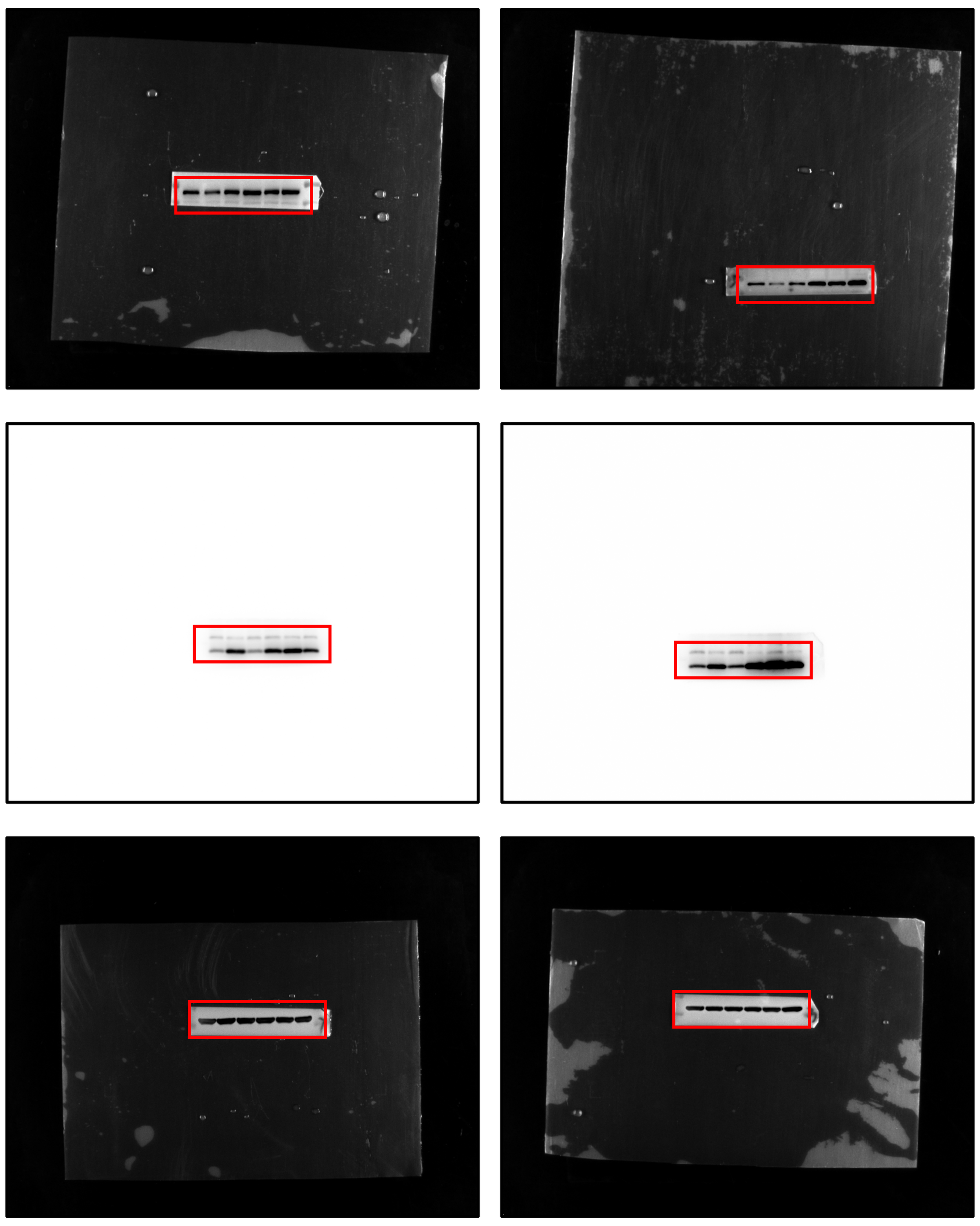

Figure 6. D

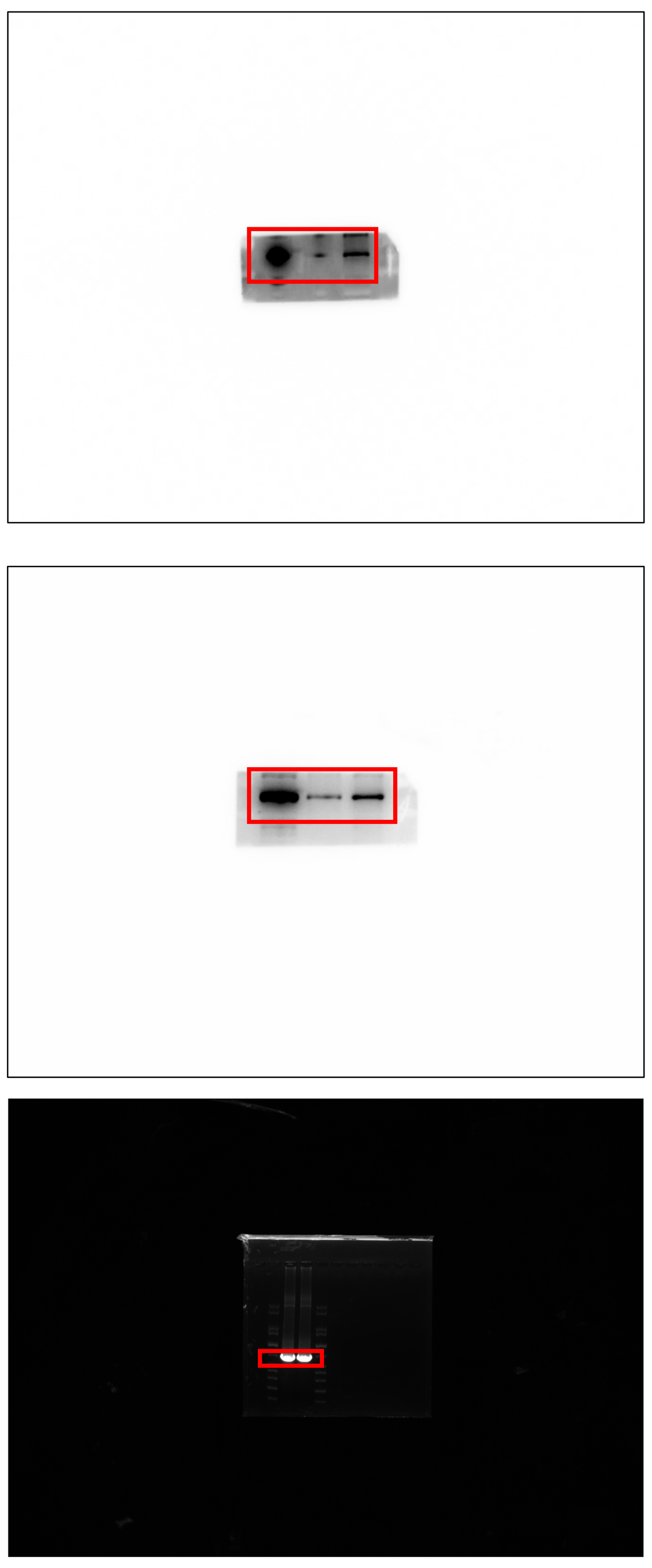

Figure 6. G

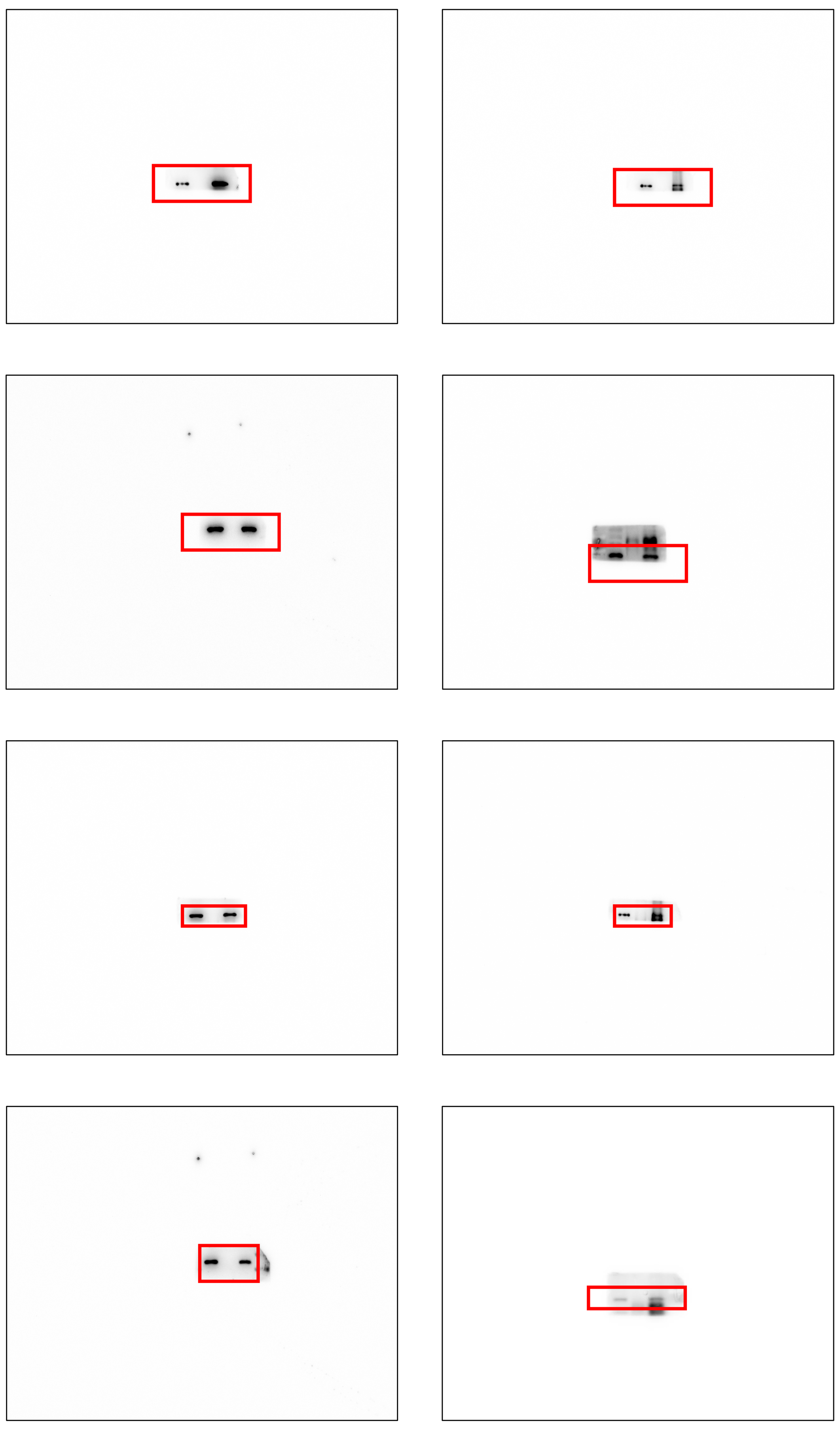

Figure 6. H

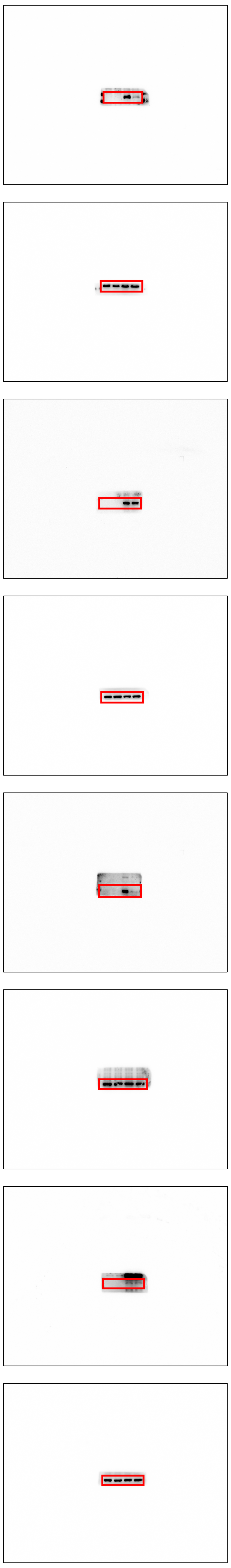

Figure 6. I

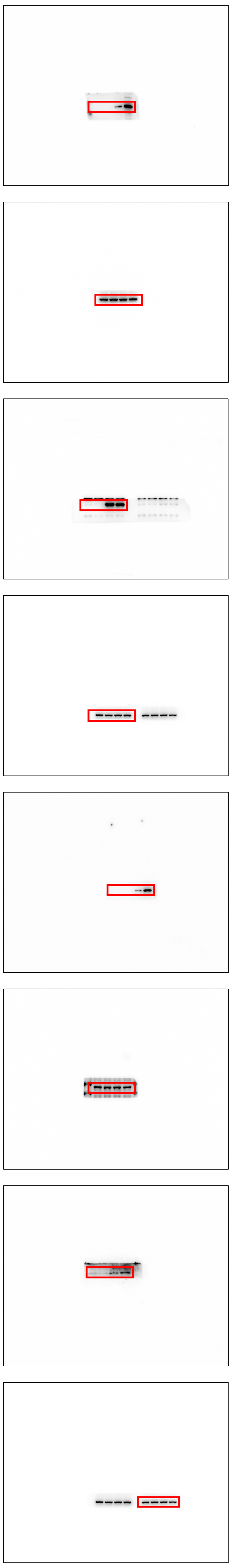

Figure 6. J

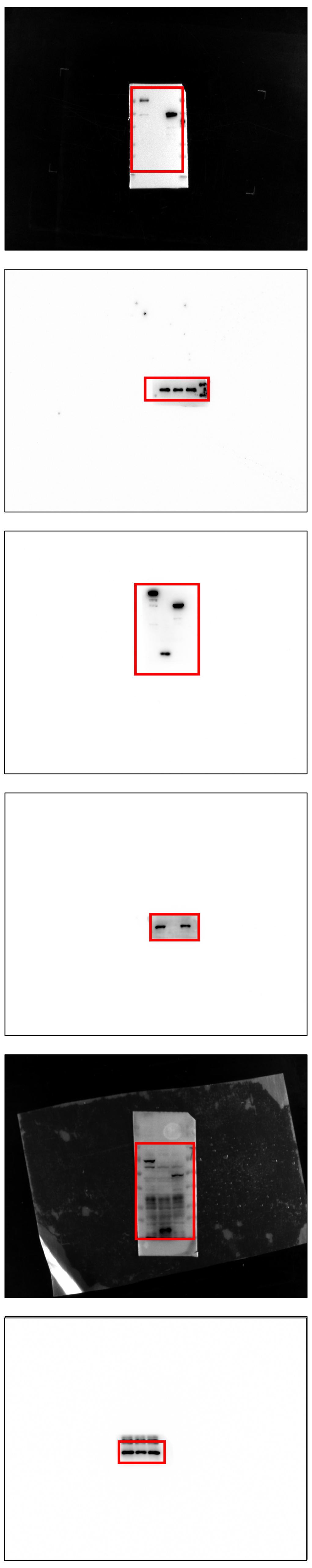

Figure 6. K

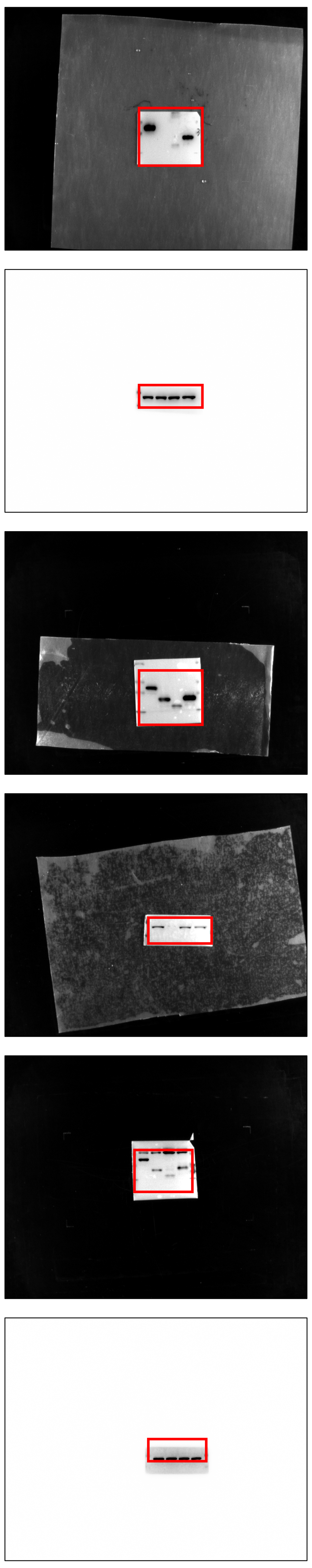

Figure 6. M

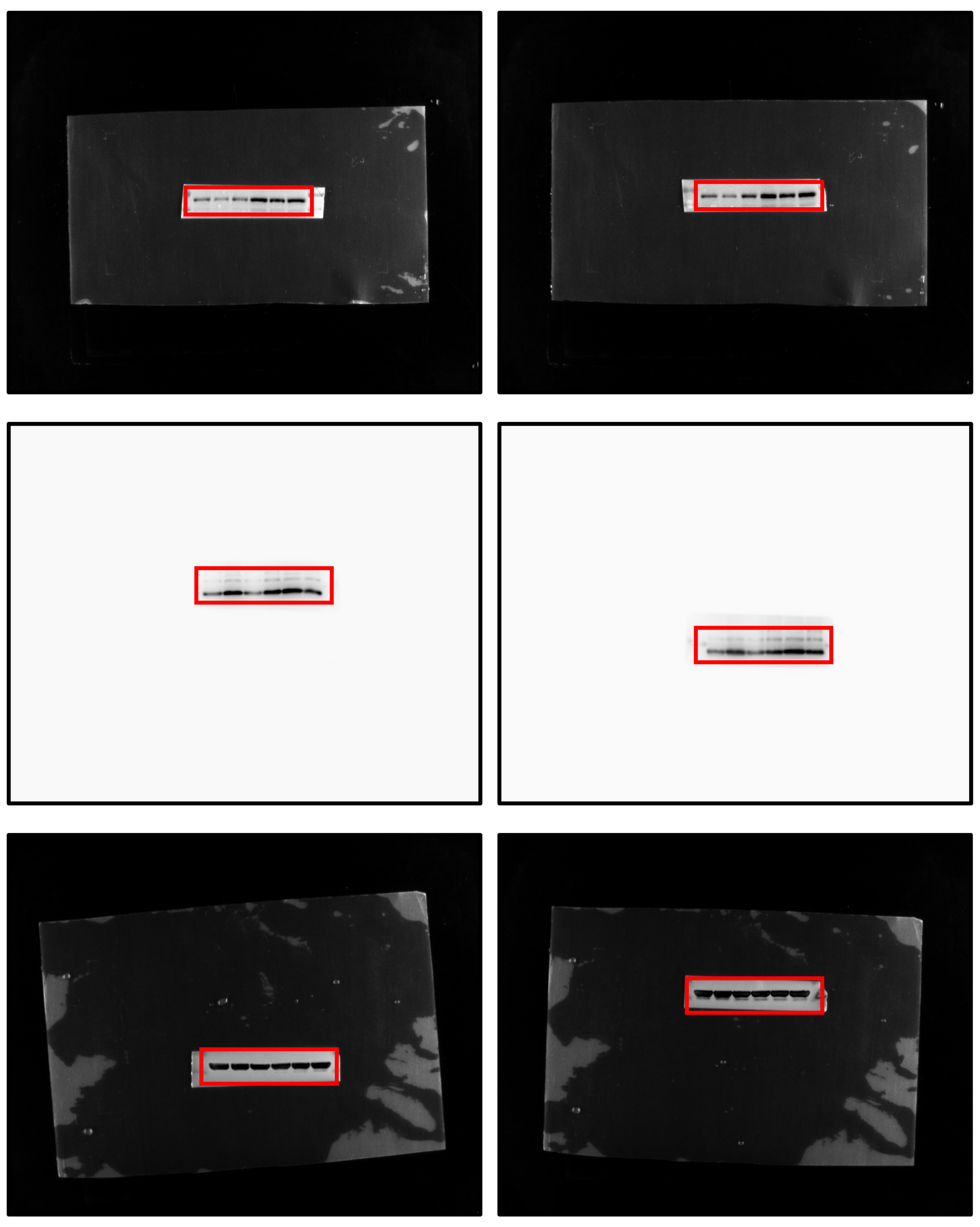

Figure 6. P

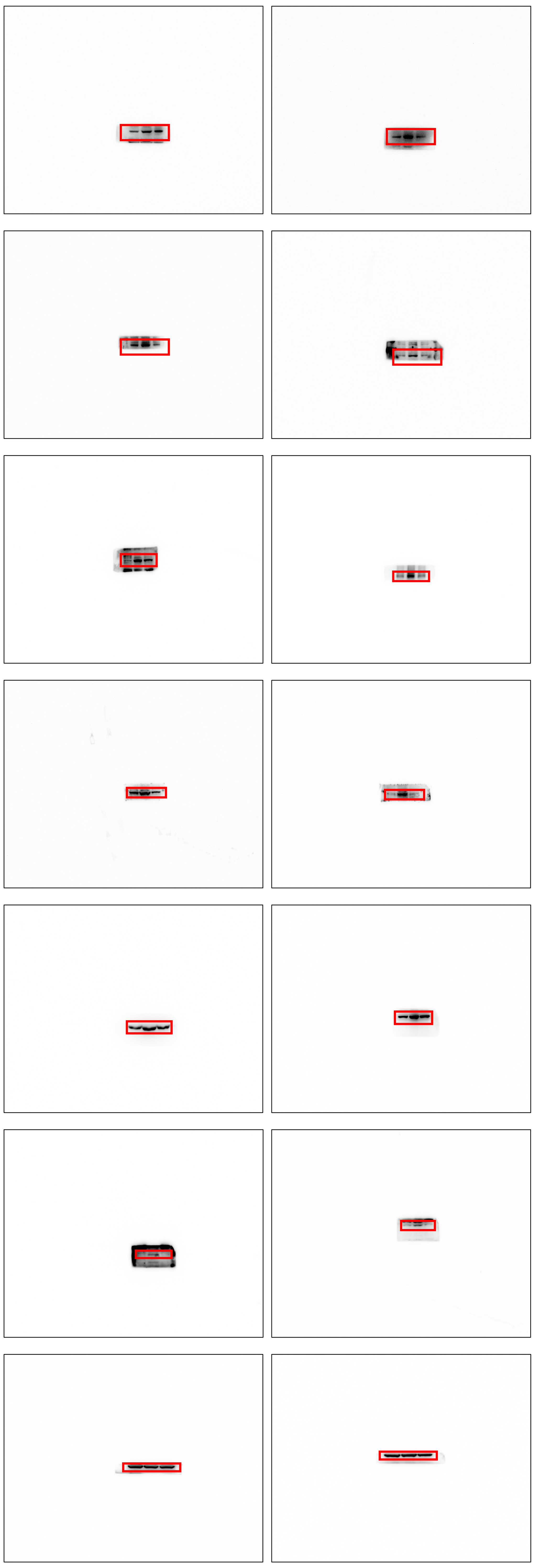

Figure 7. A

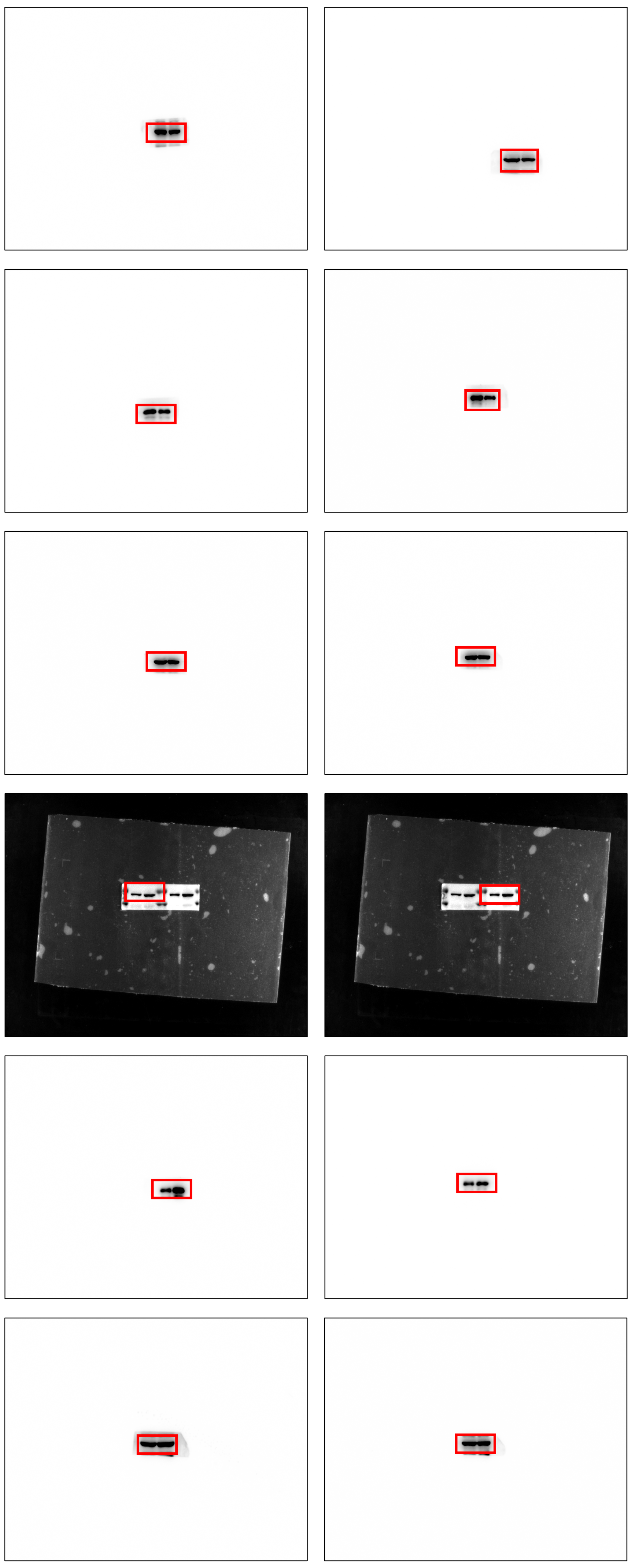

Figure 7. B

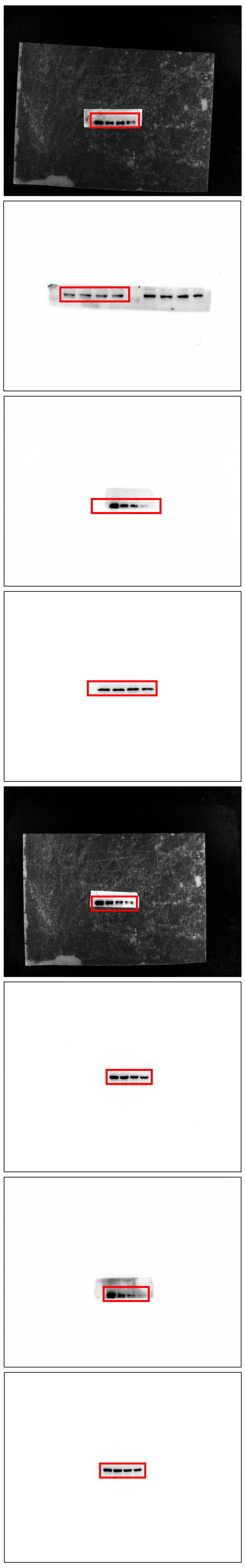

Figure 7. C

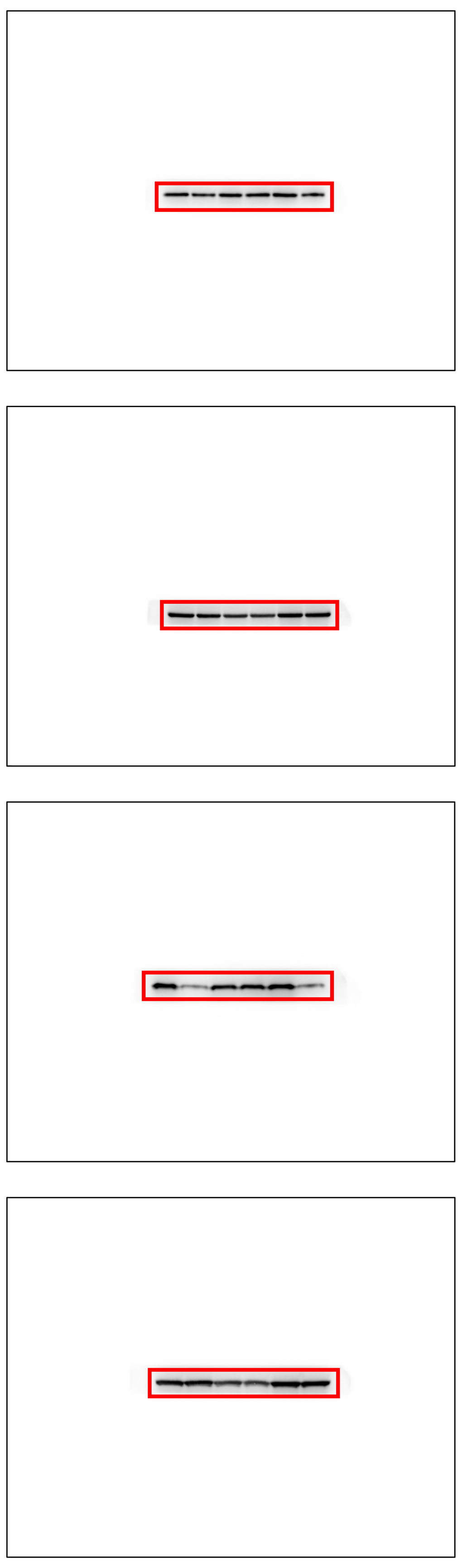

Figure 7. D

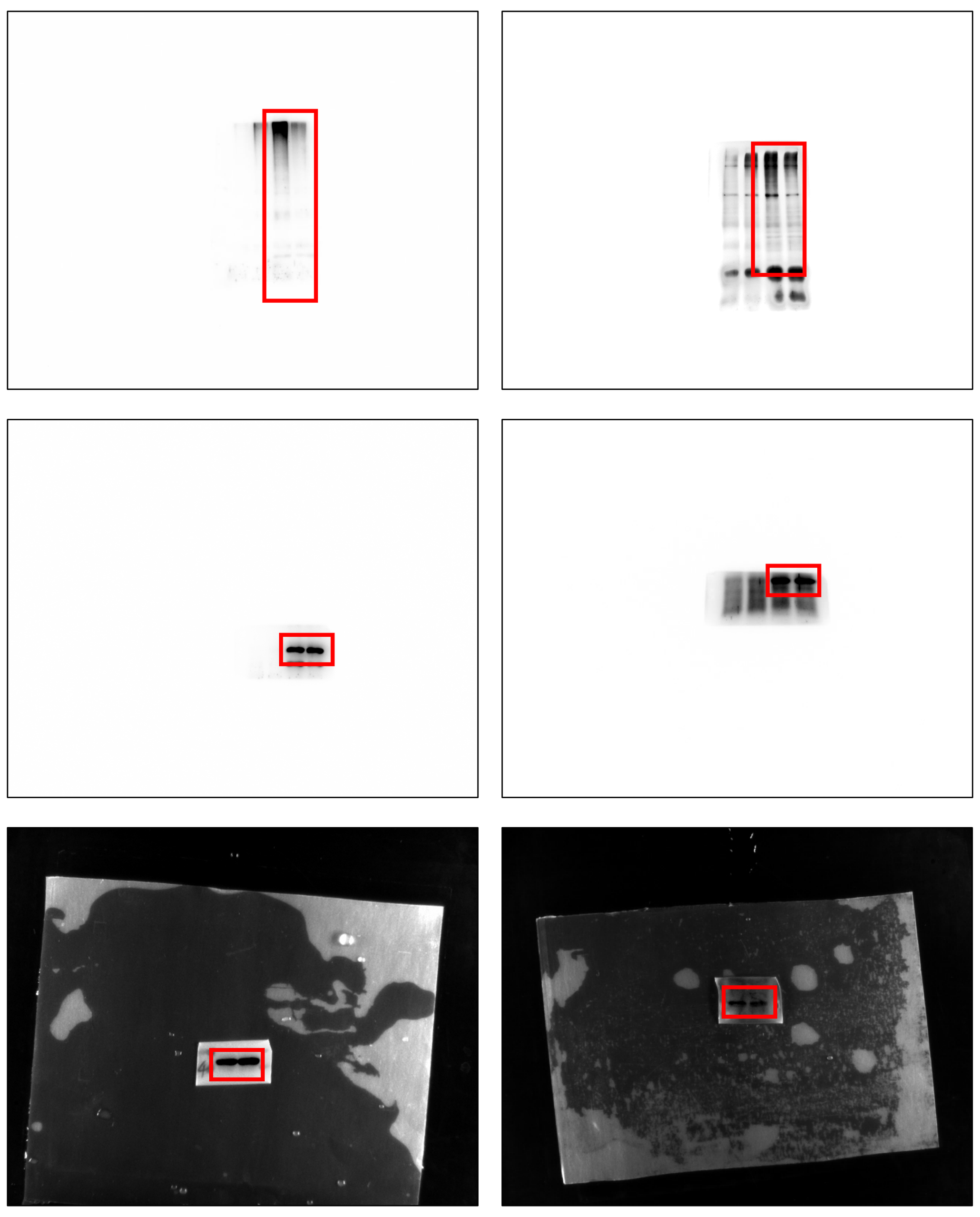

Figure 7. E

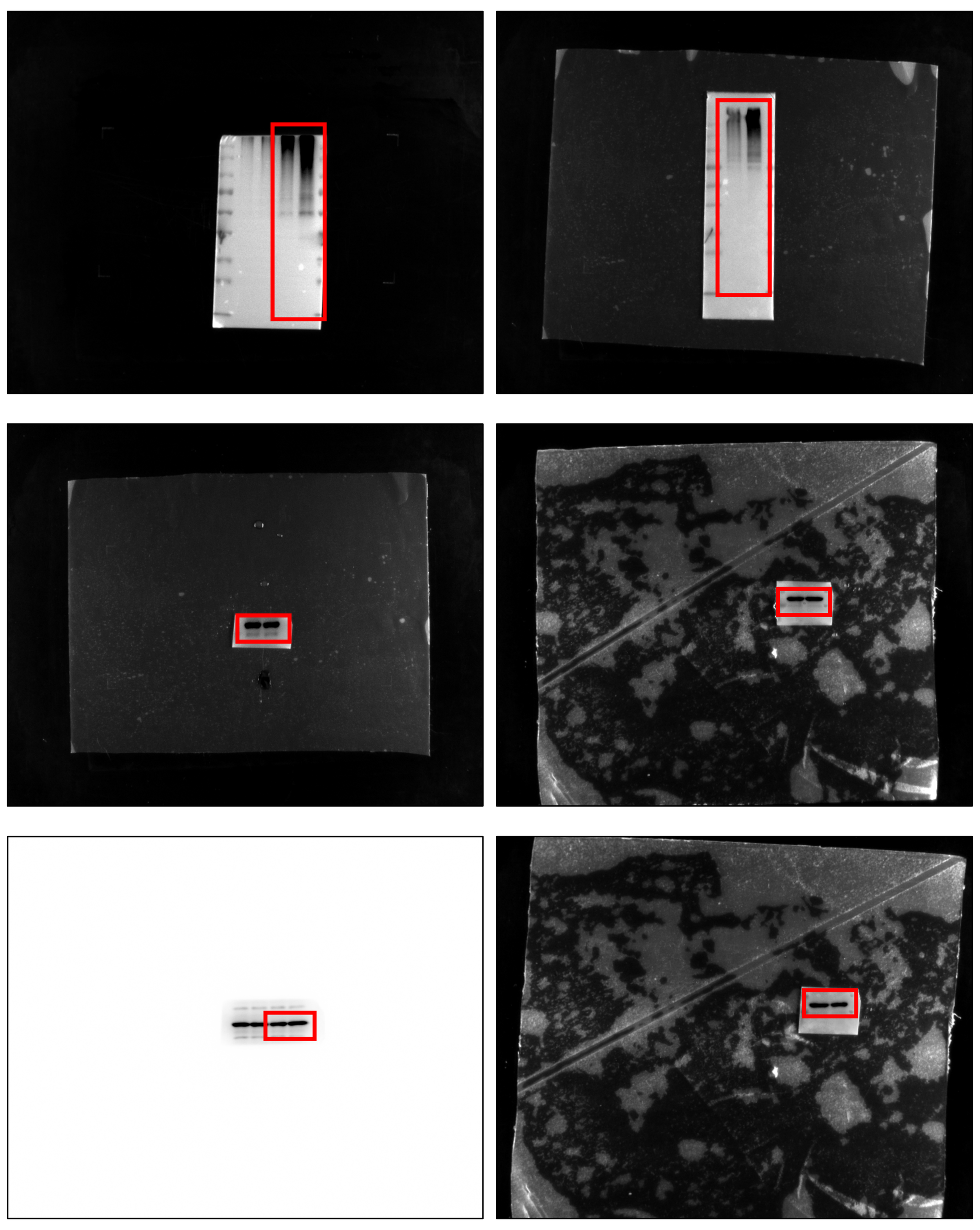

Figure 7. F

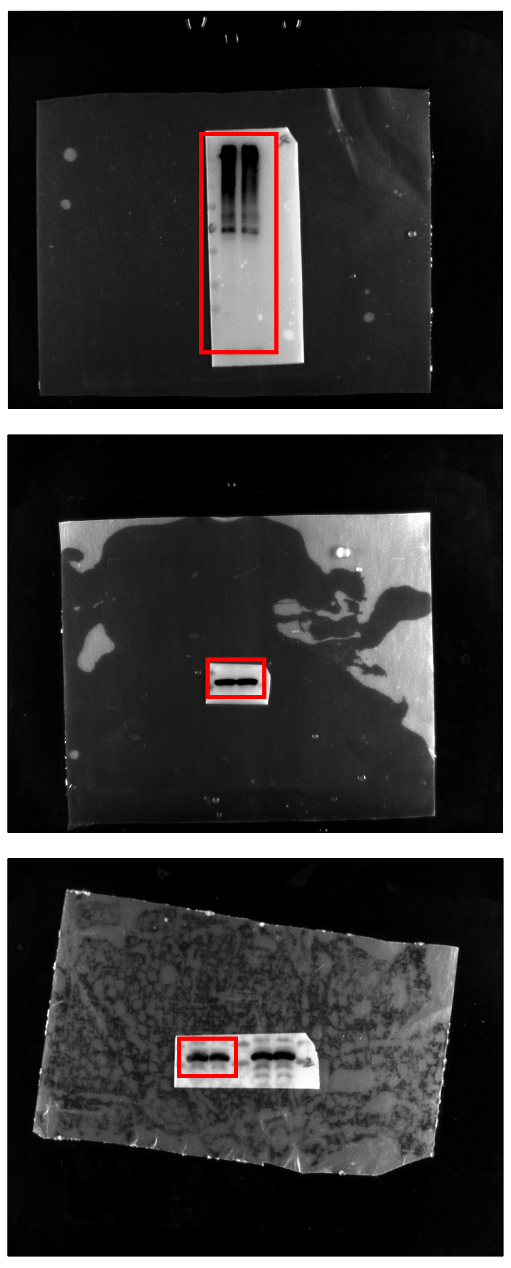

Figure 7. G

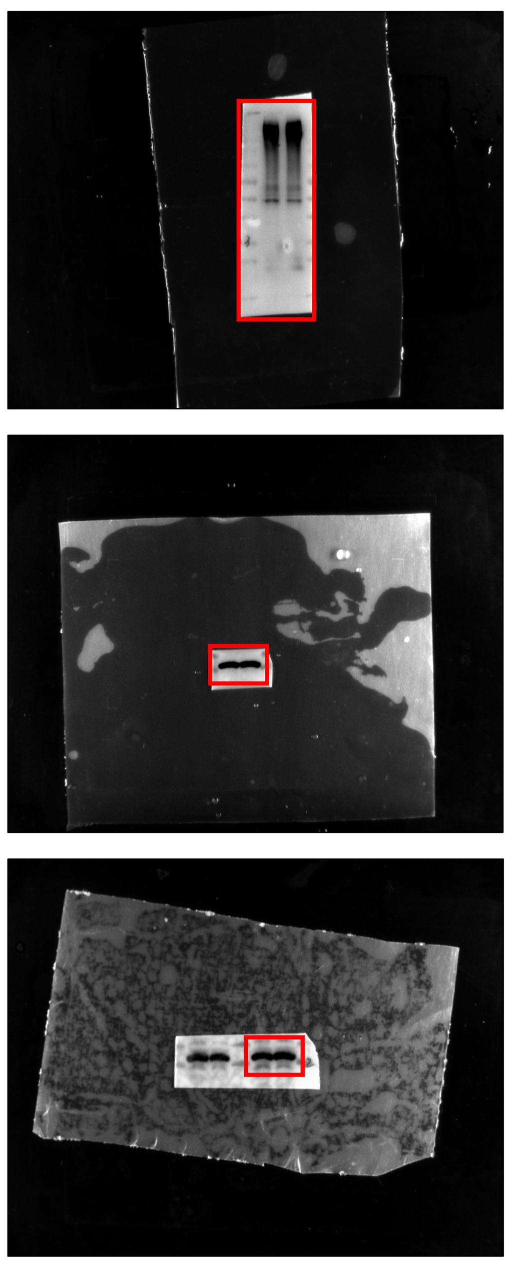

Figure 7. H

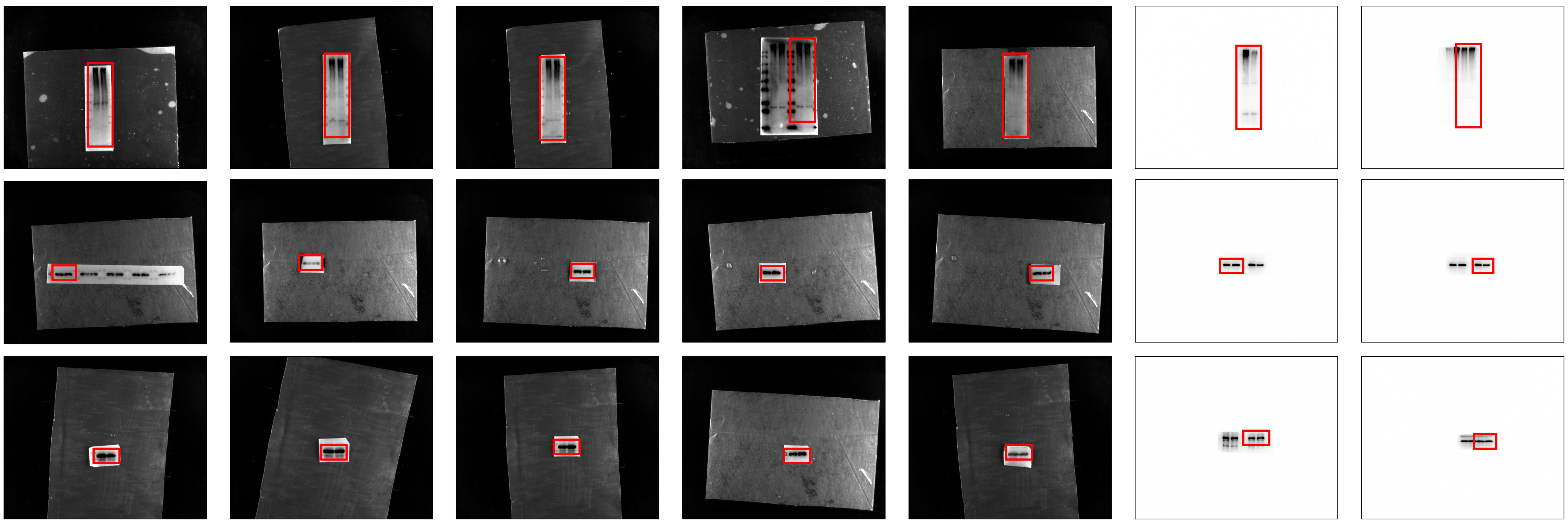

Figure 7. I

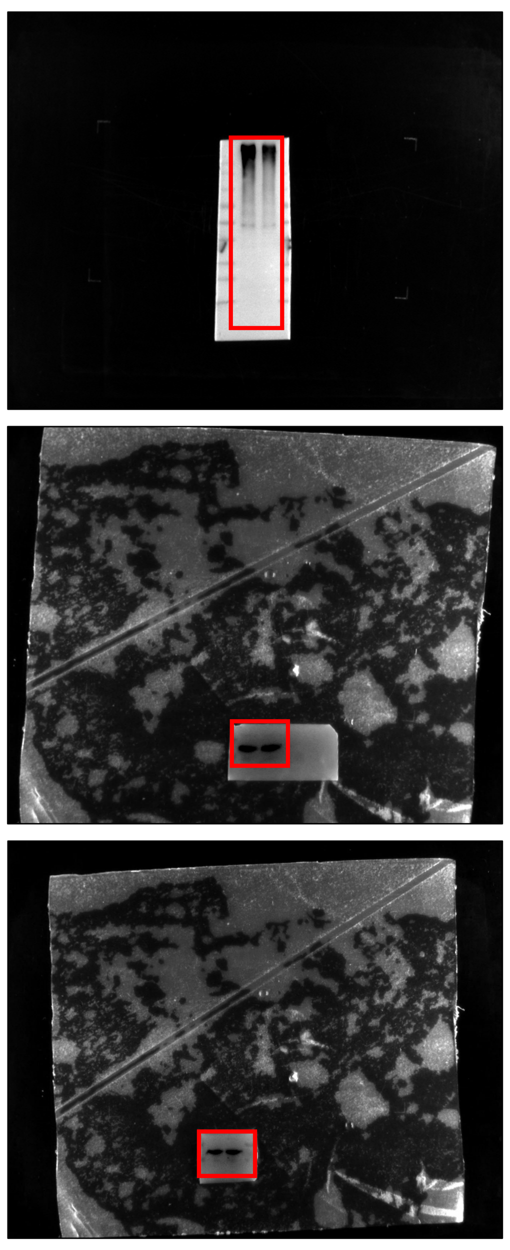

Figure 7. J

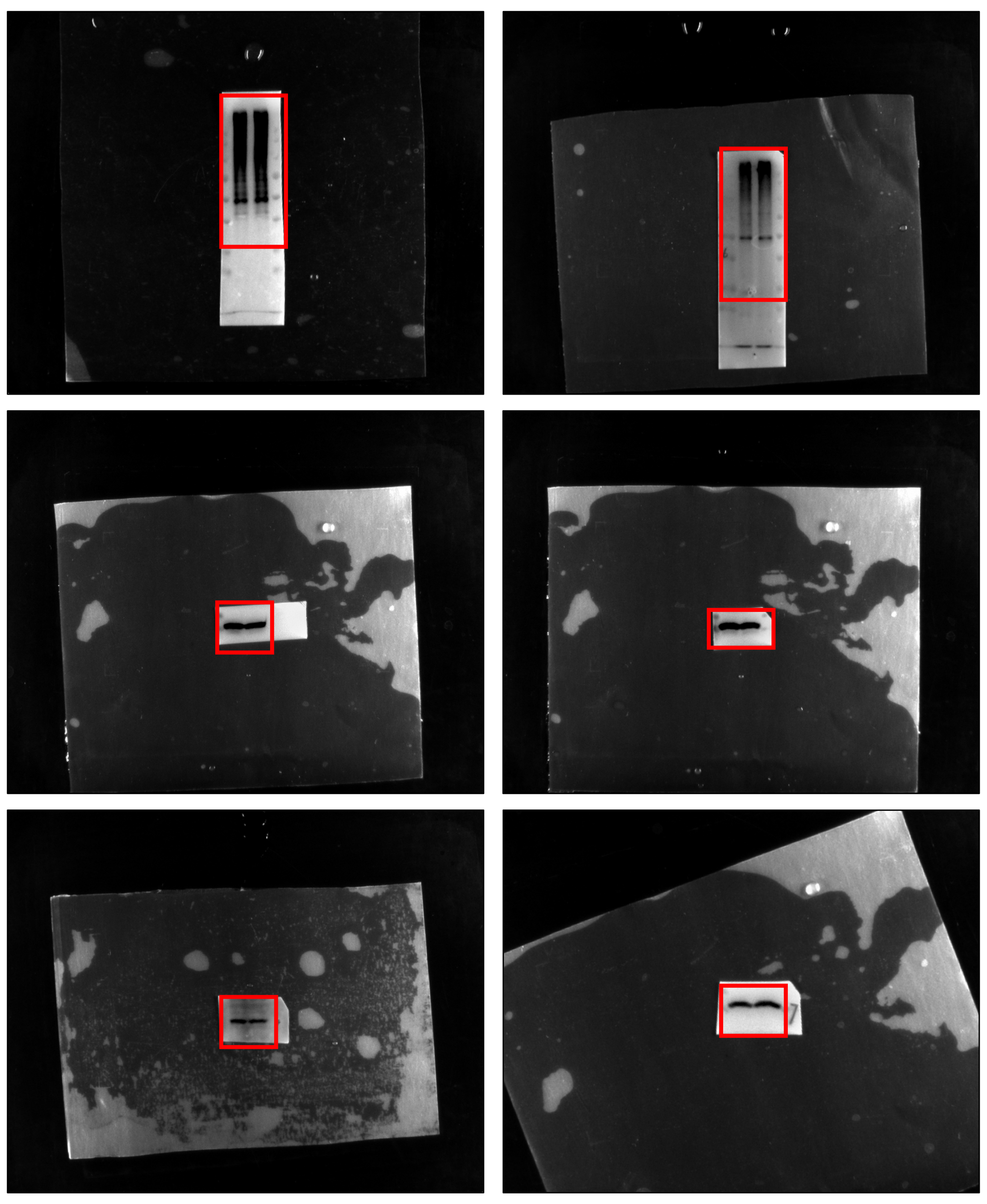

Figure 7. K

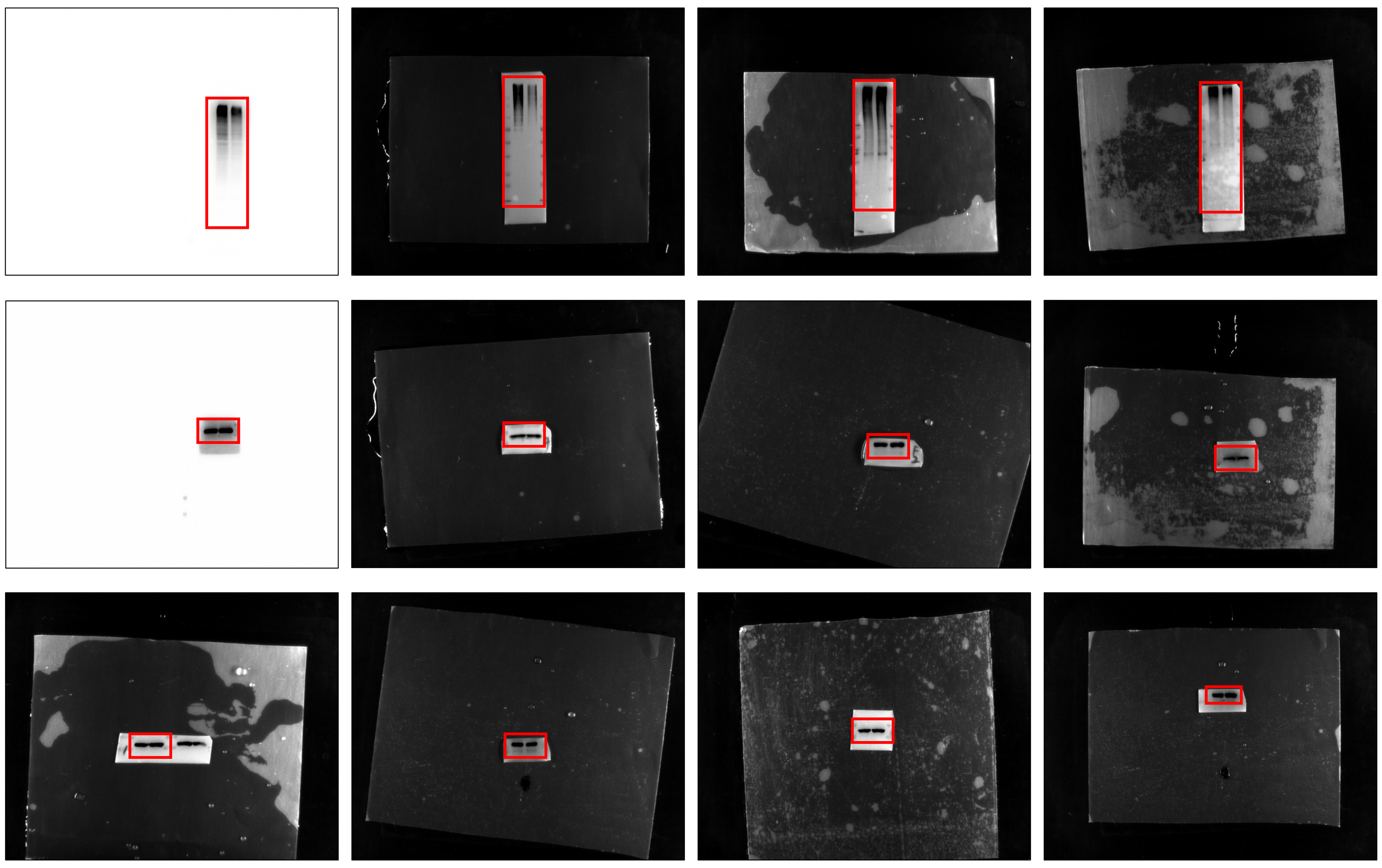

Figure 7. L

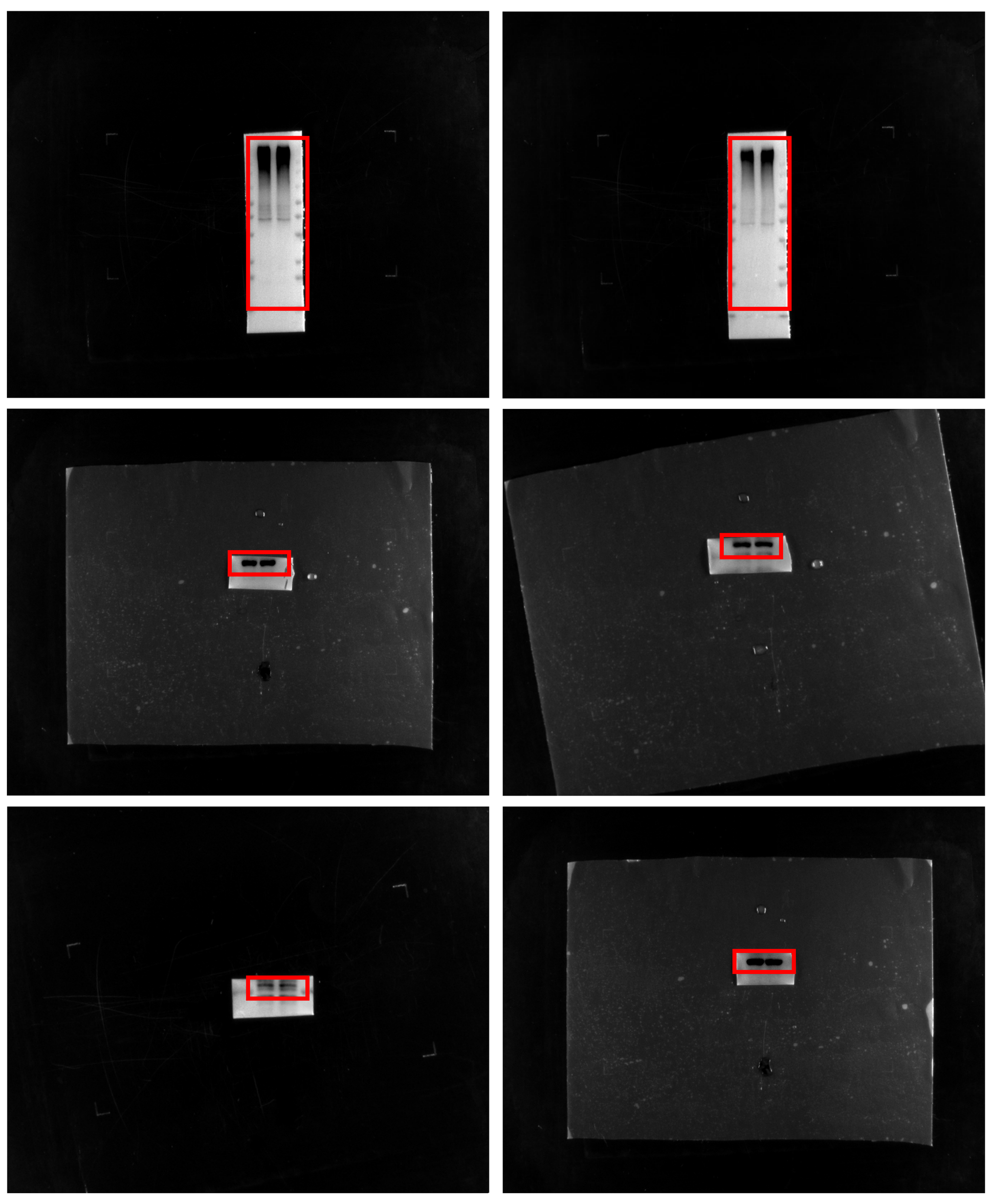

Figure S1. F

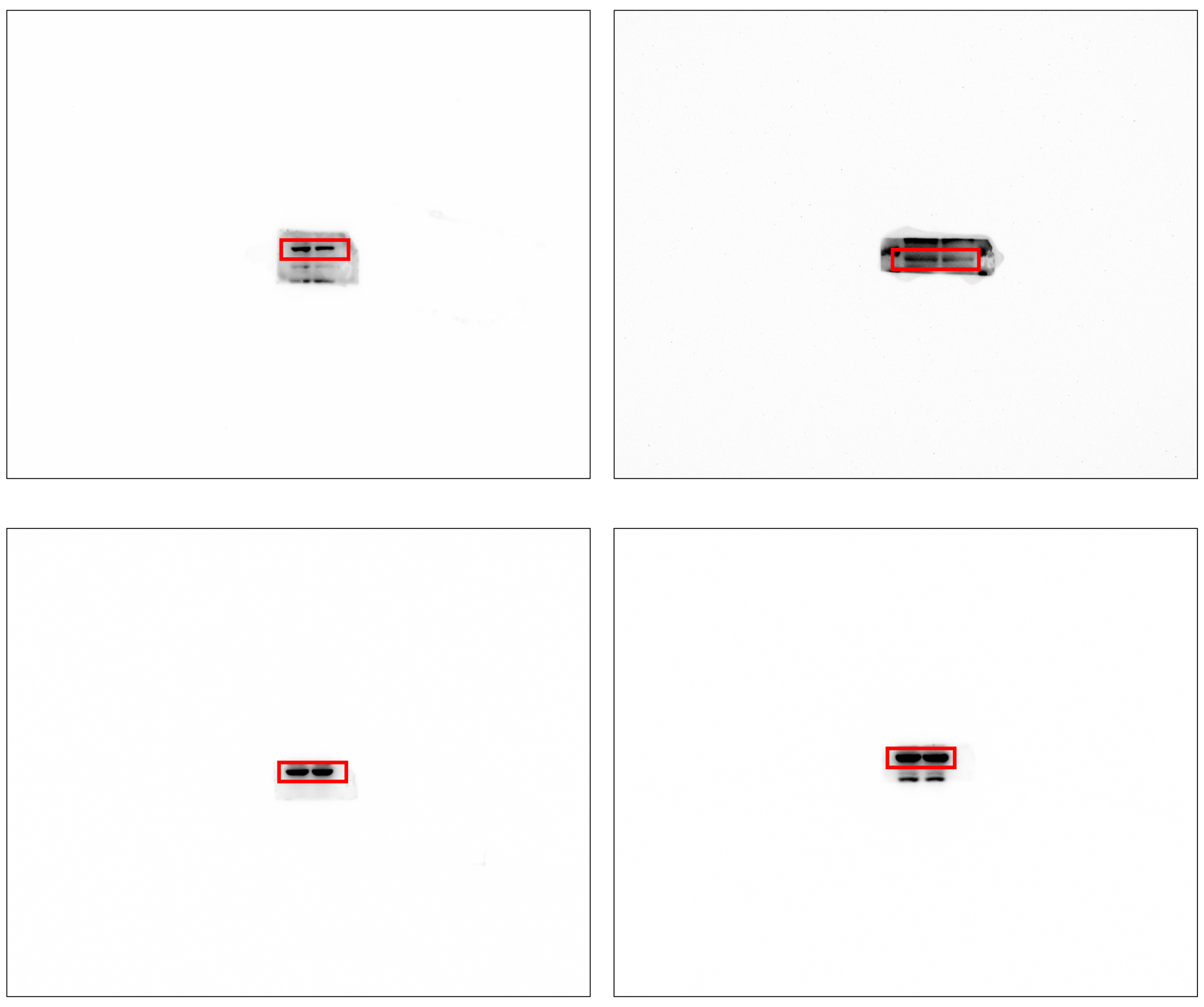

Figure S1. I

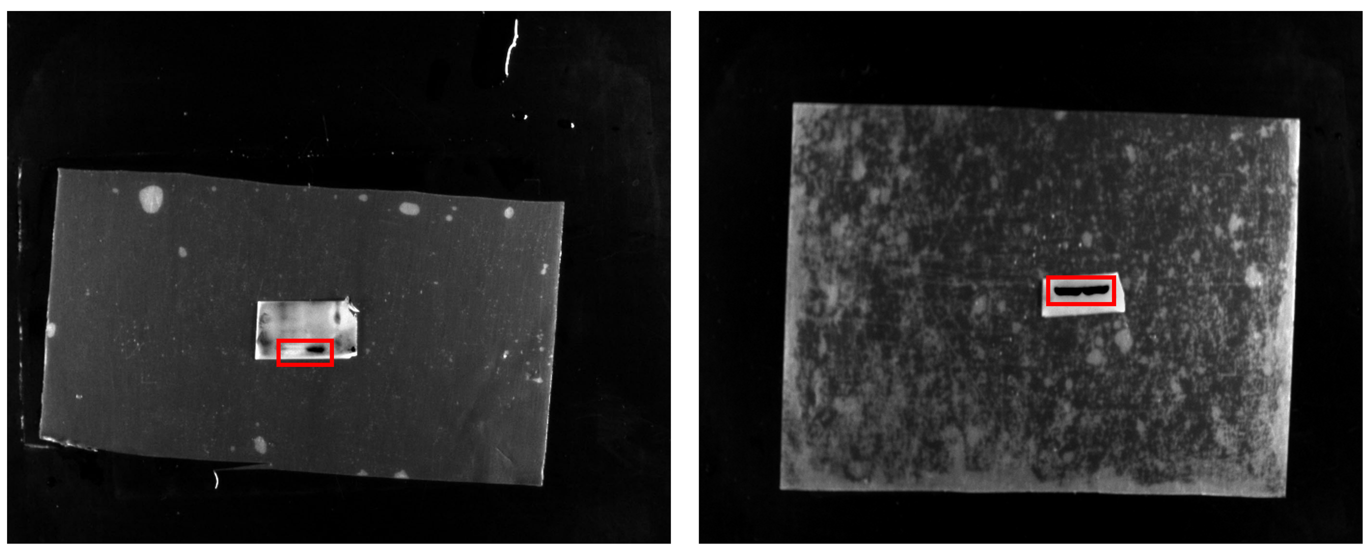

Figure S3. J

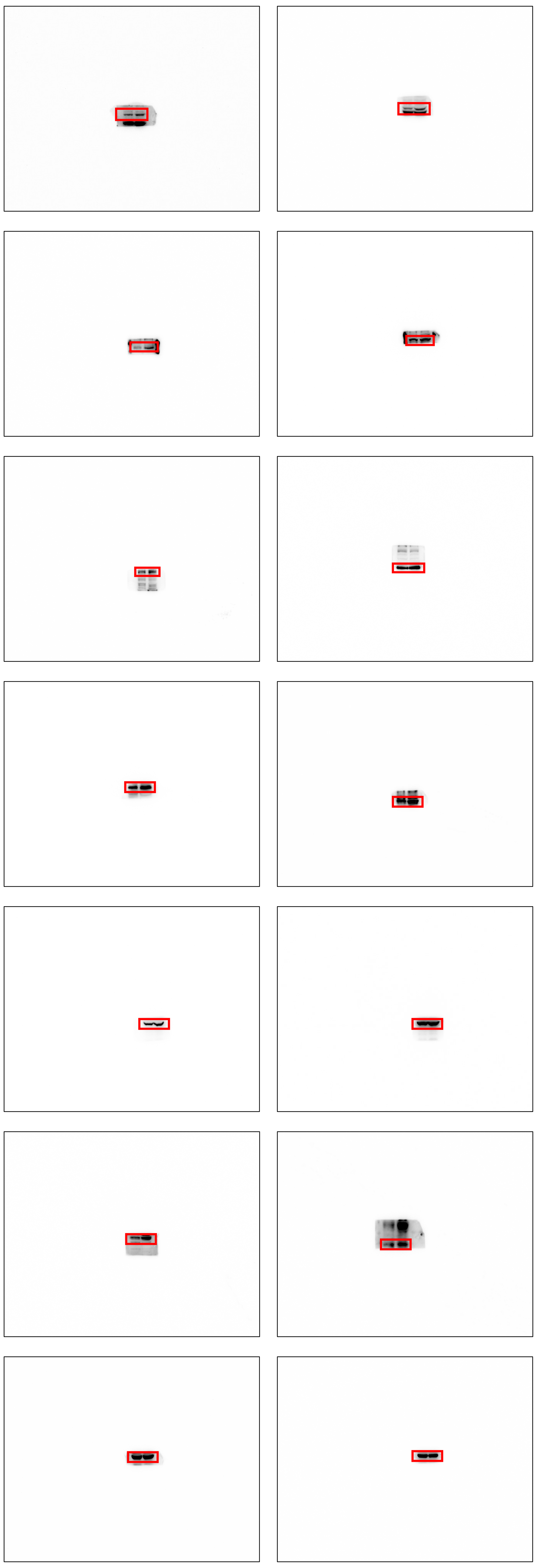

Figure S6. C

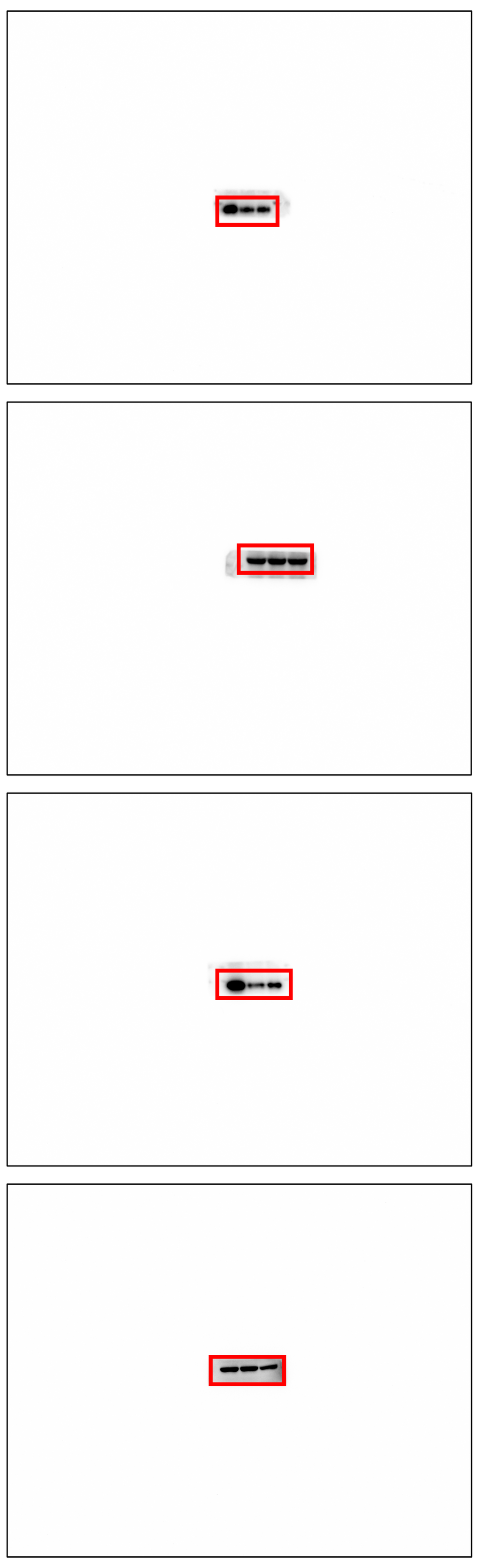

Figure S6. E

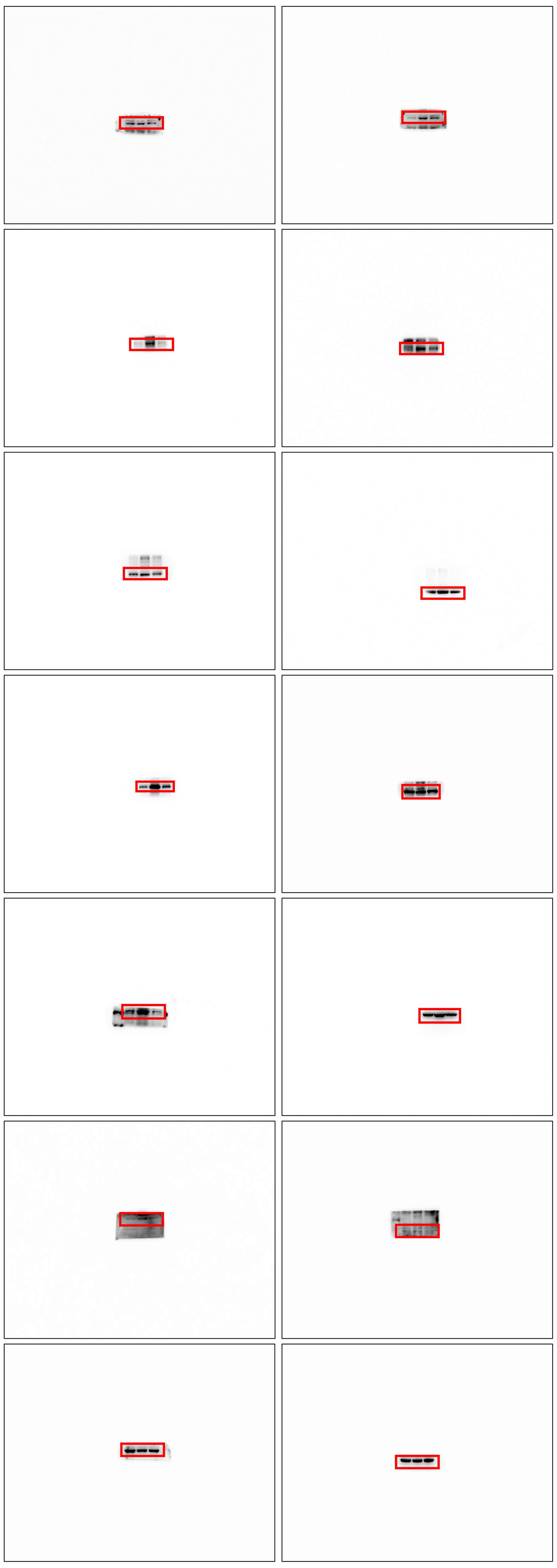

Figure S6. J

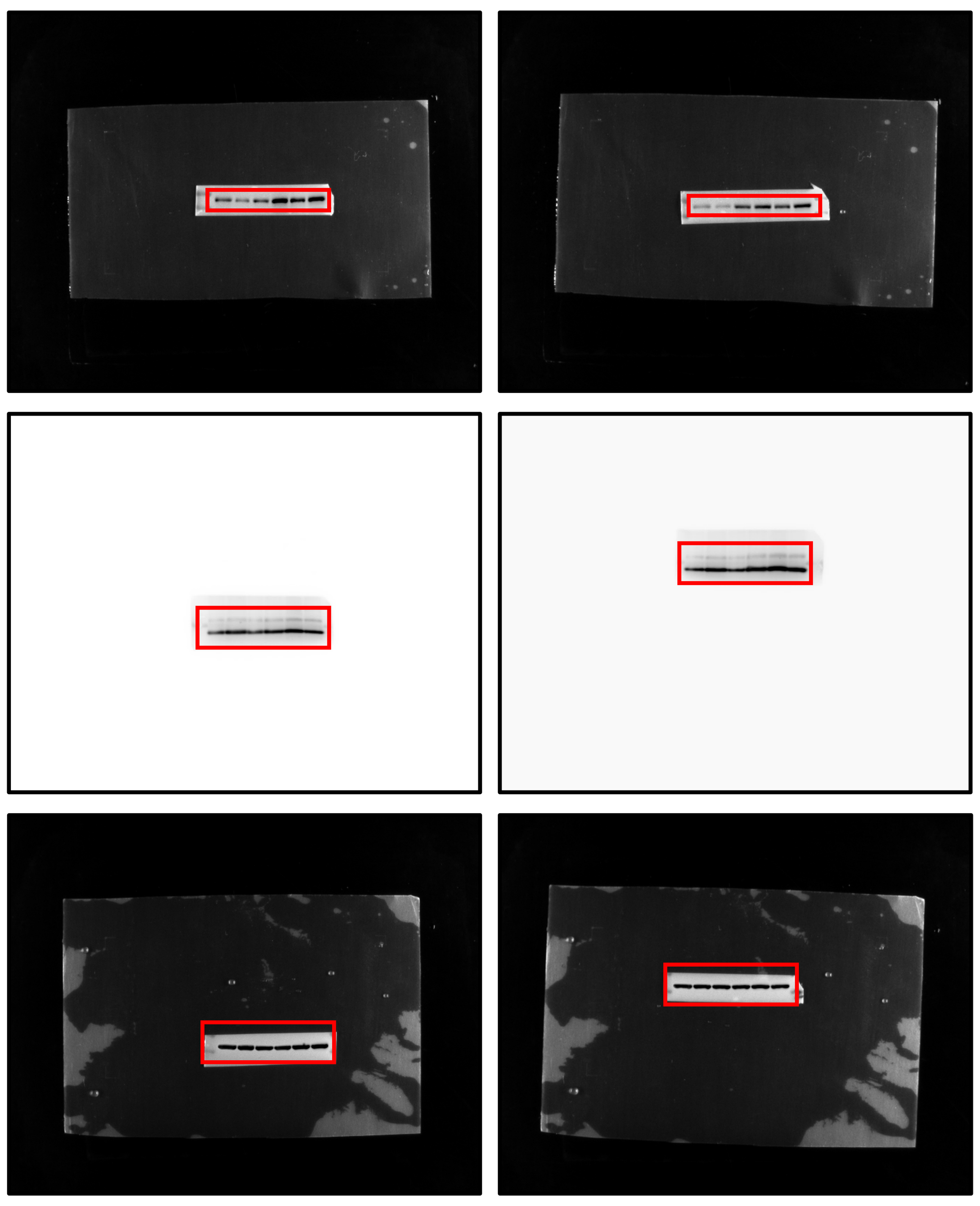

Figure S7. A

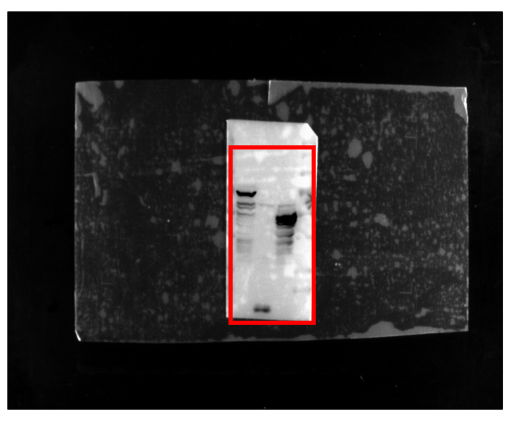

Figure S7. C

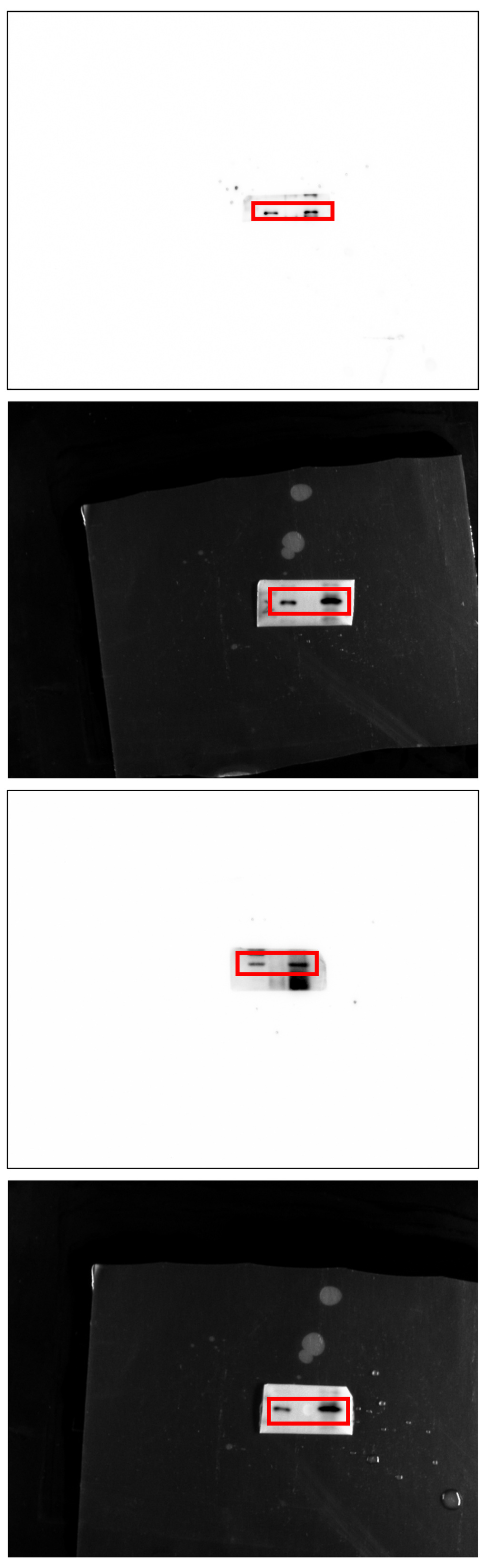

Figure S7. E

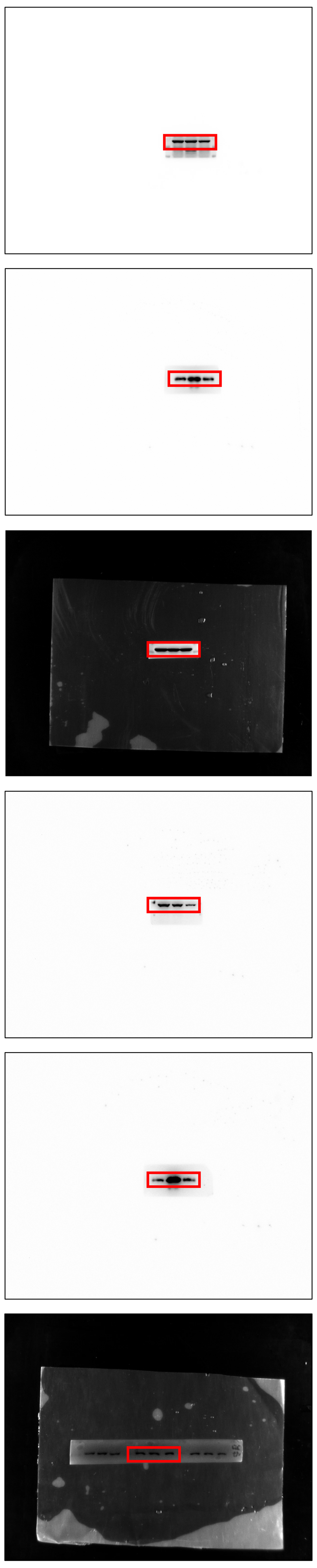

Figure S8. C

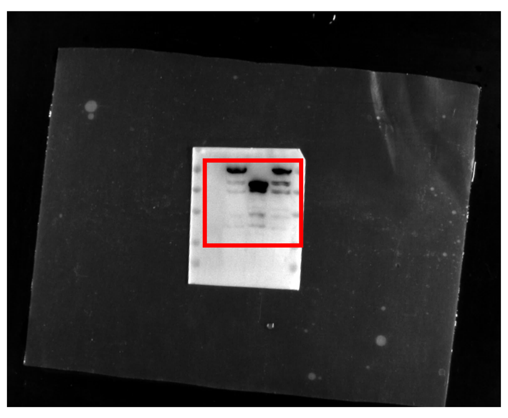

Figure S8. D

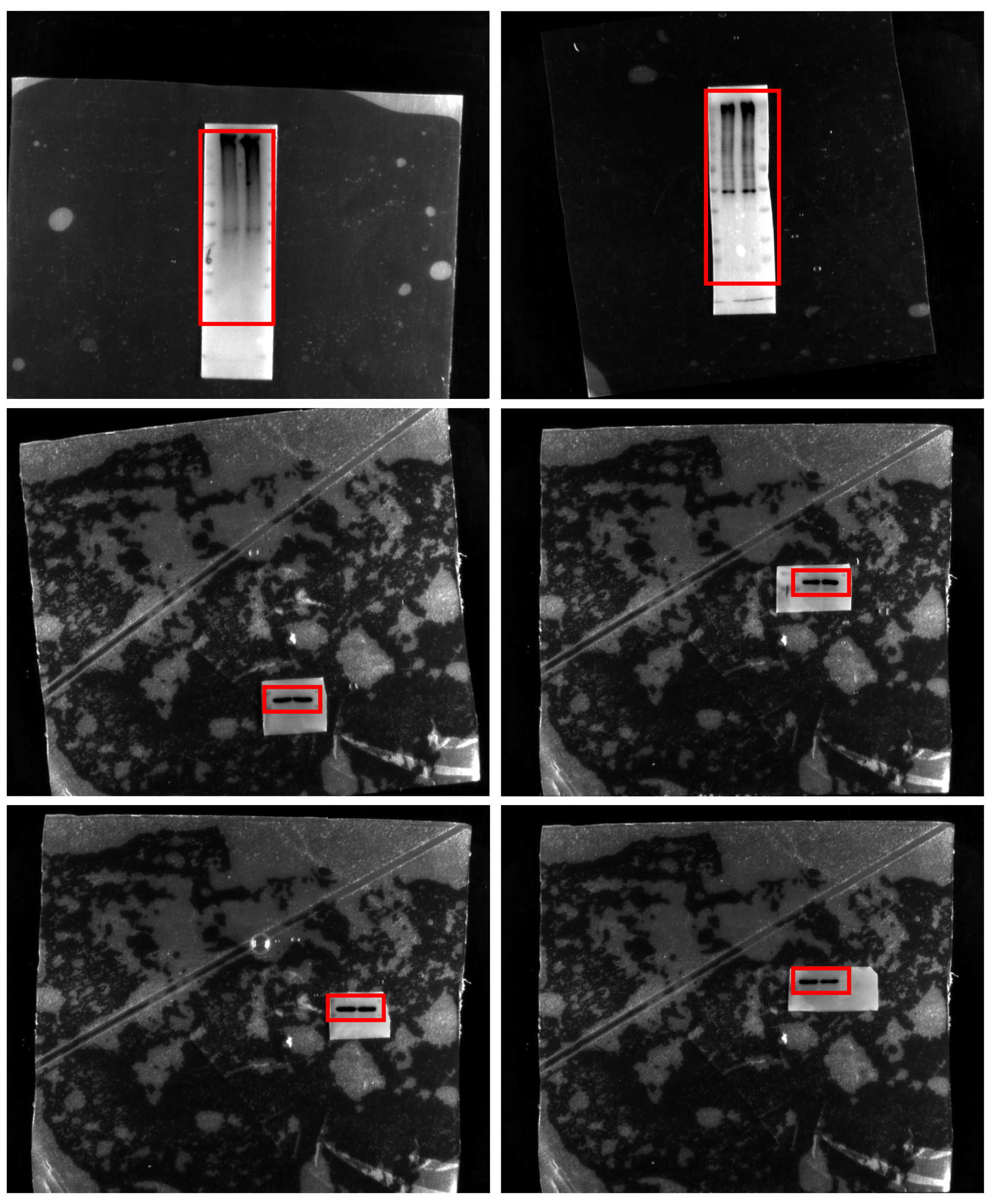

Figure S8. E

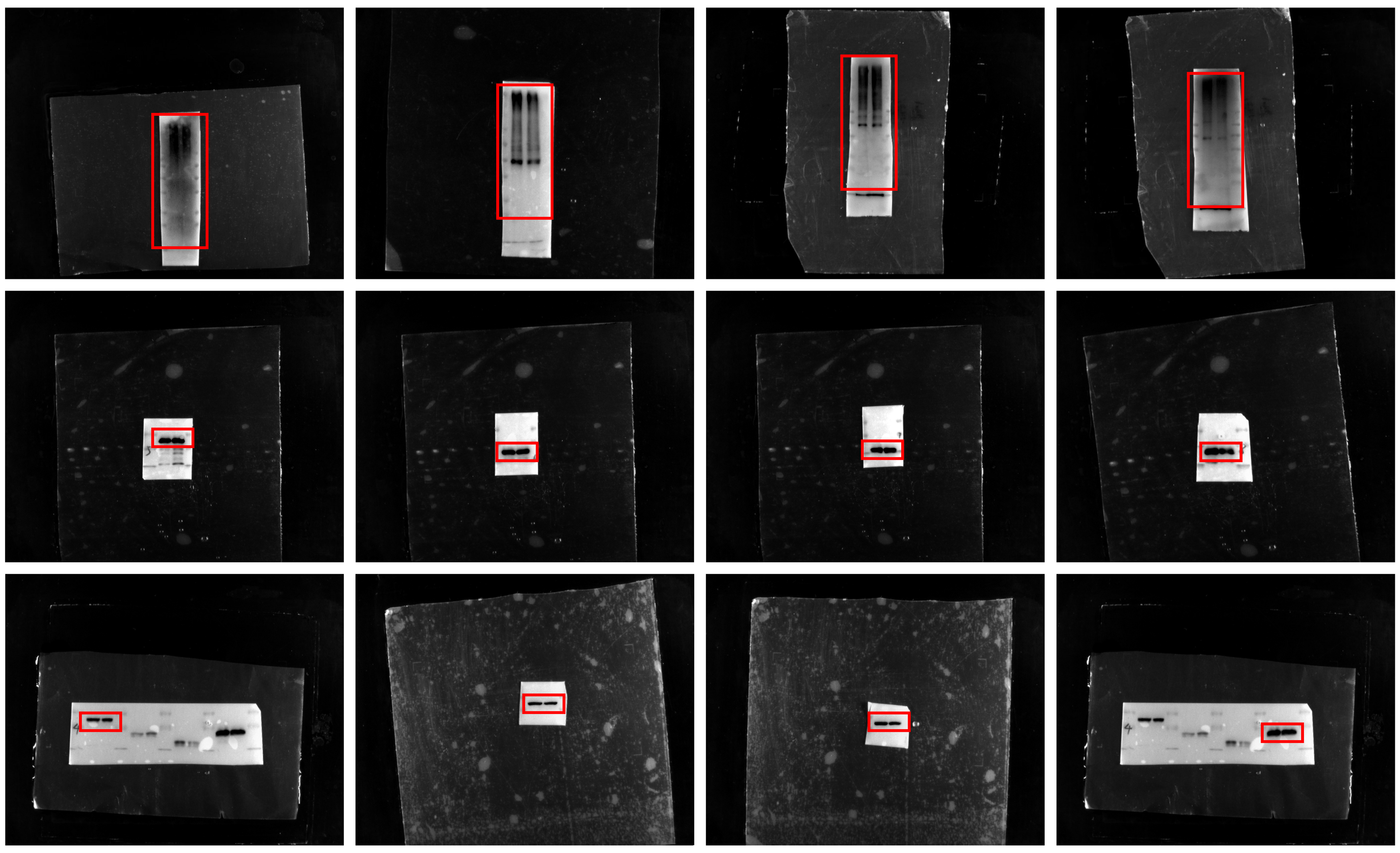

Figure S8. G

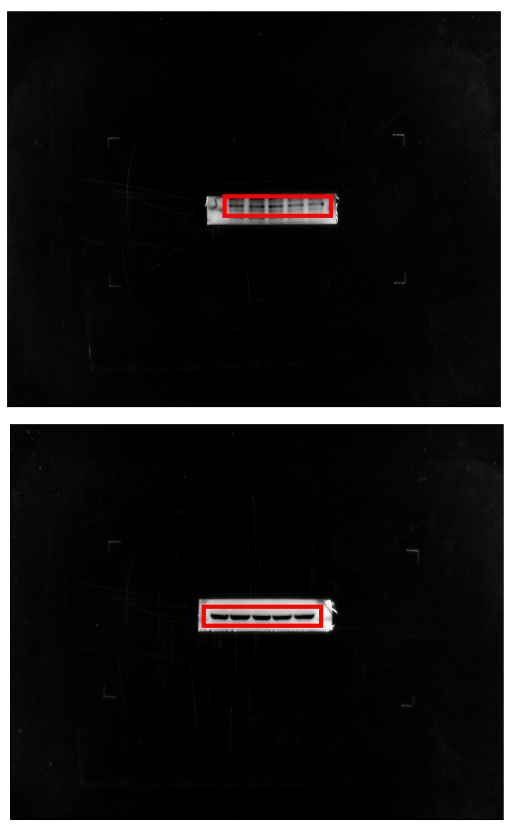

Figure S8. H

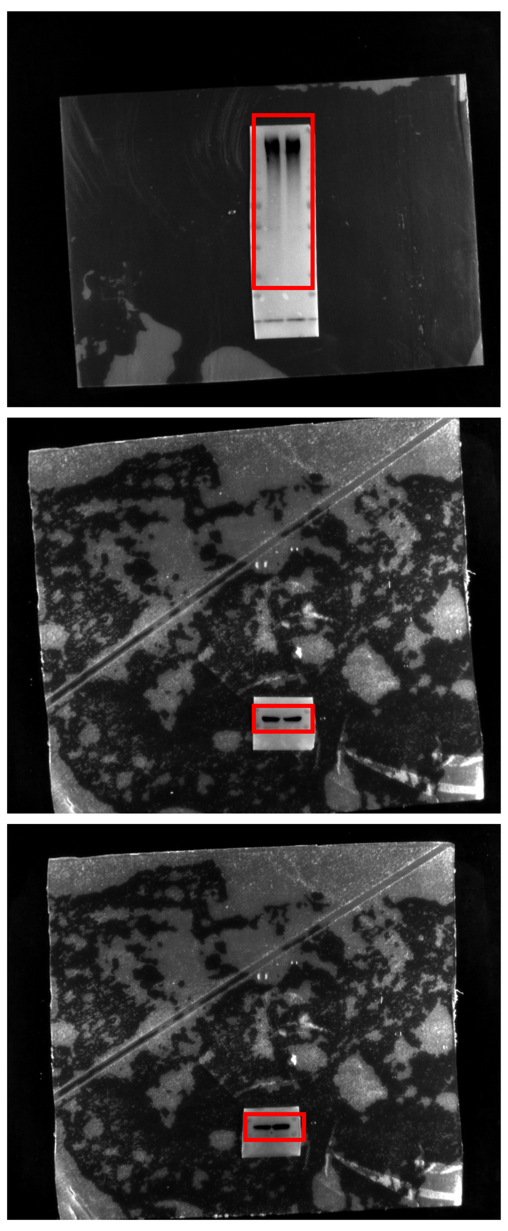

Figure S8. I

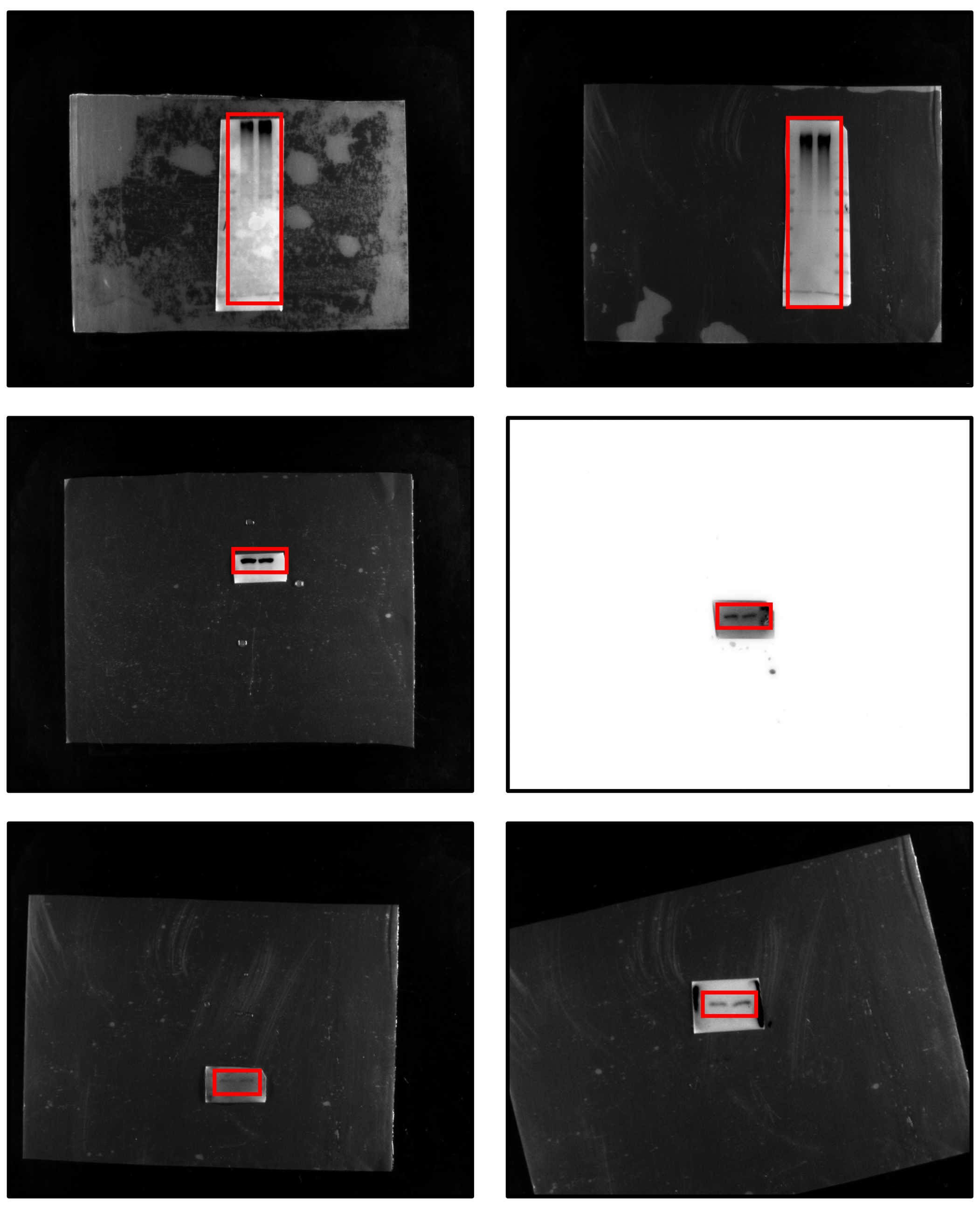

Figure S8. K

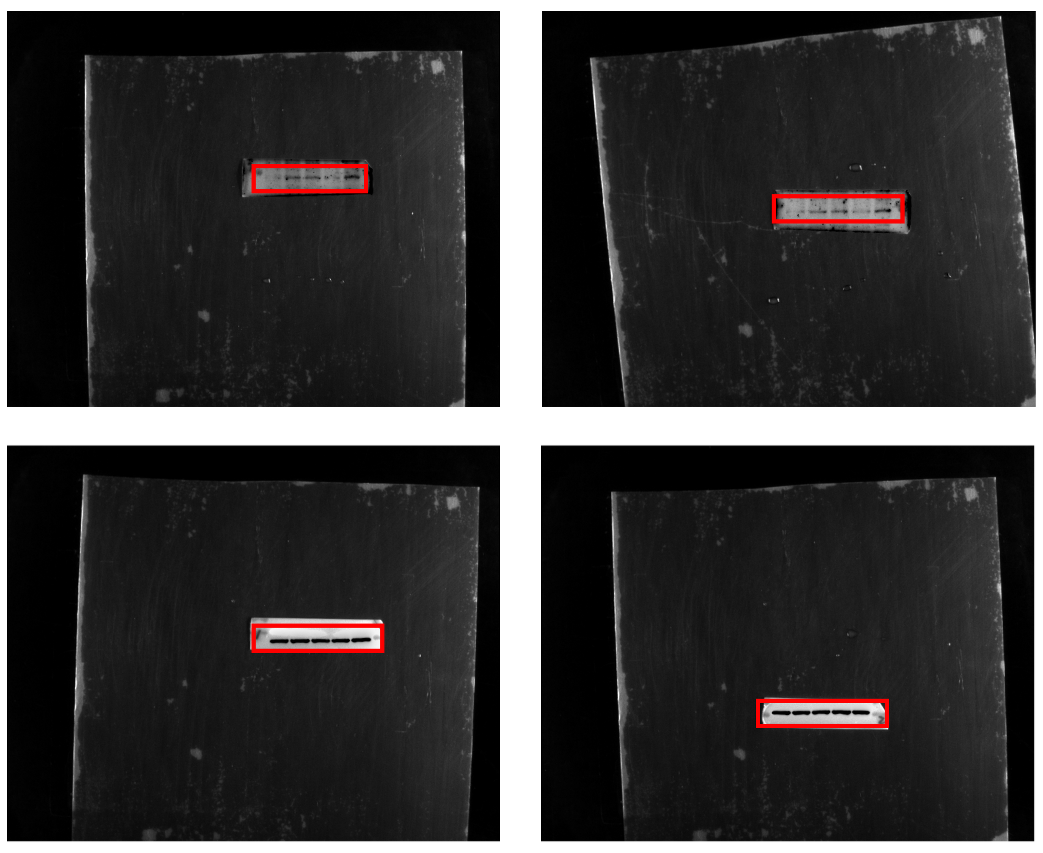

Figure S9. N

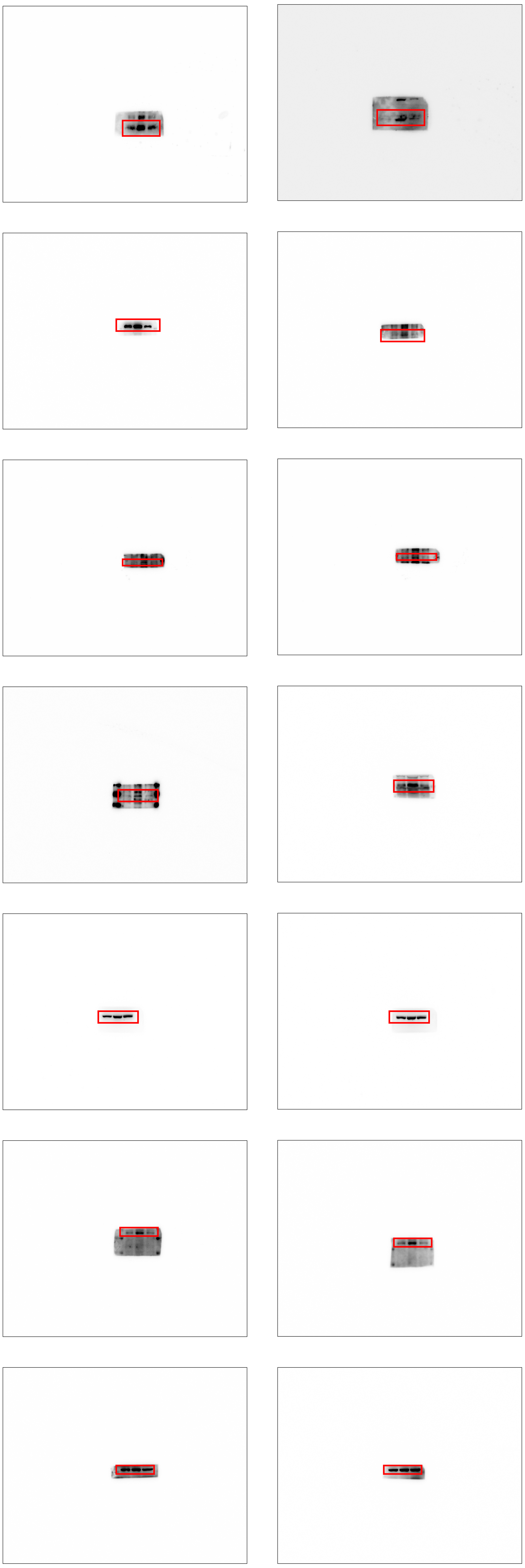

Supplement: Supplementary file 4 — Supplementary Material 4. [file 12943_2026_2580_MOESM4_ESM.pdf]
